# Physical activity and cardiometabolic risk factors in individuals with spinal cord injury: a systematic review and meta-analysis

**DOI:** 10.1007/s10654-022-00859-4

**Published:** 2022-04-07

**Authors:** Oche Adam Itodo, Joelle Leonie Flueck, Peter Francis Raguindin, Stevan Stojic, Mirjam Brach, Claudio Perret, Beatrice Minder, Oscar H. Franco, Taulant Muka, Gerold Stucki, Jivko Stoyanov, Marija Glisic

**Affiliations:** 1 Swiss Paraplegic Research, Guido A. Zäch Str. 1, 6207 Nottwil, Switzerland; 2 Graduate School for Health Sciences, University of Bern, Mittelstrasse 43, 3012 Bern, Switzerland; 3 Sports Medicine, Swiss Paraplegic Centre Nottwil, 6207 Nottwil, Switzerland; 4 Institute of Social and Preventive Medicine (ISPM), University of Bern, Mittelstrasse 43, 3012 Bern, Switzerland; 5 Public Health and Primary Care Library, University Library of Bern, University of Bern, Bern, Switzerland

**Keywords:** Spinal cord injury, Physical activity, Exercise, Cardiovascular diseases, Cardiac function

## Abstract

Physical inactivity in individuals with spinal cord injury (SCI) has been suggested to be an important determinant of increased cardiometabolic disease (CMD) risk. However, it remains unclear whether physically active SCI individuals as compared to inactive or less active individuals have truly better cardiometabolic risk profile. We aimed to systematically review and quantify the association between engagement in regular physical activity and/or exercise interventions and CMD risk factors in individuals with SCI. Four medical databases were searched and studies were included if they were clinical trials or observational studies conducted in adult individuals with SCI and provided information of interest. The Grading of Recommendations Assessment, Development and Evaluation (GRADE) approach was applied to rate the certainty of evidence. Of 5816 unique citations, 11 randomized clinical trials, 3 non-randomized trial and 32 cross-sectional studies comprising more than 5500 SCI individuals were included in the systematic review. In meta-analysis of RCTs and based on evidence of moderate certainty, physical activity in comparison to control intervention was associated with: (i) better glucose homeostasis profile [WMD of glucose, insulin and Assessment of Insulin Resistance (HOMA-IR) were − 3.26 mg/dl (95% CI − 5.12 to − 1.39), − 3.19 μU/ml (95% CI − 3.96 to − 2.43)] and − 0.47 (95% CI − 0.60 to − 0.35), respectively], and (ii) improved cardiorespiratory fitness [WMD of relative and absolute oxygen uptake relative (VO_2_) were 4.53 ml/kg/min (95% CI 3.11, 5.96) and 0.26 L/min (95% CI 0.21, 0.32) respectively]. No differences were observed in blood pressure, heart rate and lipids (based on evidence of low/moderate certainty). In meta-analysis of cross-sectional studies and based on the evidence of very low to low certainty, glucose [WMD − 3.25 mg/dl (95% CI − 5.36, − 1.14)], insulin [− 2.12 μU/ml (95% CI − 4.21 to − 0.03)] and total cholesterol [WMD − 6.72 mg/dl (95% CI − 13.09, − 0.34)] were lower and HDL [WMD 3.86 mg/dl (95% CI 0.66, 7.05)] and catalase [0.07 UgHb-1 (95% CI 0.03, 0.11)] were higher in physically active SCI individuals in comparison to reference groups. Based on limited number of cross-sectional studies, better parameters of systolic and diastolic cardiac function and lower carotid intima media thickness were found in physically active groups. Methodologically sound clinical trials and prospective observational studies are required to further elaborate the impact of different physical activity prescriptions alone or in combination with other life-style interventions on CMD risk factors in SCI individuals.

**Supplementary Information:**

The online version contains supplementary material available at 10.1007/s10654-022-00859-4.

## Introduction

Spinal cord injury (SCI) leads to a long-term disability, as a consequence of substantial motor, sensory, or autonomic damage below the level of the lesion [1–3]. Impairments in physical functioning, environmental and psychological barriers typically affect the engagement in physical activity after the injury and approximately 50% of individuals with SCI are engaged in inactive lifestyle (as compared to around a quarter of adults in general population) [4–7]. Reduced physical activity coupled with loss of somatic and autonomic control in SCI cause reduced cardiorespiratory fitness, detrimental changes in body composition and metabolic profile and lead to worsening of cardiometabolic disease (CMD) risk profile following the SCI [8–12]. In addition, autonomic dysfunction alters blood pressure and heart rate regulation, while prolonged bed rest contributes to left ventricle (LV) remodelling and impaired systolic and diastolic cardiac function that were also linked with increased CVD risk [13, 14].

Emerging evidence suggested improvements in cardiorespiratory fitness, systolic cardiac function and more advantageous cardiometabolic risk profile in physically active individuals with SCI, yet, reports on minimal physical activity engagement necessary to improve CVD risk profile remained inconsistent [15–21]. Thus, over the past decade a few systematic reviews of the literature aimed to synthetize the available evidence and inform development of the SCI-specific exercise guidelines [22–26]. These guidelines reported cardiometabolic health benefits with engagement in physical activity ranging from 40 to 150 min of physical activity per week depending on type of the exercise regimes and specific cardiometabolic risk factors [27–29].

The most comprehensive overview of the literature was published by van der Scheer et al. in 2017 [23] and created the basis for development of scientific guidelines that specify the type and minimum prescription of exercise required to improve fitness and cardiometabolic health in adults with SCI [30]. Cardiorespiratory fitness improvements were suggested with engagement in at least 20 min of moderate to vigorous intensity aerobic exercise twice per week and three sets of strength exercise for each major functioning muscle group at a moderate to vigorous intensity two times per week [29]. Whereas, cardiometabolic health benefits were suggested with engagement in at least 30 min of moderate to vigorous intensity aerobic exercise three times per week [29].

However, moderate or vigorous intensity exercise may not be reachable by all SCI individuals, and in particular to individuals with injury level above the sixth thoracic segment (due to small remaining muscle muss under voluntary control and disruption of autonomic nervous system outflow) [31] . Hence, while SCI individuals may meet the advised weekly exercise hours some may not be able to reach the 40–59% of peak oxygen uptake that is considered a bare minimum to observe health benefits [31]. Similarly, individuals who meet the recommended relative exercise intensity may achieve up to fourfold lower energy expenditure as compared to able-bodied individuals [32]. Thus, it remains unclear whether SCI individuals who are considered physical active (e.g., who meet the physical activity guidelines) as compared to individuals who are considered inactive or sedentary (e.g., do not meet physical activity recommendations) have truly better CMD risk profile.

To our knowledge, only a single meta-analysis [24] explored the differences in inflammation markers between physically active vs. inactive SCI individuals; while none of other reviews attempted to quantitatively summarize the evidence in the field. The main reasoning for such decisions was similar across reviews and referred to: (i) methodological differences in study designs; (ii) high clinical heterogeneity among SCI population or (ii) variations in baseline levels of physical activity participation. SCI population heterogeneity is driven by variations in aetiology (traumatic and non-traumatic) or severity of the injury (level and completeness) and injury duration as well as differences in traditional demographic factors such as age, gender, comorbidities and secondary health conditions. However, considering that original studies provide rather simple statistical comparisons between active and inactive groups, with increasing number of publications in the field, methods such as meta-regression could help us understand how these personal and SCI-specific factors affect the association between physical activity and CMD risk factors. In addition, the inclusion of heterogenous study samples and individuals with varying baseline levels of exercise/physical activity participation could increase the generalizability and robustness of the conclusions and subsequent recommendations.

Hence, this systematic review aimed to address the following research questions: (1) Whether SCI individuals who are engaged in habitual/regular physical activity of any level (e.g., meet or exceed physical activity recommendations of at least 30 min of moderate to vigorous intensity aerobic exercise 3 times per week as recommended by the SCI-specific guidelines [27, 29]) as compared to control group have better CMD risk factors profile (glucose homeostasis, blood lipids, oxidative stress and inflammation markers, atherosclerosis and vascular function, resting heart rate and blood pressure, cardiorespiratory fitness and cardiac function and structure); (2) Whether (and which) tailored exercise prescriptions (i.e., which type, intensity, frequency and duration) could improve CMD risk profile in SCI individuals? and (3) Which SCI-specific or personal factors may affect the association between physical activity and CMD risk factors?

## Methods

### Data sources and search strategy

This review was conducted in accordance with recently published guideline how to perform systematic reviews and meta-analysis in medical research [33]. The findings were reported following the Preferred Reporting Items for Systematic Reviews and Meta-Analyses (PRISMA) [34] guidelines. Five electronic databases were systematically searched by an experienced information specialist (BM) without date and language restrictions. The EMBASE.com, MEDLINE (Ovid), Cochrane CENTRAL and Web of Science Core Collection from inception until 16th April 2021 (date last searched) and additionally the first 200 results were downloaded from the Google Scholar search engine. The computer-based search strategy combined the terms related to SCI, physical activity and exercise, CMD factors and cardiac structure. Details on the search strategies for all databases are provided in the Online Appendix I. In addition, in order to identify further eligible studies, we manually searched the reference lists of relevant systematic reviews, the reference lists of articles included in current review and the studies citing included articles (via Google Scholar).

### Study selection, eligibility criteria and data extraction

Detailed inclusion and exclusion criteria can be found in the review protocol registered at PROSPERO (ID No. CRD42020164458). In brief, observational studies and clinical trials were eligible for inclusion if they: (i) were carried out in adult individuals with SCI; (ii) provided information on association between exercise intervention or self-reported physical activity in SCI and CVD risk factors (glucose homeostasis, blood lipids, oxidative stress and inflammation markers, atherosclerosis and vascular function, resting heart rate and blood pressure, cardiorespiratory fitness and cardiac function and structure) and (iii) provided comparison between exercise intervention and control group or in case of observational studies, physically active individuals with SCI were compared with control group (that could refer either to inactive, sedentary or individuals with considerably lower physical activity level). Studies comparing SCI population with able-bodied individuals, studies providing only pre-post exercise comparisons without control group and interventional studies with duration less than 2 weeks, were not included in the current systematic review. Considering that functional electrical stimulation (FES) may cause different physiological response to as compared to exercise alone [35], to be able to provide an overview of association between exercise alone and on interventions that a person with SCI can engage without professional medical assistance we excluded studies involving FES. In addition, we excluded animal and in-vitro studies, letters to the editor, reviews, commentaries and conference abstracts.

Based on these criteria, titles, abstracts and full-texts were independently evaluated by two reviewers. Any disagreement was settled by reaching a consensus or by consulting a third reviewer. Relevant information was subsequently extracted from full text versions of the selected articles using a previously defined data extraction form. To ensure the accuracy of data extraction, two authors independently extracted the data from the identified articles.

### Quality of evidence assessment

Two reviewers assessed the methodological quality of included studies independently (OAI and SS). The quality of included randomized clinical trials (RCTs) and non-randomized trials was evaluated using the Risk of Bias tool for randomized trials (Rob2.0) and Risk Of Bias In Non-randomized Studies—of Interventions (ROBINS) respectively for methodological quality assessment [36, 37]. Cross-sectional studies were evaluated using the Newcastle–Ottawa Scale (NOS) [38]. Using pre-specified criteria studies were classified as high, moderate or low quality. Detailed information on the assessment of study quality and risk of bias is provided in online supplement. Furthermore, we applied the Grading of Recommendations Assessment, Development and Evaluation (GRADE) approach to score the quality of evidence included in current review [39]. The GRADE approach judges the quality of evidence on two key concepts: the magnitude of effect estimates and quality of evidence (taking into consideration risk of bias, study design, consistency and directness of findings). The evidence is graded: high, moderate, low or very low. Detailed explanation on how the GRADE approach was applied can be found in online supplement.

### Selection and definition of comparison groups in observational studies

Physical activity and inactivity may be defined differently depending of the context of the research. The term inactive is often use to refer to people who do not meet specified physical activity guidelines (people who are performing insufficient amounts of moderate- and vigorous-intensity activity (MVPA)) [40]. However, in studies included in current review we noticed that the terms sedentary and inactive are used interchangeably although the sedentary refers to engagement in a large amount of sedentary behaviour (or it is defined as any waking behaviour characterized by an energy expenditure ≤ 1.5 METs). Thus, in Supplemental Table 1, we provide details on how physical activity was evaluated across observational studies included in the meta-analysis. In the current review, the physically active group was defined either as “meeting or exceeding physical activity recommendations” referring to either: (i) an exercise prescription that was linked with better cardiometabolic fitness or CMD health outcomes as reported by previous SCI-specific or American Heart Association (AHA) guidelines (e.g., moderate to vigorous physical activity of 20–30 min at least twice per week or at least 150 min/week of moderate leisure time physical activity or vigorous activity ≥ 60 min/week [27, 29, 41]); OR to (ii) para-athletes (individuals with SCI engaged in regular training sessions or as professionally trained individuals participating in national and international competitions). Defining “physical inactivity” or “sedentary behaviour” among wheelchair users and those with low mobility is challenging considering that prolonged sitting may be unavoidable [42]. Indeed, the “physically inactive” group definition across studies included in the current review varied considerably. In general, this group was defined as either “not meeting recommendations” or rarely more specific classification was provided (e.g., SCI individuals engaged in no sports, or recreational physical activity, or labour that required physical effort). In all cases, the control group was engaged in substantially lower amount of physical activity as compared to physically active group. The exceptions were four studies: one provided no definition of comparison group [43], one classified as inactive individuals who performed less than 180 min/week of 3 MET tasks [44], one defined as "inactive" individuals who exercised less than 30 min per day [31] and one defined as sedentary individuals in the ‘lowest activity level’ using the questionnaire [45]. Considering that in our leave-one-by-one sensitivity analysis removal of these studies did not affect the overall effect estimates we decided to keep these studies in the main analysis. Considering variations in definitions of physical inactivity/sedentary behaviour (that often referred to physical inactivity rather than true sedentary behaviour) across the studies, in further text, we will refer to these sedentary or inactive individuals as “*control group*”.

### Data synthesis and analysis

In RCTs, the intervention effect was defined as the pre-post differences (mean difference of the differences within groups) in outcomes between exercise intervention and control group at the end of the trial. All outcomes were continuous; thus, the mean differences [intervention minus control] of the intervention effects in CVD risk factors were presented as summary outcome measures. Random-effect models were used to obtain estimates of weighted mean differences (WMDs) and 95% confidence intervals (CIs). For the meta-analysis of cross-sectional studies, we computed for pooled means and standard deviation, and WMD based on the extracted measurement from each study. We pooled mean difference by weight using the random effects model developed by DerSimonian and Laird in 1986 [46]. Besides providing the overall pooled estimates (physically active group vs. control croup), we also provided the pooled estimates: (i) comparing para-athletes and control group and (ii) physically active individuals who were not characterized as professional para-athletes (but were engaged in habitual/regular physical activity and met physical activity recommendations) vs. control separately. The studies that could not be quantitatively pooled (e.g., due to missing information) were descriptively summarized.

Data expressed in International System of Units (SI-units), were converted in accordance to the standard conversion tables of the US National Institute of Standards and Measures to conventional units. For studies where median and ranges (IQR, or minimum–maximum values) were reported, we converted the data into mean and standard deviation to be able to pool the effect estimates across studies (WMD) [47]. To resolve cases of multiple publications based on the same study sample, the most recent information or the publication with the most relevant outcome were extracted. In case of uncertainty, the corresponding author was supposed to be contacted for clarifications in case that an article was published within the past 10 years (none of the studies met this criterion, thus no corresponding authors were contacted for this purpose). We assessed heterogeneity using the Cochrane χ^2^ statistic and the *I*^2^ statistic according to the method described by Higgins JP et al. and were classified from low to high—Low (*I*^2^ ≤ 25%), moderate (25% < *I*^2^ < 75%), or high (*I*^2^ ≥ 75%) [48].

If eight or more studies were included in the meta-analysis [49], they were evaluated using a random effects meta-regression. Study characteristics such as geographical location of the study, median number of participants, and study quality and patient characteristics consisting of median age, sex distribution, hours of exercise per week in physically active group; and injury characteristics (level, duration and completeness of injury) were selected as characteristics for assessment of heterogeneity. A leave one out analysis was performed by iteratively estimating the WMD by removing one study at a time in order to assess the impact of each study in the analyses. We evaluated asymmetry using Egger’s test and publication bias was assessed with a funnel plot. All tests carried out were two tailed taking the *p* value < 0.05 as significant. All statistical analyses were conducted with STATA, Release 16 (Stata Corp, College Station, TX, USA).

## Results

### Literature search and study characteristics

**Fig. 1:**
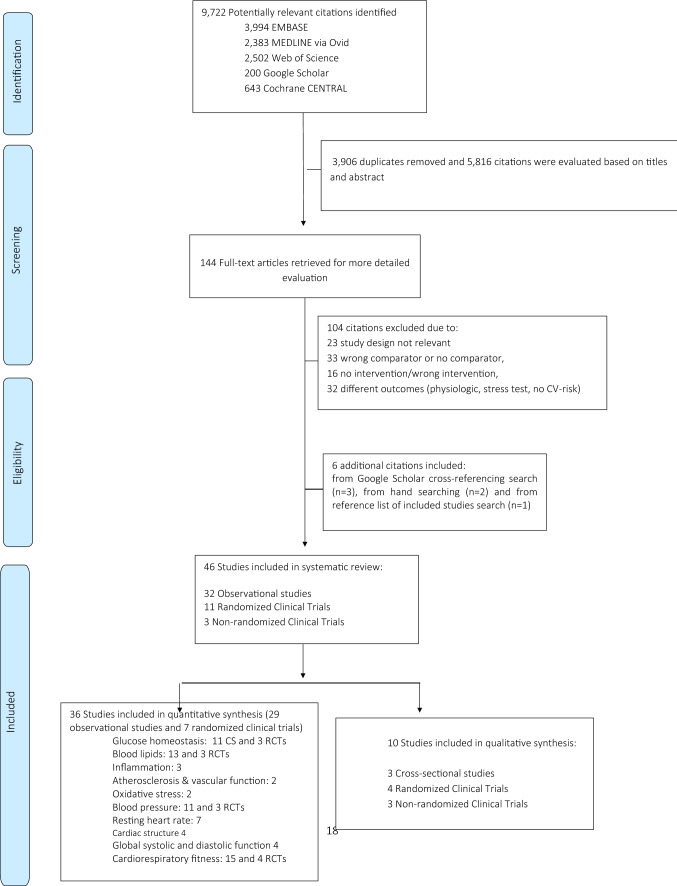
Flow chart of studies included in the current review

Of 5, 816 unique citations evaluated by three reviewers based on the search strategy, 144 relevant full-text articles were retrieved and 46 studies (32 cross-sectional studies, 11 RCTs and 3 non-randomized trial) were included in the current systematic review (Fig. 1). Two longitudinal studies reported on association of physical activity and physical capacity, however, these studies did not meet inclusion criteria of current review (i.e., did not provide control group and did not report health outcomes of interest) [50, 51]. In following sections, we focus on results of our meta-analysis; while detailed information on four RCT, three non-randomized clinical trials and three cross-sectional studies, which were not included in the meta-analysis (e.g., due to missing information on effect estimates that were necessary for quantitative synthesis or because including individuals in the subacute phase of the injury) can be found in Supplemental Tables 2, 3, and 4.

Among the eleven RCTs included in the review, we were able to meta-analyse data from seven RCTs comprising 173 individuals with SCI (98 in intervention group and 75 in control group). Four RCTs included individuals without known CMDs, while three did not provide clear information. Age ranged from 33.1 years to 46.8 years and percentage of male population varied from 64.7 % to 91.3% (one study did not specify sex). Study duration ranged from 6 weeks (3 RCTs) to 36 weeks (one RCT) and the trial's interventions included arm-crank ergometer, indoor hand-bike and different levels of aerobic and resistance and circuit trainings on average from 40 to 240 min/week. Control group was more homogenous (e.g., usual activities, standard care or lifestyle maintenance). Details are provided in Table 1 and Supplemental Table 3 and 5. Non-randomized clinical trials were not included in the meta-analysis and were quantitatively summarized in Supplemental Table 4.

**Table 1:** Characteristics of clinical trials included in current systematic review

Lead author, publication date and country	Sample size (N) and percentage of male population	Duration (weeks)	Intervention characteristics		SCI duration (years)	SCI injury type	Mean age (years)	Mean BMI (kg/m^2^)	Health status	Overall risk of bias	The main findings
			Intervention	Control							
Akkurt et al., 2017^1^ [73]; Turkey	33 (29, 88%)	12	General rehabilitation exercises and aerobic exercise with the arm ergometer for 2 sessions/day 5 days/week	Only general rehabilitation exercises for 2 sessions/day 5 days/week	3.43 ± 3.14	Traumatic, motor complete and incomplete cervical, thoracic and lumbar SCI	34.7 ± 10.3	23.7 ± 3.8	Otherwise healthy (i.e., pressure sores, Bladder infections, cardiovascular diseases or contraindication for exercise)	High	There were no statistically significant intergroup differences at Weeks 0–6, Weeks 6–12 and Weeks 0–12, both in the intervention group and the control group with regard to metabolic syndrome parameters (TC, TG, HDL, LDL, glucose, waist circumference, SBP, DBP)
Gorgey et al., 2016^2^ [74]; USA	11 (11, 100%)	16	Two exercise interventions (functional electrical stimulation cycling versus arm cycling ergometer), 5 days/week	Two overnight stays/pre-training and were used to control for the effects of aging with SCI	5.5 ± 4	Chronic motor complete SCI (C6–T10; AIS A or B). No information on the type of trauma	38 ± 9	25.7 ± 4.3	Comorbidities were not reported/discussed)	Some concerns	There were no changes in the lipid profile in either the exercise or the control groups following the post-intervention or in the follow-up assessment visits
Hicks et al., 2003 [75]; Canada	34 (NA)	36	Supervised progressive 90–120 min exercise training twice weekly for 9 months. Subjects began each exercise session with a warm-up (wheeling around the indoor track or low-intensity arm ergometry) and gentle upper extremity stretching followed an aerobic training, which involved arm ergometry for 15–30 min, at an intensity of approximately 70% maximum heart rate	Control group was offered a bi-monthly education session (together with the EX group) on topics including exercise physiology for persons with SCI, osteoporosis after SCI, and relaxation techniques	9.4 ± 5.9	Traumatic, motor complete and incomplete cervical to lumbar SCI)	39.3 ± 10.7	NA	Otherwise healthy subjects Individuals with ischemic heart disease, unstable angina, dysrhythmia, or autonomic dysreflexia, recent osteoporotic fracture, and tracheostomy were excluded)	High	There were no differences between groups in resting measures of heart rate, SBP or DBP at baseline, nor were there any changes in these variables in either group over the 9 months. Subjects with tetraplegia had similar resting HRs as those with paraplegia, but significantly lower SBP and DBP pressures; there was no effect of time or group assignment on these measures
Kim et al., 2015 [68]; South Korea	15 (9, 60%)	6	The 60 min of exercise/day, 3 days a week for 6 weeks under the supervision of an exercise trainer consisting of 8 min warm up, 44 min on of hand bike exercise and 8 min cool down	Usual activities	6.5 ± 3.8	Motor complete and incomplete cervical and thoracic SCI. No information on the type of trauma	33.1 ± 5.4	21.4 ± 3.2	Otherwise healthy SCI individuals (e.g., cardiovascular disease, uncontrolled type 2 diabetes and hypertension excluded)	High	Participation in a six-week exercise program significantly decreased BMI (baseline: 22.0 ± 3.7 m/kg^2^ vs. post-intervention: 21.7 ± 3.5 m/kg2), fasting insulin (baseline: 5.4 ± 2.9 µU/ml vs. post-intervention: 3.4 ± 1.5 µU/ml), and HOMA-IR (baseline: 1.0 ± 0.6 vs. post-intervention: 0.6 ± 0.3) levels compared to the control group. HDL-C level (baseline: 42.4 ± 11.5 mg/dl vs. post-intervention: 46.1 ± 12.3 mg/dl) increased significantly after training. No significant changes in glucose, TC, TG, or LDL-C levels were observed in the exercise group. VO_2_ peak (baseline: 16.8 ± 7.2 ml/kg/min vs. post-intervention: 21.2 ± 9.1 ml/kg/min) increased significantly in the exercise group compared to the control group (mean difference vs. control, − 2.9 ml/kg/min)
Kim et al., 2019 [69]; South Korea	17 (12, 65%)	6	Daily exercise program consisted of a 25-min warm-up consisting of 5 min of joint exercises, 15 min of exercise on an arm ergometer, and 5 min of stretching, followed by a 30-min exercise program (resistance, circuit, and aerobic training), and a 5 min of cool down (stretching), but the contents of the 30-min exercise were customized for each individual depending on the comorbidities and other factors	Standard care without exercise	10.53 ± 6.9	Motor complete and incomplete cervical, thoracic, and lumbar SCI. No information on the type of trauma	36.8 ± 6.9	21.9 ± 2.82	Otherwise healthy SCI individuals. Individuals with CVD, uncontrolled type 2 diabetes and hypertension, pressure ulcers, and orthopedic problems were excluded	High	The 6-week exercise program significantly decreased the average fasting insulin (baseline: 7.5 ± 4.7 µU/ml vs. post intervention: 4.5 ± 2.2 µU/ml, *p* < 0.05) and HOMA-IR (baseline: 1.5 ± 1.0 vs. post-intervention: 0.9 ± 0.4, *p* < 0.05) in the exercise group, whereas there was no change in control group (between group difference, mean fasting insulin: − 3.2 µU/ml, *p* = 0.003; mean HOMA-IR: − 0.66, *p* = 0.001). HDL-C has increased in exercise group and decreased in control group during the follow up (pre-post difference was 5.5 mg/dl ± 8.0 in exercise group and − 1.7 mg/dl ± 1.9 in control group, *p* = 0.021). There were no differences in glucose, TC and LDL
Lavado et al., 2013 [76]; Brazil	42 (35, 83.3%)	16	Aerobic physical conditioning with moderate intensity of for one hour, twice or three times a week	Control group maintained their daily life activities	4.4 ± 1.9	Cervical and thoracic, motor complete and incomplete	36.3 ± 7.6	NA	Comorbidities were not reported/discussed	Some concerns	The increase of oxygen consumption in the intervention group compared to the control group was observed only at the end of the program. In the values before and after the training period of the intervention group significant differences were also observed
Nightingale et al., 2017 [21] and 2018 [77]; UK	21 (15, 71%)	6	Home-based moderate intensity exercise using a portable arm-crank ergometer four times a week. The first exercise session was supervised by an experimenter and extended by 5 min per session throughout the first week (i.e. from 30–45 min). The last stretch of exercise was > 36 h before follow-up laboratory testing	The control group were encouraged to maintain their usual lifestyle	16.29 ± 10.9	Motor incomplete thoracic SCI. No information on the type of trauma	46.8 ± 7.7	NA	Individuals without acute health issues (i.e., pressure sores, urinary tract infections, and cardiovascular contraindications for testing) or musculoskeletal complaints, and not taking antihyperglycemic medication	High	Compared with controls, intervention group significantly decreased serum fasting insulin (Δ, 3.1 ± 10.7 pmol/l for control and − 12.7 ± 18.7 pmol/l for intervention) and homeostasis model assessment of insulin resistance (HOMA2-IR; Δ, 0.06 ± 0.20 for control and − 0.23 ± 0.36 for intervention). Adipose tissue metabolism, composite insulin sensitivity index (C-ISI Matsuda), and other cardiovascular disease risk biomarkers were not different between groups. The exercise group also increased the VO_2_ peak (Δ, 3.4 ml/kg min)
Ordonez et al., 2013 [55]^3^; Spain	17 (17, 100%)	12	The 3 sessions/week, consisting of warming-up [10–15 min] followed by arm-crank (20–30 min [increasing 2 min and 30 s every 3 weeks]) at moderate work intensity of 50–65% of the heart rate reserve (Starting at 50% and increasing 5% every 3 weeks) and by a cooling down period [5–10 min]	Individuals matched on age, sex, and injury level who did not take part in any training program	4.6 ± 0.29	Traumatic, motor complete SCI below the fifth thoracic level (T5)	29.9 ± 2.6	27.7 ± 4.0	Healthy (individuals with smoking habits and alcohol consumers and individuals receiving medication and/or antioxidantconsumption that may interfere with the redox homeostasis were excluded)	Low	Both total antioxidant status (0.64 ± 0.2 mmol/l vs. 0.88 ± 0.1 mmol/l) and erythrocyte GPX activity (23.6 ± 2.4U/g hemoglobin vs. 27.8 ± 2.2U/g hemoglobin) were significantly increased at the end of the training program. Lipid peroxidation, expressed as plasmatic levels of malondialdehyde, was significantly reduced (0.48 ± 0.13 mmol/l vs. 0.35 ± 0.11 mmol/l). Similarly, protein oxidation, expressed as plasmatic carbonyl group level, was decreased after exercise (1.92 ± 0.3 nmol/mg protein vs. 1.33 ± 0.2 nmol/mg). In the control group, no significant changes in any of the tested parameters were found
Pelletier et al., 2015^4^ [78]; Canada	23 (21,91.3%)	16	Training involved ≥ 20 min of moderate-vigorous aerobic exercise (rating of perceived exertion 3e6 on 10-point scale) and 3–10 repetitions of upper-body strengthening exercises (50%-70% 1 repetition maximum) 2 times per week	Control group maintained existing physical activity levels with no guidance on training intensity	12.0 ± 10.0	Cervical, thoracic, motor complete and incomplete	40.4 ± 11.6	NA	Comorbidities were not reported/discussed	Some concerns	There was a significant increase in peak aerobic capacity (relative VO_2_ peak: 17.2%, absolute VO_2_ peak: 9.9%) and submaximal power output (26.3%) in the control group only
Rosety-Rodriguez et al., 2014 [56]; Spain	17 (17, 100%)	12	Arm cranking exercise program of 3 sessions/week consisting of warm-up (10–15 min), arm crank (20–30 min; increasing 2 min and 30 s every 3 weeks) at a moderate work intensity of 50–65% of heart rate reserve (starting at 50% and increasing 5% every 3 weeks), plus cool-down (5–10 min)	The control participants completed baseline assessments but did not take part in the training program	4.6 ± 0.29	Traumatic, complete SCI at or below T5	29.9 ± 3.7	27.7 ± 4.2	Otherwise healthy (individuals with pressure ulcers and/or coexisting infections, smoking/alcohol intake and receiving medication that may interfere with metabolism, participation in a training program in the 6 months prior to participation in the trial were excluded)	Low	When compared with baseline, plasma levels of leptin, TNF-a, and IL-6 were significantly decreased in the intervention group. In contrast, no significant changes were found in plasma concentrations of adiponectin and plasminogen activator inhibitor-1 (PAI-1)
Totosy de Zepetnek et al. [54]; Canada	23 (21, 91%)	16	The training involved ≥ 20 min of moderate-vigorous aerobic exercise (rating of perceived exertion 3e6 on 10-point scale) and 3–10 repetitions of upper-body strengthening exercises (50%-70% 1 repetition maximum) 2 times per week	Control group maintained existing physical activity levels with no guidance on training intensity	12.0 ± 9.9	Motor complete and incomplete cervical and thoracic SCI. No information on the type of trauma	41.4 ± 11.6	26.5 ± 5.1	Individuals with any progressive loss of neurologic function within the previous 6 months were excluded	High	When implemented as part of a supervised training program, the physical activity guidelines for adults with SCI has a positive influence on some aspects of body composition and carotid vascular health. Despite these benefits, 16 weeks of adherence to the physical activity guidelines did not elicit changes in other CVD risk factors. There was a significant increase in peak aerobic capacity

BMI: body mass index; CVD: cardiovascular disease; DBP: diastolic blood pressure; HDL: High density lipoprotein; HOMA-IR: Homeostatic model assessment for insulin resistance; IL-6: interleukin 6; LDL: Low density lipoprotein; SBP: systolic blood pressure; TC: total cholesterol; TNF-a: tumor necrosis factor alpha

Among 11 RCTs, 7 contributed to meta-analyses, reasons for exclusion are provided below:

^1^Control group received a general rehabilitation exercises, and the population included individuals in subacute injury phase, thus it was not included in meta-analysis (all other trials included subjects in chronic phase of the injury)

^2^Used two types of exercise and did not disaggregate data for functional electrical stimulation (FES), thus it was not included in meta-analysis (FES was exclusion criteria)

^3^Overlapping population with Rosety-Rodriguez et al., 2014, none of trial included in meta-analysis as there were no additional trials to report on inflammation/oxidative stress parameters

^4^Partially overlapping population with study by Totosy de Zepetnek et al. [54]

Information from twenty-nine cross-sectional observational studies comprising 5527 individuals with SCI (2398 physically active and 3129 control SCI individuals) contributed to meta-analysis of habitual/regular physical activity and CMD risk factors. Supplemental Table 2 and 6 summarize the most important characteristics of observational studies included in meta-analysis. All studies included in the meta-analysis were carried out in individuals with chronic SCI (median SCI duration was 10.65 years (IQR 5.8 years; 19.2 years), with exception of a single study that recruited a control group within the rehabilitation centre [52]. Eleven studies (37.9%) were conducted in para-athletes and 18 in individuals engaged in habitual/regular physical activity without professional component. Median engagement in physical activity was 10.8 h/week (Q1:3.75 h/week, Q3:12.2 h/week), based on information provided by 14 studies only (among these 8 studies included professional para-athletes, 5 reported on regular physical activity and one focused on leisure time physical activities). Twenty-three studies were conducted in male population (79%) while only six studies included females. The majority of studies (n = 17, 58.6%) were of small sample size (n ≤ 30). Nine studies were conducted in Europe, nine studies in North America, six in South America, two in Australia and one in the Middle-East and in Asia respectively. Solely one study included individuals with CMD [53], 35.4% of studies excluded individuals with known CVDs or diabetes, while the majority (62.1%) did not provide this information. When considering the mean biomarker levels in active and control groups, the mean values of the majority of study participants fell within normal reference ranges. Details are provided in Supplemental Table 7.

### Glucose homeostasis

**Fig. 2:**
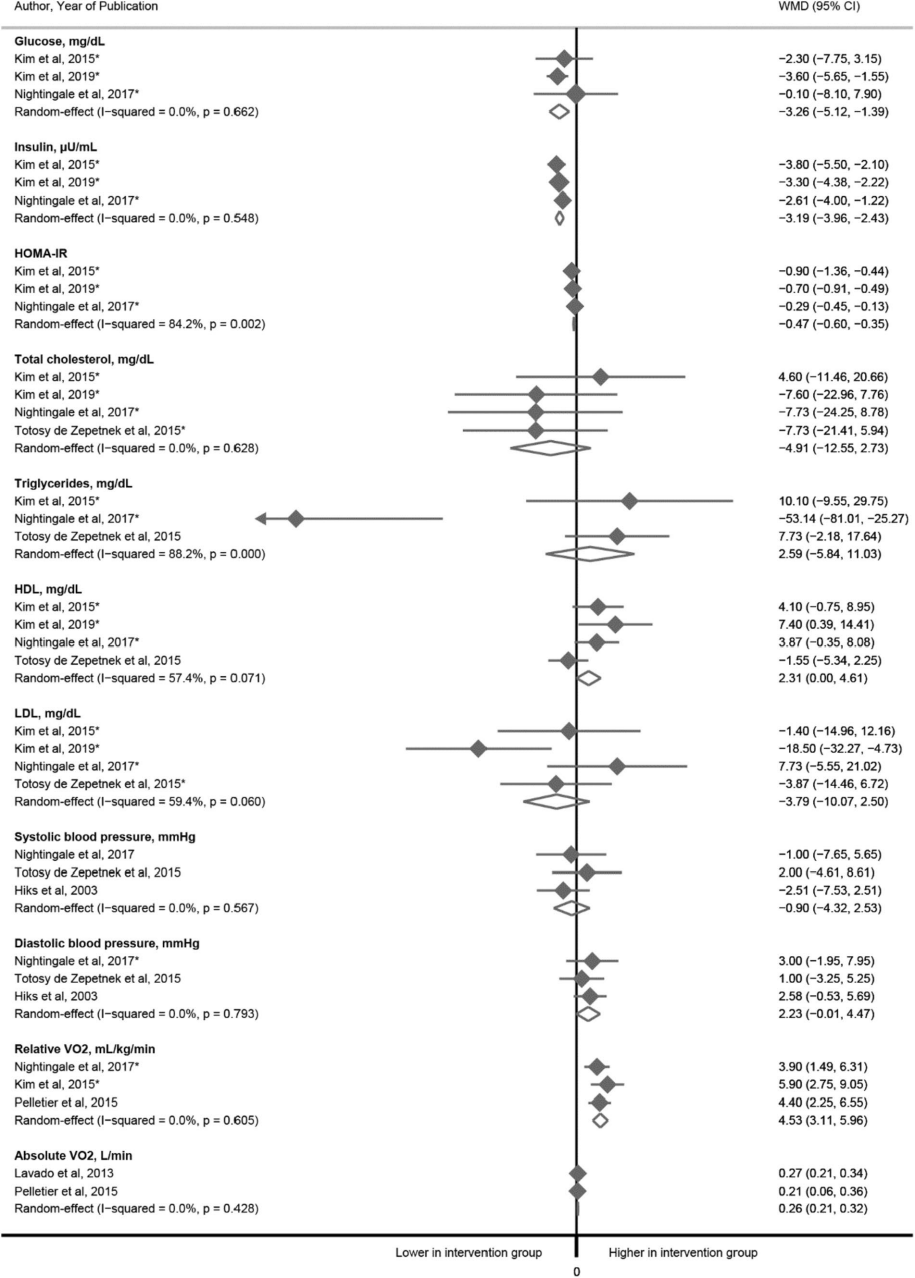
The associations between exercise and glucose homeostasis parameters, blood lipids and blood pressure: a meta-analysis of randomized clinical trials. *Indicates that individuals with cardiometabolic diseases were excluded from the trial (otherwise information was not provided in original articles)

In the meta-analysis of three RCTs (n = 53), a 6-week exercise intervention (90–180 min/week moderate to vigorous aerobic exercise) as compared to control group (usual care/activities) was associated with: (i) a decrease in fasting glucose [WMD was − 3.26 mg/dl (95% CI − 5.12 to − 1.39)], (ii) insulin [WMD was − 3.19 μU/ml (95% CI − 3.96 to − 2.43)] and (iii) Homeostatic Model Assessment of Insulin Resistance (HOMA-IR) [WMD − 0.47 (95% CI − 0.60 to − 0.35], Fig. 2. All three trials included SCI individuals without cardiometabolic complications.

In line with the meta-analysis of RCTs, meta-analysis of cross-sectional studies showed significantly lower glucose and insulin levels in physically active vs. control SCI groups [WMD were − 3.25 mg/dl (95% CI − 5.36, − 1.14) and − 2.12 μU/ml (95% CI − 4.21 to − 0.03) respectively]. In contrast to what was reported in clinical trials, the mean HOMA-IR between the two groups was not different. When stratifying the analyses based on level of the physical activity, glucose levels were significantly lower in para-athletes in comparison with control individuals, while statistical significance diminished when comparing individuals engaged in regular physically activity (without professional component) and control SCI group (Table 2).

**Table 2:** Meta-analysis of cross-sectional studies

Outcome	Number of estimates	Number of physically active SCI individuals	Number of SCI individuals in control group	Mean difference (95% CI)	*I*^2^ for heterogeneity	Number of estimates	Mean difference (95% CI) (Para-athletes vs. control group)	*I*^2^ for heterogeneity	Number of estimates	Mean difference (95% CI) (Para-athletes excluded)	*I*^2^ for heterogeneity
*Glucose homeostasis*											
Glucose, mg/dl	10	166	286	− 3.25 (− 5.36, − 1.14)*	0%	3	− 3.43 (− 6.05, − 0.81)*	0%	7	− 2.91 (− 6.48, 0.66)	0%
HOMA-IR	6	70	56	− 0.22 (− 0.70, 0.26)	45.1%	0	–	–	6	− 0.22 (− 0.70, 0.26)	45.1%
Insulin, μU/ml	7	86	132	− 2.12 (− 4.21, − 0.03)*	37.5%	1	–	–	6	− 2.41 (− 4.63, − 0.20)	42.8%
*Blood lipids*											
Total cholesterol, mg/dl	10	129	312	− 6.72 (− 13.09, − 0.34)*	34.7%	4	− 11.8 (− 18.9, − 4.67)*^1^	12.2%	6	0.93 (− 4.38, 6.23)	0%
HDL, mg/dl	12	181	358	3.86 (0.66, 7.05)*	79.3%*	6	4.87 (1.10, 8.65)*	78.9%*	6	2.44 (− 2.98,7.86)	72.1%*
LDL, mg/dl	9	141	214	− 5.13 (− 12.2,1.91)	28.8%	3	− 9.87 (− 19.1, − 0.65)*	9.5%	6	− 0.33 (− 7.64, 6.98)	0%
Triglycerides, mg/dl	10	129	312	− 3.21 (− 9.44, 3.03)	18.5%	4	− 5.55 (− 11.8, 0.67)	0%	6	− 3.28 (− 17.2,10.7)	37.5%
*Oxidative stress and inflammation*											
Catalase, UgHb^−1^	3	37	28	0.07 (0.03, 0.11)*	0%	1	–	–	2	1.86 (− 4.14, 7.87)	0%
hsCRP, mg/dl	4	53	44	− 0.03 (− 0.10, 0.04)	56.5%	1	–	–	3	− 0.02 (− 0.06, 0.02)	11.2%
*Atherosclerosis and vascular function*											
Carotid IMT, mm	2	80	66	− 0.09 (− 0.16, − 0.02)	68.7%	1	–	–	1	–	–
*Blood pressure and heart rate*											
Systolic blood pressure, mmHg	9	212	268	− 2.31 (− 6.68, 2.06)	36.8%	4	1.21 (− 3.63, 6.05)	0%	5	− 5.08 (− 11.80, 1.64)	44.9%
Diastolic blood pressure, mmHg	8	157	218	− 1.99 (− 5.47,1.50)	37.9%	4	0.93 (− 2.90, 4.75)	0%	4	− 4.92 (− 9.60, − 0.24)*	29.6%
Resting Heart rate, bpm	7	122	103	− 6.93 (− 11.22, − 2.65)*	46.7%	4	− 7.59 (− 11.12, − 4.05)*	0.0%	3	− 3.58 (− 17.99,10.82)	81.3%*
*Cardiac structure*											
Aortic root diameter, mm	3	37	38	1.00 (− 0.65, 2.65)	0%	2	1.01 (− 0.65, 2.68)	0%	1	–	–
LV end diastolic diameter, mm	3	39	36	3.24 (1.06, 5.43)*	23.2%	2	2.89 (0.15, 5.62)*	45.9%	1	–	–
Posterior wall thickness, mm	4	47	45	0.02 (− 0.71, 0.75)	76.6%*	2	− 0.04 (− 0.42,0.35)	19.4%	2	0.09 (− 2.46, 2.64)	91.4%*
Septal wall thickness, mm	5	72	55	− 0.13 (− 0.95, 0.69)	82.4%*	3	− 0.49 (− 1.06, 0.08)	57.4%	2	0.49 (− 1.76, 2.74)	88.4%*
LV mass index, g/m^2^	4	47	45	6.57 (− 8.42, 21.56)	86.6%*	2	7.58 (− 0.07,15.2)	0%	2	6.97 (− 30.5, 44.5)	94.2%*
End diastolic volume, ml	2	18	16	− 2.41 (− 14.44, 9.61)	0%	0	–	–	2	− 2.41 (− 14.4, 9.61)	0%
*Global systolic and diastolic function*											
Stroke volume, ml	4	66	58	9.37 (3.07, 15.66)*	46.2%	3	11.35 (4.04,18.67)*	43.9%	1	–	–
Cardiac output, Q, L/min	3	37	38	− 0.04 (− 0.62, 0.53)	0%	2	0.04 (− 0.62, 0.71)	0%	1	–	–
Ejection fraction, %	5	76	65	− 0.85 (− 2.66, 0.97)	0%	3	− 1.14 (− 3.12, 0.83)	0%	2	0.77 (− 3.86, 5.40)	0%
E/A ratio	4	47	45	0.18 (− 0.07, 0.44)	44.9%	2	0.10 (− 0.13, 0.33)	0%	2	0.24 (− 0.41, 0.89)	70.8%
Isovolumetric relaxation time, ms	2	18	16	− 11.70 (− 19.50, − 3.89)*	0%	0	–	–	2	− 11.70 (− 19.50, − 3.89)*	0%
*Cardiorespiratory fitness*											
Relative VO_2_, ml/kg/min	10	133	102	8.52 (5.52, 11.52) *	85.5%	2	12.25 (7.78, 16.71) *	56.7%	8	7.48 (4.10, 10.86)*	84.7%
Absolute VO_2_, L/min	4	42	42	0.81 (0.46, 1.15)*	85.3%	–	–	–	4	0.81 (0.46, 1.15)*	85.3%
Peak workload, W	5	72	61	53.23 (36.66, 69.80)*	78.7%	1	–	–	4	58.09 (36.40, 79.79)*	78.9%
Peak Heart Rate, bpm	6	84	70	8.49 (0.06, 16.91) *	77.9%	1	–	–	5	8.49 (0.06, 16.91)*	59.2%

^*^Indicates statistically significant results

^1^*p value* from meta-regression was significant

HOMA-IR: Homeostatic Model Assessment for Insulin Resistance; HDL: High-density lipoprotein: LDL: Low density lipoprotein; hsCRP: High-sensitivity C-reactive protein; LV: Left ventricular…;

E/A ratio: Early to late ventricular filling ratio; VO_2_: Maximal oxygen consumption

Physically active group definition was heterogeneous among the studies. The studies included in current review were published between 1975 and 2019. Considering that physical activity recommendations varied over these four decades, we considered that physical activity guidelines recommendations were met or exceeded in the following cases: 1. Individuals in physically active group were engaged in at least 30 min of moderate to vigorous intensity aerobic exercise 3 times per week (90 min/week); conditional SCI-specific recommendation that this physical activity level could improve cardiometabolic health in SCI individuals [29] OR. 2. At least 20 min of moderate to vigorous intensity aerobic exercise twice per week and three sets of strength exercise for each major functioning muscle group at a moderate to vigorous intensity two times per week (linked with improved cardiorespiratory fitness in SCI) [29] OR. 3. Individuals in physically active group engaged in moderate to vigorous physical activity at the frequency of minimum twice per week in duration of 20–30 min OR any sustained physical activity can be of benefit to CVD health in SCI population as long as it meets the requirements for time and intensity [27]. OR. 4. Moderate leisure time physical activity (LTPA) ≥ 150 min/week or vigorous LTPA ≥ 60 min/week, based on ACSM/AHA recommendations (the SCI-specific recommendations were not available) [41] OR. 4. Professional para-athletes were considered to meet physical activity recommendations due to professional component in their engagement in sports. Details can be found in Supplemental Table 1 and “Methods” section

### Blood lipids

In the meta-analysis of four RCTs (n = 74) we did not observe significant differences in total cholesterol and high-density lipoprotein (HDL), nor between low-density lipoprotein (LDL) and triglyceride between exercise and control groups (based on three RCTs and 57 individuals) Fig. 2. Three trials included SCI individuals without cardiometabolic complications, while in study by Totosy de Zepetnek et al. [54] only subjects with progressive loss of neurologic function within 6 months prior to the study were excluded. Trials duration varied from 6 to 16 weeks, intervention comprised from 40 to 180 min/week moderate to vigorous aerobic or resistance exercise, and control group received either standard care or was suggested to maintain regular activity levels.

In contrast to observations from RCTs, meta-analysis of cross-sectional studies showed lower total cholesterol [WMD − 6.72 mg/dl (95% CI − 13.09, − 0.34)] and higher HDL in physically active vs. control SCI group [WMD 3.86 mg/dl (95% CI 0.66, 7.05)]. The results remained stable when comparing para-athletes with control group, while in subgroup analyses comparing physically active vs. less active individuals with SCI the significance was diminished. We observed the difference in LDL levels only between para-athletes and control group [WMD − 9.87 mg/dl (95% CI − 19.1, − 0.65)] while in overall analyses and comparing active and control SCI group we did not observe significant differences in LDL (Table 2). We found no differences in triglyceride levels in neither of the analyses, which was in line with observations from RCTs.

### Oxidative stress and inflammation markers

We identified four studies comparing oxidative stress parameters among physically active and inactive individuals with SCI [7, 43, 44, 55]. In a randomized clinical trial, a 12-week arm-cranking exercise program improved the antioxidant defence system in adults with chronic SCI. Total antioxidant status (0.64 ± 0.2 mmol/l vs. 0.88 ± 0.1 mmol/l) and erythrocyte glutathione peroxidase activity increased (23.6 ± 2.4 U/g hemoglobin vs. 27.8 ± 2.2 U/g hemoglobin) and plasmatic levels of malondialdehyde (0.48 ± 0.13 μmol/l vs. 0.35 ± 0.11 μmol/l) and protein oxidation, expressed as plasmatic carbonyl group level were reduced (1.92 ± 0.3 nmol/mg protein vs. 1.33 ± 0.2 nmol/mg protein) at the end of the training program [55]. Another experimental study explored the effect of exhaustive exercise on systemic oxidative stress and reported a link between exhaustive exercise and transient increase in lipid peroxides and protein carbonyls. Those parameters however decreased to baseline levels 2 h post-exercise cessation [44]. Two cross-sectional studies reported better blood antioxidant defence capacity characterized by higher erythrocyte catalase and glutathione peroxidase, malondialdehyde and changes and overall enzymatic antioxidant potential index in physically active individuals [7, 43]. In current study, we managed to pool effect estimates from 3 cross-sectional studies and found that the catalase was 0.07 UgHb^–1^ (95% CI 0.03, 0.11) lower in physically active in comparison to control group, Table 2.

In addition, six studies explored inflammation markers in physically active vs. control group with SCI [19, 54, 56–59]. In a 12-week clinical trial, arm cranking exercise reduced plasma levels of inflammatory cytokines [TNF-α (23.3 pg/ml vs. 20.6 pg/ml) and IL-6 (6.7 pg/ml vs. 4.1 pg/ml)] in adults with chronic SCI with injury level below fifth thoracic segment [56] while in another 16-week intervention study including individuals with cervical and thoracic SCI changes in inflammatory markers were not observed within the study period [TNF-α, 4.7 pg/ml vs. 4.4 pg/ml; IL-6, 2.5 pg/ml vs. 1.5 pg/ml and plasminogen activator inhibitor-1, 30.4 vs. 42 ng/ml] [54]. When pooling the findings from 4 cross-sectional studies we found no difference in high sensitivity C-reactive protein (hsCRP) between physically active and control groups (Table 2).

### Atherosclerosis and vascular function

In the current review, we found five studies (one trial and four cross-sectional studies) exploring vascular function and atherosclerosis in physically active and SCI individuals [53, 54, 60–62]. Arterial elasticity was better in para-athletes vs. non-athletes with SCI. In particular, aortic pulse wave velocity (PWV) was lower (6.9 ± 1.0 vs. 8.7 ± 2.5 m/s) in elite hand-cyclists with high level of physical exercise training (mean 17 h/week) as compared to sex-matched non-athletes/sedentary individuals with SCI (mean 1 h/week) of comparable age and time since injury [60]. In study by Bell et al. the ankle-brachial index (ABI) was lower in individuals with SCI as compared to controls without the injury, and there was a moderate negative correlation with number of years post-injury and ABI independently of participants’ age. However, the ABI did not differ among physically active and control groups with SCI (0.94 ± 0.11 vs. 0.97 ± 0.10). In addition, they did not report differences in brachial and carotid intima media thickness (CIMT) between the two groups [53]. In contrast, another cross-sectional study reported higher CIMT among para-athletes as compared to sedentary SCI individuals. In the same study they compared the expression of serum microRNAs (miRNAs) among the two groups. The miR-125b-5p, miR-146a-5p, miR-328-3p, miR-191-5p, miR-103a-3p, and miR-30b-5p, which are linked with vascular remodelling, correlated and CIMT and oxidized LDL-cholesterol and showed distinct expression between active and inactive individuals with SCI. Their findings further suggest that circulating miRNA expression in individuals with SCI compared with able-bodied individuals attenuated by regular physical activity [62]. When we pooled the findings from these studies, the CIMT was slightly lower in physically active individuals as compared to controls [WMD was − 0.09 mm (95% CI − 0.16, − 0.02)], Table 2. In contrast to findings from observational studies, in a 16-week randomized clinical trial which evaluated the effects of following the physical activity guidelines in individuals with SCI carotid artery stiffness was improved and no differences in carotid artery structure (i.e., pulse pressure, IMT, wall-to-lumen ratio), regional stiffness or endothelial function in physical activity intervention versus control group [54].

### Resting heart rate and blood pressure

In a meta-analysis of three RCTs including 76 individuals we did not observe significant differences in systolic and diastolic blood pressure between exercise and control groups (Fig. 2). Two trials included SCI individuals without cardiometabolic complications, in one trial cardiometabolic status was not reported. Trials duration varied from 6 to 36 weeks, intervention comprised from 40 to 180 min/week moderate to vigorous aerobic or resistance exercise, and control group was suggested to maintain regular activity levels in two studies, in one, control group received a bi-monthly education session (related to exercise physiology for persons with SCI, osteoporosis after SCI, and relaxation techniques).

Based on meta-analysis of cross-sectional studies resting heart rate was lower in physically active individuals with SCI compared to control group with WMD − 6.93 bpm (95% CI − 11.22, − 2.65), Table 2. Systolic and diastolic blood pressure were not significantly different between the two groups including all studies. However, diastolic blood pressure was lower in subgroup analysis when comparing active versus control SCI groups [WMD was − 4.92 mmHg, (95% CI − 9.60, − 0.24)].

### Cardiac structure and function

We found no RCTs exploring the role of exercise in cardiac structure and function. Based on meta-analysis of cross-sectional studies left ventricular end diastolic diameter was 3.24 mm (95% CI 1.06, 5.43) wider in physically active as compared to control group. There was no statistically significant difference in aortic root diameter, posterior wall thickness, septal wall thickness, left ventricular mass index and end diastolic volume. Further, resting stroke volume was 9.37 ml (95% CI 3.07, 15.66) higher in physically active in comparison to control SCI group. There was no statistical difference in cardiac output, ejection fraction and early to late filling ratio (E/A). Isovolumetric relaxation time was − 11.70 ms (95% CI − 19.50, − 3.89) lower in physically active vs. control group (Table 2). In addition, among included studies, one non-randomized clinical trial indicated that left ventricular indices remained unchanged while there was a tendency for increased cardiac output during the 16 weeks training program undertaken by the intervention group [63].

### Cardiorespiratory fitness

In the meta-analysis of three RCTs (n = 59), relative oxygen uptake relative (VO_2_) was 4.53 ml/kg/min (95% CI 3.11, 5.96) higher in the intervention group as compared to the control group; while among 65 participants and based on only two studies, absolute VO_2_ was 0.26 L/min (95% CI 0.21, 0.32) higher in intervention group (Fig. 2). Results from observational studies were in line with interventional studies; relative and absolute VO_2_ were 8.52 ml/kg/min (95% CI (5.52, 11.52) and 0.81 L/min (95% CI 0.46, 1.15) respectively. The peak workload was 53.23 W (95% CI 36.66, 69.80) higher in the physically active group than in the control group while the peak heart rate was 8.49 bpm (95% CI 0.06, 16.91) higher among the physically active group as compared to the control group (Table 2). Most of the included studies (observational and RCTs) assessed cardiorespiratory fitness using either a computer controlled stationary wheelchair ergometer or an arm crank ergometer and study participants were asked to complete an incremental velocity test until they reached volitional exhaustion at a predefined pace or self-paced rate (Supplemental Table 5).

### Quality and credibility of the evidence, sensitivity analyses, and heterogeneity

For the RCTs, most of the studies (n = 9, 81.1%) were judged as having some concerns for bias in randomization procedure (mostly due to insufficiently explored procedures) and all non-randomized trials were judged to have a high risk of bias mostly due to confounding variables (Supplemental Tables 8 and 9). The majority of cross-sectional studies were judged to be of fair quality using the Newcastle Ottawa scale (n = 30, 93.7%) and the remaining two studies were judged as good quality (Supplemental Table 10). In meta-analysis of RCTs, the heterogeneity was low/moderate for the majority of pooled effect estimates (n = 9, 81.8%) and only analyses of HOMA-IR and triglycerides showed high heterogeneity. We were not able to explore sources of heterogeneity for these two health outcomes considering that only three RCTs contributed to meta-analysis. In meta-analysis of cross-sectional studies, eight pooled effect estimates had high between study heterogeneity with an *I*^2^ estimate exceeding 75% and *p* < 0.05 for the Cochrane χ2 statistic (HDL, relative and absolute VO_2_, peak workload and peak heart rate posterior wall thickness, septal wall thickness, and left ventricular mass index), Table 2. Factors such as age, sex, injury level and whether a person was a professional para-athlete were suggested to be potential sources of heterogeneity across different outcomes (Supplemental Table 11). We used meta-regression to fit a line between study subject characteristics' and the CVD risk factors. With increasing mean age, the difference in systolic and diastolic blood pressure became more apparent between physically active and control groups. With increasing mean age, the difference in total cholesterol and LDL decreased, while WMD in HDL was higher in younger physically active individuals in comparison to control group and this difference became negative with increasing age (meta-regression slopes and significance shown in Supplemental Fig. 1). We observed no linear trend between age and differences in serum triglycerides, yet with increasing SCI duration the difference in LDL became less apparent between the two groups. We observed no linear trends between duration of injury and other CVD risk factors, nor between exercise hours per week and CVD risk markers (meta-regression slopes and significance shown in Supplemental Figs. 2 and 3). With increasing percentage of male population per study, the difference in systolic blood pressure was less apparent between the groups, while the difference in LDL among the groups was higher in studies comprising more males (meta-regression slopes and significance shown in Supplemental Fig. 4). Leave-one-out sensitivity analysis showed that the pooled estimates were not influenced by any specific study for most of the outcomes. In the case of insulin and total cholesterol, where the significant overall estimate might be driven by certain studies; and HOMA-IR and septal wall thickness, where overall non-significant observations could be driven by two studies included in meta-analysis suggesting no consistency (Supplemental Table 13A–L). Due to the small number of RCTs included in the meta-analyses (3–4 RCTs per outcome), we were not able to explore the risk of publication bias. Except for the analysis on peak heart rate, we found no evidence on publication bias in cross-sectional studies. However, the caution is needed as less than 10 studies contributed to analysis for the following outcomes: insulin, HOMA-IR, systolic and diastolic blood pressure, heart rate, ejection fraction, septal wall thickness and peak work load (Supplemental Fig. 5).

After careful consideration of all above mentioned factors, the GRADE approach was used to assess the credibility of evidence presented in current review (Table 3; Supplemental Table 12). The evidence from meta-analysis of RCTs in general SCI population, on whether the engagement in physical activity exercise intervention could improve cardiovascular risk factors as compared to control group showed the following: (i) we report with moderate certainty improved glucose and cardiorespiratory fitness parameters in individuals who were engaged in physical activity interventions (the exercise regimes employed from 40 to 240 min of moderate to vigorous aerobic exercise per week) as compared to the control group; (ii) with low and moderate confidence we report no differences in blood lipids and blood pressure between intervention and control groups, respectively.

**Table 3:** Comparison of meta-analysis findings from cross-sectional studies and RCTs

Outcome	Cross-sectional studies					Randomized clinical trials		
	Overall findings	Median reported exercise hours/week	Para-athletes vs. control group	Physically active vs. control group	Certainty of evidence (the GRADE approach)	Overall findings	Exercise intervention	Certainty of evidence (the GRADE approach)
Glucose	+	8.54 h/week	+	O	Low (C)	+	On average 134 min/week (90–180 min/week) moderate to vigorous aerobic exercise^10^	Moderate (B)
Insulin	+	8.54 h/week	–	+	Low (C)	+	On average 134 min/week (90–180 min/week) moderate to vigorous aerobic exercise^10^	Moderate (B)
HOMA-IR	O	8.54 h/week	–	O	Very low (D)	+	On average 134 min/week (90–180 min/week) moderate to vigorous aerobic exercise^10^	Low (C)
Total cholesterol	+	3 h/week^1^	+	O	Low (C)	O	On average 110.5 min/week (40–180 min/week) moderate to vigorous aerobic or resistance exercise^11^	Low (C)
HDL	+	5.7 h/week^2^	+	O	Low (C)	O	On average 110.5 min/week (40–180 min/week) moderate to vigorous aerobic or resistance exercise^11^	Low (C)
LDL	O	5.7 h/week^3^	+	O	Very low (D)	O	On average 110.5 min/week (40–180 min/week) moderate to vigorous aerobic or resistance exercise^11^	Low (C)
Triglycerides	O	3 h/week^1^	O	O	Low (C)	O	On average 117.3 min/week (40–180 min/week) moderate to vigorous aerobic exercise^11^	Very Low (D)
Systolic blood pressure	O	9.6 h/week^3^	O	O	Low (C)	O	93.3 min/week (40–180 min/week) moderate to vigorous aerobic or resistance exercise^12^	Moderate (B)
Diastolic blood pressure	O	10.6 h/week^4^	O	+	Very low (D)	O	93.3 min/week (40–180 min/week) moderate to vigorous aerobic or resistance exercise^12^	Moderate (B)
Resting Heart rate	+	11.2 h/week^5^	+	O	Low (C)	–	–	–
Catalase	+	3 h/week^6^	–	O	Low (C)	–	–	–
hsCRP	0	10.2 h/week^6^	–	O	Very low (D)	–	–	–
Carotid intima media thickness	+	6.7 h/week^6^	–	–	Low (C)	–	–	–
Aortic root diameter	O	10.6 h/week^6^	O	–	Very low (D)	–	–	–
LV end diastolic diameter	+	10.6 h/week^6^	+	–	Low (C)	–	–	–
Posterior wall thickness	O	10.6 h/week^6^	O	O	Very low (D)	–	–	–
Septal wall thickness	O	10.6 h/week^6^	O	O	Very low (D)	–	–	–
LV mass index	O	10.6 h/week^6^	O	O	Very low (D)	–	–	–
End diastolic volume	O	10.6 h/week^6^	–	0	Very low (D)	–	–	–
Stroke volume	+	10.6 h/week^6^	+	–	Low (C)	–	–	–
Cardiac output	O	10.6 h/week^6^	O	–	Low (C)	–	–	–
Ejection fraction	O	10.6 h/week^6^	O	O	Very low (D)	–	–	–
E/A ratio	O	10.6 h/week^6^	O	O	Very low (D)	–	–	–
Isovolumetric relaxation time	+	9.7 h/week^6^	–	+	Low (C)	–	–	–
Relative VO_2_	+	3 h/week^7^	+	+	Low (C)	+	93.3 min/week (40–180 min/week) moderate to vigorous aerobic or resistance exercise^13^	Moderate (B)
Absolute VO_2_	+	NA^8^	–	+	Very low (D)	+	110 min/week (20–240 min/week) moderate to vigorous aerobic exercise^14^	Moderate (B)
Peak workload	+	NA^9^	–	+	Very low (D)	–	–	–
Peak Heart Rate	+	NA	–	+	Very low (D)	–	–	–

+: Parameter was lower in physically active individuals in comparison to control SCI individuals, in case of HDL and catalase, LV end diastolic diameter and stroke volume, relative and absolute VO_2_, peak workload and peak heart rate, the mean levels were higher in physically active in comparison to control individuals

O: No significant association was observed

–: Meta-analysis was not performed

^1^60% of studies provided information

^2^66.7% of studies provided information

^3^88.9% of studies provided information

^4^87.5% of studies provided information

^5^71.4% of studies provided information

^6^100% of studies provided information

^7^45.4% of studies provided information

^8^None of the studies provided information

^9^Only a single study provided information

^10^Average was calculated based on following information: Glucose, Insulin, HOMA-IR: Nightingale et al., 2017 45 min, 4 times/week, Kim et al., 2019 30 min, 3 times/week and Kim et al., 2015 44 min, 3 times/week

^11^Total cholesterol, HDL, LDL: Nightingale et al., 2017 45 min, 4 times/week, Kim et al., 2019 30 min, 3 times/week, Kim et al., 2015 44 min, 3 times/week, Totosy de Zepetnek et al. 54 20 min, 2 times/week. Triglycerides: Nightingale et al., 2017 45 min, 4 times/week, Kim et al., 2015 44 min, 3 times/week, Totosy de Zepetnek et al., 2015 20 min, 2 times/week

^12^Systolic and diastolic blood pressure: Nightingale et al., 2017 45 min, 4 times/week, Totosy de Zepetnek et al., 2015 20 min, 2 times/week and Hicks et al., 2003 30 min, 2 times/week

^13^Relative VO_2_: Nightingale et al., 2017 45 min, 4 times/week, Kim et al., 2015 44 min, Pelletier et al., 2015, 20 min, 2 times/week

^14^Absolut VO_2_: Pelletier et al., 2015, 20 min, 2 times/week, Lavado et al., 2013 60–120 min, 2 times/week

Certainty of evidence (assessed using the GRADE approach):

High: We are very confident that the true effect lies close to that of the estimate of the effect

Moderate: We are moderately confident in the effect estimate: the true effect is likely to be close to the estimate of the effect, but there is a possibility that it is substantially different

Low: Our confidence in the effect estimate is limited: the true effect may be substantially different from the estimate of the effect

Very Low: We have very little confidence in the effect estimate: the true effect is likely to be substantially different from the estimate of effect

When focusing on meta-analysis of observational studies, our findings suggest the following: (i) we report with low confidence, better glucose homeostasis and lipid profile and higher serum catalase levels (as a proxy of better oxidative status) in physically active individuals as compared to control group; (ii) with very low to low confidence we report beneficial role of physical activity on more favourable cardiorespiratory fitness parameters and systolic and diastolic heart function, as well as the CIMT and (iii) with very low confidence we report no differences in hsCRP between physically active and control groups. These results may be driven by the fact that one third of studies included in meta-analysis comprised professionally trained SCI athletes.

## Discussion

**Fig. 3:**
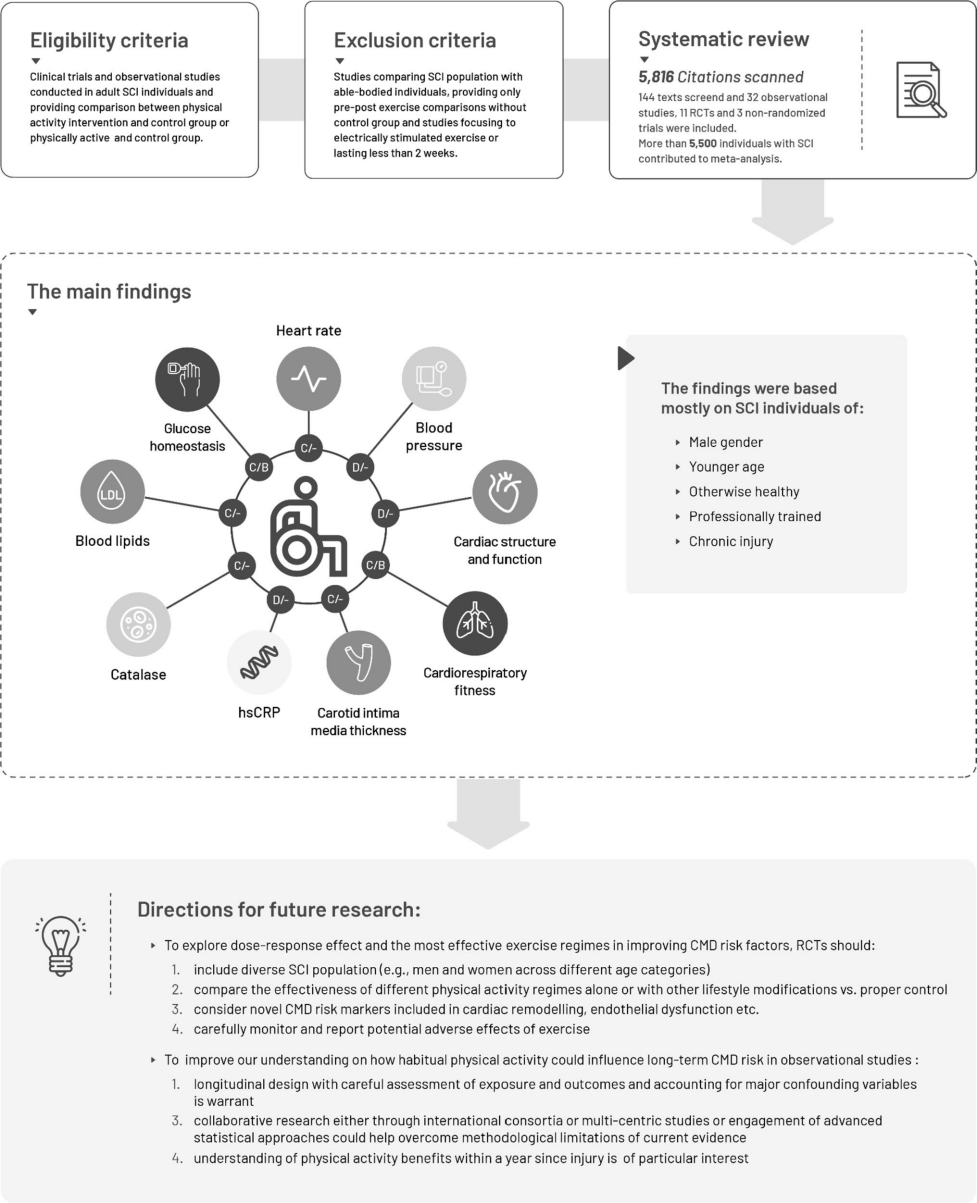
The illustrative summary of the most important findings of the current systematic review. White: No association observed in the meta-analysis. Dark grey: Results were supported by meta-analysis of cross-sectional studies and RCTs (i.e. glucose homeostasis). Grey: Results were supported by meta-analysis of cross-sectional studies only (i.e. blood lipids). Light grey: Results were significant only in cross-sectional studies but not overall (i.e. pooled estimates were significant only in analyses comparing para-athletes with sedentary individuals with SCI). Letters A-D refer to certainty of evidence as assessed using the GRADE approach: A: high certainty, B: moderate certainty; C: low certainty and D: very low certainty; First letter refers to certainty of evidence from cross-sectional studies, second letter refers to certainty of evidence from RCTs (missing letter for RCTs indicates that association was not supported by evidence form RCTs or that meta-analysis was not performed)

Based on limited evidence of moderate certainty coming from RCTs, moderate to vigorous exercise (an average of 93–134 min/week) was associated with improvements in glucose metabolism and cardiorespiratory fitness in SCI individuals free of CMD. Further evidence from RCTs, classified as low to moderate certainty, showed no differences in blood lipids nor blood pressure among exercise and control groups, in contrast to the results of meta-analysis of cross-sectional studies (evidence of low certainty) suggesting that SCI individuals classified as physically active may have better lipid profile and more favourable parameters of systolic and diastolic cardiac function as compared to control group. Limited evidence from cross-sectional studies supported marginally better atherosclerotic profile and less oxidative stress in physically active groups. However, cross-sectional studies are potentially at risk of reverse causality bias, and thus, these should be interpreted with caution. The evidence summary and directions for future research (based on literature gaps identified in current systematic review) are provided in Fig. 3.

### Critical appraisal of current body of evidence

SCI-specific guidelines on physical activity agree that individuals with SCI should avoid inactivity and engage, as much as possible and according to their possibilities, in regular physical activity (e.g., to use manual rather than electrical wheelchairs for everyday transfers) [64–67]. Cardiometabolic health benefits were proposed with as little as 40–150 min of physical activity per week (similarly to recommendations for able-bodied population) [27–30]. In line with previous studies, our findings were consistent in suggesting improvements in glucose metabolism and cardiorespiratory fitness with engagement of SCI individuals in moderate to vigorous exercise or habitual physical activity. However, the evidence of poor methodological quality and with no consistent role of physical activity on lipid profile, oxidative stress, atherosclerotic profile and cardiac function was identified.

The current systematic review underscored the limited quality and size of the SCI evidence on role of physical activity in CMD which is considerably inferior to the evidence gathered among the general population. The current evidence is predominated by observational studies of cross-sectional study design. These studies are at risk of reverse causality bias (e.g., individuals of better health profile including cardiometabolic risk profile may be more prone to engage in active lifestyle) and other biases, thus, the observed associations between habitual physical activity and CMD risk factors may not reflect causality. Another limitation of the cross-sectional studies included in the review was the reporting of mean values (SDs) of CMD biomarkers among comparison groups without providing adjustments for potential confounders/mediators. We explored potential factors that could affect the differences in results across the included studies by performing meta-regression and stratification analysis, and we identified factors such as age, sex, injury level and whether a person was a professional para-athlete as potential sources of heterogeneity across different outcomes. In addition, physically active and control groups were not always clearly defined and the information on type of physical activity (i.e., endurance or strength exercise) across different studies was missing, while the number of exercise hours per week was not always reported which precluded our ability to investigate whether different types and lengths of physical activity can have different influence on cardiometabolic health. This is important factor to take into account in future research considering we found differences in results when studies were stratified by whether they included para-athletes or physically active individuals without professional training engagement. In addition, studies in general did not provide information on lifestyle factors (e.g., diet and or smoking history) nor medication use, and thus we were not able to account for those factors in the meta-analyses, and understand whether the observed associations are a proxy of healthy lifestyle in general. Further, SCI individuals were grouped based on self-reported physical activity, which may introduce a recall bias. In particular, the individuals engaged in healthier lifestyle may be more prone to remember their exercise patterns and report the number of exercise hours per week more accurately. Considering that the benefits of physical activity we observed were more stable in para-athletes, we may speculate that this may either be an indication of dose–response effect or a consequence of healthier life-style engagement among this subgroup of SCI individuals (as compared to general population). Finally, studies in general aimed to recruit healthy SCI individuals, the median age among individuals included in meta-analysis was 33 years and the majority of studies reported mean cardiometabolic biomarker levels within normal reference ranges which may minimize the magnitude of change among blood biomarkers and may have influenced the null findings observed for some outcomes. However, we cannot exclude the possibility that physical activity may simply not influence these outcomes (e.g., blood lipids).

RCTs, although provide more reliable evidence, have limitations that merit to be discussed. First, only a limited number of RCTs could be included in meta-analysis, yet, the overall statistical heterogeneity was low and findings from trials were supported by the pooled effect estimates of cross-sectional studies. However, small number of available trials precluded our ability to explore the role of different exercise regimes on cardiometabolic risk factors. Second, the majority of the trials included individuals without known CMD and relatively young predominantly male population (the mean age ranged from 29.9 to 46.8 years), which may limit generalizability of our findings. Third, at least two [68, 69] out of three trials included in meta-analysis of glucose parameters performed the follow-up assessment within the 48 h of the last training session. Therefore, we cannot speculate whether improvements in glucose levels may be an acute manifestation of exercise or a consequence of long-term exercise prescription.

### Directions for future research

First, besides all studies that contributed to meta-analysis of habitual physical activity were cross-sectional they also included relatively young study populations without major comorbidities which limits generalizability of our findings. Hence, to improve our understanding on how habitual physical activity influence long-term cardiometabolic risk in SCI individuals, well designed, multi-center longitudinal studies (to avoid reverse causality bias) with careful assessment of both, exposure (e.g., using validated physical activity questionnaires and/or diaries or accelerometers) and outcomes and accounting for major confounding variables are a prerequisite. Such factors include age, sex, injury characteristics and injury duration, major lifestyle indices (e.g., diet, smoking and alcohol intake), prophylactic and therapeutic medication use, and parameters of body morphology. Second, while SCI is predominantly a men's condition with around 85% of this population being male, many researchers purposively exclude women from research in order to ensure more homogeneity in study sample [70]. We discuss this issue in depth elsewhere [70], however, a general suggestion to overcome this and other common problems in SCI research (e.g., difficulties to disaggregate results per injury characteristics or injury duration due to small sample size) would be to focus on collaborative research either through international consortia or multi-centric studies or to engage advanced statistical approaches in order to generate reliable evidence [70]. Third, the loss of metabolically active skeletal muscle and limitations in mobility result in decreased basal metabolic rate and resting energy expenditure already within weeks since injury [71, 72]. Understanding physical activity benefits in this early stage would be of particular interest to tackle long-term cardiometabolic risk and develop timely preventive strategies. Fourth, minimal intensity and duration of physical activity/exercise linked with better CMD risk profile remain a matter of debate. Based on our findings it is difficult to speculate whether there is a dose–response effect of physical activity among SCI population or this is solely a consequence of interplay between various aspects of healthy lifestyle observed in professionally trained SCI individuals. This is of particular interest as the SCI population may require increased physical activity engagement (vs. non-SCI) to achieve a given reduction in CMD risk (considering reduced absolute energy expenditure for a given absolute exercise intensity) [32]. To study the dose–response effect, and explore the most effective exercise regimes in improving CVD risk factors, carefully-designed RCT should: (i) Include diverse SCI populations (i.e., men and women across different age categories, individuals in the subacute phase of the injury etc.) rather than to purposively focus on homogenous populations on expenses of generalizability of the findings; (ii) compare the effectiveness of physical activity alone and physical activity together with dietary intervention or other lifestyle modifications vs. proper control (e.g., usual physical activity pattern and usual diet). Considering that lifestyle monotherapies are likely insufficient to modify CMD risk factors in persons with SCI and practical constraints on absolute low energy expenditure in this population which may limit the utility of exercise to impact CMD risk factors that require a minimum calorie cost/deficit for modification [32, 20]. (iii) Consider novel CVD risk markers included in cardiac remodelling, endothelial dysfunction etc. (i.e., apolipoprotein M, matrix metalloproteinases, intercellular cell adhesion molecule-1, etc.) and (iv) Carefully monitor and report potential adverse effects of exercise.

## Conclusions

Evidence of moderate certainty shows that moderate to vigorous exercise may improve glucose metabolism and cardiorespiratory fitness in SCI individuals. Due to lack of rigorous and prospective studies among SCI individuals, uncertainty remains on the impact of habitual physical activity and exercise interventions on other cardiometabolic risk factors. To design effective physical activity recommendations that can improve cardiovascular health in individuals with SCI further methodologically sound prospective observational and interventional studies are warranted.

## Supplementary Information

Below is the link to the electronic supplementary material.

Supplementary file1 (DOCX 2109 kb)

## Data Availability

The datasets generated during and/or analyzed during the current review are available from the corresponding author on reasonable request.
