# Supplementary material for: Physical activity and cardiometabolic risk factors in individuals with spinal cord injury: a systematic review and meta-analysis
Source: Eur J Epidemiol. 2022 Apr 7;37(4):335–65. doi: 10.1007/s10654-022-00859-4 (PMC9187578; doi:10.1007/s10654-022-00859-4)
Supplement: Supplementary file 1 — Supplementary file1 (DOCX 2109 kb) [file 10654_2022_859_MOESM1_ESM.docx]

List of materials provided in online supplement

**Supplemental tables**

**Supplemental table 1**. Physical activity assessment across observational studies included in the current meta-analysis

**Supplemental Table 2**. Characteristics of included cross-sectional studies

**Supplemental table 3**. Study characteristics of clinical trials

**Supplemental table 4**. Descriptive summary of clinical trials investigating the associations between physical exercise and intermediate cardiovascular risk factors in individuals with SCI

**Supplemental table 5.** Cardiorespiratory fitness assessment in observational studies and clinical trials

**Supplemental table 6**. Descriptive characteristics of cross-sectional studies included in the meta-analysis (n=29)

**Supplemental table 7.** Mean values of cardiovascular risk factors, cardiac structure and function and cardiorespiratory fitness in physically active and control group (observational studies

Supplemental Table 8. Risk of bias assessment of the randomized controlled trials (RCT) based on the Cochrane Collaboration’s tool (RoB 2)

**Supplemental Table 9**. Risk of bias assessment of the non-randomized controlled trials based on the Cochrane Collaboration’s tool (ROBINS-I)

**Supplemental table 10.** Risk of bias assessment of observational studies using Ottawa scale (NOS)

**Supplemental Table 11.** Subgroup analyses of individual factors, injury characteristics and study design factors

**Supplemental table 12**. Grading of Recommendations Assessment, Development and Evaluation (GRADE) of all outcomes in observational studies and randomized controlled trials

**Supplemental Table 13 A-**L. Leave one out analyses

**Supplemental Figures**

Supplemental Figure 1. Meta-regression of intermediate cardiovascular outcomes with age as a continuous variable

Supplemental Figure 2. Meta-regression of intermediate cardiovascular outcomes with hours of exercise per week as a continuous variable

Supplemental Figure 3. Meta-regression of intermediate cardiovascular outcomes with duration of injury as a continuous variable

Supplemental Figure 4. Meta-regression of intermediate cardiovascular outcomes with percentage of male participants (sex) as a continuous variable

**Supplemental Figure 5.** Assessment of publication bias (funnel plots and Egger’s test)

**Online Appendix I**: Detailed search strategies used in this review

**Supplemental table 1.** Physical activity assessment across observational studies included in the current meta-analysis

| **Author, year of publication** | **Definition used to classify subjects as active as defined in original studies** | **Physical exercise (hours/week)** | **Definition used to classify subjects as inactive as defined in original studies** | **Physical exercise (hours/week)** | **Tool/questionnaire used to assess physical activity** |
| --- | --- | --- | --- | --- | --- |
| Bell et al, 2011(1) | Active groups met or exceeded 150 min/week of moderate-intensity exercise as recommended by the American College of Sports Medicine/American Heart Association. | NA | Subjects with less than 150 min/week were classified as sedentary SCI. | NA | Questionnaire not specified. |
| Bhambhani et al, 1995(2) | Endurance trained group were regular participants of competitive wheelchair marathon racers with regular training. | NA | Subjects in the untrained group participated in recreational activities once or twice a week only and did not participate in any systematic physical training programs. | NA | Questionnaire not specified |
| Brenes et al, 1986(3) | Highly trained SCI athletes who participated in the 1983 National Wheelchair Athletic  Association (NWAA) Games (USA) and athletes involved in their own training schedules. All athletes were involved in aerobic exercise programs for a minimum of 3 days/week. | NA | Admitted subjects with traumatic SCI to a rehabilitation center were classified as sedentary. No information on rehabilitation exercise program was provided. | NA | Questionnaire not specified. |
| Buchholz et al, 2009(4) | Active groups participated in 25 to 260 min/day of Leisure Time Physical Activity (LTPA). LTPA was defined as activities such as exercising, playing a sport, going for a walk–wheel or engaging in activities of daily living. Most active participants were classified as participants among the top 37%  of the LTPA distribution (≥25 min/day) | 8.54 ± 5.81 | Subjects who did not report any LTPA whatsoever were classified as inactive. | NA | Self-reported LTPA was recorded using the validated Physical Activity Recall Assessment for People with SCI (PARA-SCI) questionnaire. |
| Currie et al, 2015(5) | Elite male athletes with SCI recruited from the Canada Cup International Wheelchair Rugby Tournament and Para-cycling World Championship (Baie-Comeau, Quebec, Canada). | 17 ±3 | Nonathletic males with SCI were recruited  from the community. | 1±1 | Self-reported physical activity levels including the number of hours of exercise per week and the number of years competing (for athletes) was recorded. Questionnaire not specified. |
| Currie et al, 2017(6) | Trained Paralympic wheelchair rugby athletes met or exceeded at least 15 hours/week of exercise training and had at least 3 years of competitive sports participation. | ≥15 | Untrained subjects participating in less than 5 hours/week of physical activity or exercise training. | ≤5 | Questionnaire not specified. |
| Dallmeijer et al^a^, 1997(7) | Active groups were engaged in at least 1.5 to 6 hours/week of regular sports activities for at least 6 months | 3 ± 1.7 | Subjects not participating in regular sports activities were classified as sedentary. | NA | Questionnaire not specified. |
| Dallmeijer et al^b^, 1997(8) | Active group was already involved in at least 1.5 hours/week of quad rugby training before the commencement of the study | NA | Subjects not performing regular quad rugby training were classified as inactive | NA | Questionnaire not specified |
| Davis et al, 1998(9) | Trained provincial or national caliber athletes. Eight active Individuals performed track athletics 4 times/week. Seven athletes performed a variety of sports at least 3 times/week including swimming, basketball and weightlifting | NA | The inactive group was involved in regular leisure time physical activity less than 3 times/ week or participated in recreational activities such as bowling, horseback riding and volley ball | NA | Questionnaire not specified |
| De Rossi et al, 2014(10) | Athletes with SCI had been training and performing sports for an average of 4.2 years at the point of recruitment. | 10.8 ±1.2 | Non-athletes with SCI did not perform  sports, recreational physical activity, or labor-intensive physical effort. | NA | Questionnaire not specified. |
| Dearwater et al, 1986(11) | Highly trained male athletes engaged in competitive training of 135 min/week or 30 to 60 min/day some of whom qualified for the NWAA games (USA). | 2.25 - 7 | Admitted males with SCI at a rehabilitation center were classified as sedentary. | NA | Questionnaire not specified. |
| D'Oliveira et al, 2014(12) | Active groups engaged physical exercise three times per week or more at least 150 min/week for least 3 months. | 13.0 ±7.0 | Subjects not engaging at least 150 min/week of physical exercise were classified as non-physically active. | NA | Trained interviewers collected data on hours/week of exercise and duration of continuous  practice of physical exercise since injury using a structured  questionnaire. Questionnaire not specified. |
| Flank et al, 2014(13) | Active groups engaged in a minimum of 30 mins/day of physical activity | 1.7 ± 3.03 | Inactive individuals were defined as engaging in less than 30 min/day of physical activity. | NA | Physical activity levels were assessed by a Swedish questionnaire, adapted  and tested for this SCI population. The questionnaire was modelled after  2 existing surveys developed for English-speaking persons with disabilities.(14, 15) |
| Hübner-Woźniak et al, 2010(16) | Semiprofessional rugby players and wheelchair rugby players | NA | Inactive individuals were defined as disabled SCI wheelchair users not engaging in sports activities. | NA | Questionnaire not specified. |
| Hübner-Woźniak et al, 2012(17) | Active group included professional wheelchair rugby players engaged in 2–3 h/session training twice a week. | 4-6 | Inactive males with SCI not participating in sports activities were defined as sedentary. | NA | Questionnaire not specified. |
| Huonker et al, 1998(18) | Active group comprised of elite male athletes of various disciplines; all members of the German Paralympic team | NA | Male subjects who were participants at the beginning of a long-term vocational rehabilitation program 2 years after the onset of SCI were classified as sedentary. | NA | Questionnaire not specified. |
| Koury et al, 2013 (19) | Subjects who practiced a minimum of 150min/week (at least 3 days a week) of physical activity for at least 3 months were classified as active. | 12.5±6.7 | Subjects who did not meet a minimum of 150 min/week of physical activity were classified as non-physically active/inactive. | NA | Information on type of physical activity, time spent in total  daily physical activity (min) and time of physical activity practice after injury  (months) was obtained through a structured questionnaire.  Questionnaire not specified. |
| Lovell et al, 2012(20) | Subjects who have been training for over 2 years and regularly participated in national and international road hand cycling competitions were classified as trained. | NA | The untrained SCI men (less than 2 sessions per week) participated in swimming, basketball, tennis and gym sessions. | NA | Data on current level of physical activity, years of hand cycle training, average training  Volume/week (in kilometers), number of training sessions/week and best 20-km time trial completed over  the past year was collected through a questionnaire. Questionnaire not specified. |
| Maggioni et al, 2012(21) | Subjects were classified as a ‘long-trained endurance athlete’ if they had a history of endurance training of 1.5 hours/session at least three times weekly. | 4.5 | Subjects were classified as sedentary if they were categorized in the ‘lowest activity level’ of the questionnaire | NA | Training status/physical activity of the subjects was assessed by the  localized Italian version of the validated International Physical Activity Questionnaire.(22) |
| Paim et al, 2019(23) | Active group comprised competing athletes who were regularly performing wheelchair sports for at least 1 year. | 11.2±6.4 | Subjects who performed no sports, recreational physical activity, or labor that  required physical effort were classified as sedentary. | NA | Questionnaire not specified. |
| Sadowska-Krępa et al, 2015(24) | Subjects who participated in wheelchair rugby from the Polish Wheelchair Rugby League and who met 3hours/week of training were classified as active. | 3 | Definition of inactive not specified. | NA | Questionnaire not specified. |
| Schreiber et al, 2014(25) | Physically active group comprised competing athletes that were regularly different sports activities. | 11.9 ± 1.4 | Subjects that performed no sports, recreational physical activity or labor that required physical effort were classified as sedentary. | NA | Questionnaire not specified. |
| Schreiber et al, 2018(26) | Physically active group included competing athletes that were regularly performing adapted sports. | 11.0±5.9 | Subjects that did not perform labor that required physical effort, recreational physical activity or sports were classified as sedentary. | NA | Questionnaire not specified. |
| Schumacher et al, 2009(27) | Athletes from different disciplines including medal winners at Paralympic games who engaged in endurance training more than 15 h/week on average for more than 3 years were considered trained. | >15 | SCI subjects were considered untrained if they engaged in less than 3 h/week of physical activity in the last year. | <3 | Questionnaire not specified. |
| Ingles et al, 2016(28) | Those who performed more than  180 min per week of MVPA (values greater than 3 metabolic equivalents of task) were included in the active group and | NA | Those who performed less than 180 min per week were included in the non-active group | NA | An accelerometer Actigraph model GT3X (Actigraph, Pensacola, FL, USA) was used to collect the accelerations achieved over 7 days. This device was previously validated for this population |
| Zwiren et al, 1975(29) | Wheelchair-bound athletes who engaged in vigorous training and competed internationally | NA | Wheelchair-bound sedentary who did not undergo any physical training | NA | Questionnaire not specified. |
| Wecht et al. 2006(30) | Individuals who participated in aerobic conditioning more than 3 d/week for at least 30 min per session for the last 6 months were considered endurance trained (wheelchair pushing, arm cycle conditioning, and swimming) Fitness status was confirmed by performing a peak arm cycle ergometry (ACE) exercise test; subjects were determined fit if they achieved ≥ 85% of their predicted peak oxygen consumption (V˙ O2) | NA | Sedentary individuals with paraplegia were defined as not having participated in regular aerobic conditioning for the past year, which was confirmed on the peak ACE test as those not achieving 85% of their predicted V˙ O2 | NA | The same questionnaire was used as in the Framingham health study |
| Tanhoffer et al, 2014(31) | Subjects performed regular and structured exercise at least 3 times per week (minimum of 150 min of exercise weekly), for a minimum of 6 months of continuous training | 3.6 ± 1.2 | Individuals performed structured exercise equal or less than 60 min weekly, they were assigned to sedentary group | 28 ± 31 min/week | A self report diary was completed by participants during the observation period (14 days). Information about physical activity such as type, duration and self-perceived intensity of activity (very light, light, moderate, intense and very intense), as well as date and time of the day were recorded over the 2 weeks DLW protocol. All participants who were engaged in exercise programs performed their physical activities under professional supervision, at gyms or sport associations. To compute the time spent in exercise weekly, only those activities self-reported/assessed in moderate intensity or higher were considered. |
| Lee et al, 2015(32) | Subjects that regularly exercised, at least 3 sessions of one or more sports activities per week lasting a minimum of 1 hour | NA | Subjects that did not regularly exercise | NA | Questionnaire not specified |
| We considered that physical activity guidelines recommendations were met or exceeded in the following cases:   1. Individuals in physically active group were engaged in at least 30 min of moderate to vigorous intensity aerobic exercise 3 times per week (90 min/week), conditional recommendation that this physical activity level could improve cardiometabolic health in SCI individuals. (33) **OR** 2. At least 20 minutes of moderate to vigorous intensity aerobic exercise twice per week and three sets of strength exercise for each major functioning muscle group at a moderate to vigorous intensity two times per week (linked with improved cardiorespiratory fitness in SCI) (33) **OR** 3. Individuals in physically active group engaged in moderate to vigorous physical activity at the frequency of minimum twice per week in duration of 20-30 minutes OR any sustained physical activity can be of benefit to CVD health in SCI population as long as it meets the requirements for time and intensity.(34) **OR** 4. Moderate leisure time physical activity (LTPA) ≥150 min/week or vigorous LTPA ≥60 min/week, based on ACSM/AHA recommendations (the SCI-specific recommendations were not available)(35) **OR** 5. Professional para-athletes were considered to meet physical activity recommendations due to professional component in their engagement in sports | | | | | |

Supplemental Table 2. Characteristics of included cross-sectional studies

| **Author, year of publication** | **Study Design** | **Study Location** | **Population** | **Health**  **Status** | **Medication use** | **No. of individuals** | **Sex, number and % of male** | **SCI lesion duration (mean y±SD )** | **Type of SCI lesion** | **Physical exercise (hours/week)** | **Age**  **(mean y±SD )** | **Outcomes** | **Study Quality** |
| --- | --- | --- | --- | --- | --- | --- | --- | --- | --- | --- | --- | --- | --- |
| Bell et al, 2011(1) | CS | USA | Active SCI men (n=55) and sedentary Spinal cord Injured (SCI) Men (n=50) with paraplegia and tetraplegia | Diabetes (7.2% in active group, 12% in sedentary group) | Antihypertensives/antiarrhythmics (11.43%); Antilipidemic (4.76%); Insulin/glucophages (3.81%) | 105 | 93, 89% | 19.2 ± 8.9 | NA | NA | 40.6±10.9 | Atherosclerosis and vascular function, blood pressure | 7 |
| Bhambhani et al, 1995(2) | CS | Canada | Endurance trained SCI individuals (n=8) and untrained SCI individuals (n=8) | NA | NA | 16 | 16, 100% | NA | Cervical SCI. No information on the type of trauma. | NA | 32.7±7.9 | Oxygen consumption, heart rate | 7 |
| Brenes et al, 1986(3) | CS | USA | Highly trained athletic individuals (n=22) and sedentary individuals(n=66) with SCI | NA | NA | 88 | 73, 83% | NA, Chronic and acute (control group) | Traumatic SCI. No information provided on lesion type | NA | 28.7 ± 9.3 | Blood lipids | 7 |
| Buchholz et al, 2009(4) | CS | Canada | Physically active individuals (n=28) and inactive individuals (n=28) with SCI | NA | NA | 56 | 44, 79% | 14.6±10.1 | Traumatic SCI. No information provided on lesion type | 8.54 ± 5.81 | 41.9±12.2 | Blood glucose, insulin, blood pressure, blood lipids and inflammatory markers | 8 |
| Currie et al, 2015(5) | CS | Canada | Non-athletes (n=8) and athletes (n=13) with chronic, motor-complete, cervical SCI. | NA | NA | 21 | 21, 100% | 16 ± 7.3 | Traumatic, chronic, motor-complete, cervical SCI | 7.1 ± 2.0 | 40.8 ± 8.4 | Blood pressure, peak heart rate, heart function and vascular function | 6 |
| Currie et al, 2017(6) | CS | Canada | Trained individuals with tetraplegia (n=8) vs untrained individuals with tetraplegia (n=9) | All  participants were free from overt disease and no participants reported comorbidities including hypertension, diabetes, and dyslipidemia | No CVD medication use reported | 17 | 17, 100% | 16.9 ± 9 | Traumatic, chronic, cervical, motor-complete. | ≥15 | 35.3±8.1 | Heart structure and function, blood pressure, vascular function | 7 |
| Dallmeijer et al, 1997^a^(7) | CS | Netherlands | Active SCI men (n=11) and sedentary SCI men (n=13) both with tetraplegia with complete and incomplete lesions | Subjects without symptoms of CVD or other medical complications were included in the study | NA | 24 | 24, 100% | 5.8 ± 6.7 | Chronic cervical SCI. No information on the type of trauma. | 3 ± 1.7 | 33.0 ±8.5 | Blood lipids, incident CVD, vascular function, | 6 |
| #Dallmeijer et al, 1997^b^(8) | CS | Netherlands | Active male Quad rugby players (9), inactive persons with tetraplegia (male=7, females=2) | Some subjects suffered from Urinary tract infections and musculoskeletal problems | NA | 18 | 16, 89% | 6.6 ± 8.2 | Motor complete and incomplete cervical SCI (ASIA impairment scale B and D). No information on the type of trauma | NA | 30.6 ±12.7 | Cardiorespiratory fitness, peak oxygen uptake | 6 |
| De Rossi et al, 2014(10) | CS | Brazil | competing tetraplegic athletes that were regularly performing wheelchair rugby (n=15) and competing paraplegic ones that were regularly performing basketball (n=12), handball (n=1), and tennis (n=1) vs. 29 SCI sedentary men | All enrolled subjects were normotensive, nondiabetic, nonsmoker, and normolipemic. | NA | 58 | 58, 100% | 8.4 ± 1.0 | Chronic, cervical and thoracic motor complete | 10.8 ±1.2 | 30.7 ± 6.8 | Heart structure and function, blood pressure, blood lipids | 7 |
| Davis et al, 1988(9) | CS | Canada | Highly active(n=15) vs inactive (n=15) paraplegics | NA | NA | 30 | 30, 100% | 12.6± 5 | Traumatic (n=20) and non-traumatic (n=10) chronic thoracic and lumbar SCI | NA | 27.8 ±4.3 | Heart function, cardiorespiratory fitness | 7 |
| Dearwater et al, 1986(11) | CS | USA | Extremely inactive sedentary individuals with SCI (n=77) and Highly trained active SCI athletes (n=17) | NA | NA | 94 | 94, 100% | NA | NA | 2.25 - 7 | 29.4 ± 10.4 | Blood lipids and blood glucose | 7 |
| D'Oliveira et al, 2014(12) | CS | Brazil | Physically active Individuals (n=14) and inactive (n= 8) with cervical SCI | NA | NA | 22 | 22, 100% | 10.2 ±8.2 | Motor complete and incomplete cervical SCI. No information on the type of trauma. | 13.0 ±7.0 | 31.2 ±9.6 | Blood glucose and Insulin | 6 |
| Flank et al, 2014(13) | CS | Sweden | Physically active individuals with SCI who underwent vigorous exercise (n=27) vs individuals with SCI who underwent moderate exercise (n=107) | NA | NA | 134 | 103, 77% | 18.5 ±12.3 | Traumatic, motor complete and incomplete thoracic and lumbar SCI | 0.9 ± 2.2 | 47.8 ± 13.8 | Blood pressure, blood lipids and blood glucose, | 7 |
| Hubli et al, 2014(36) ¥ | CS | Canada | Athletes (Hand cyclists) with SCI (n=10) and Non-athletes with SCI (n=10) | None of the individuals reported a history of smoking  or CVD, such as coronary artery disease, pulmonary  disease, diabetes mellitus, or metabolic syndrome | NA | 20 | 16, 80% | 18 ± 8 | Traumatic, motor complete chronic cervical and Thoracic SCI | 9 ± 3.2 | 41.5 ±8.9 | Atherosclerosis, blood pressure, heart function and vascular function | 7 |
| Hübner-Woźniak et al, 2010(16) | CS | Poland | Inactive disabled men using wheelchairs (n=10), and Active wheelchair rugby players (n=10). | NA | NA | 20 | 20, 100% | 8.4 ± 4.9 | Cervical SCI. No information on the type of trauma. | NA | 28.7 ± 5.8 | Blood lipids | 6 |
| Hübner-Woźniak et al, 2012(17) | CS | Poland | Sedentary males with tetraplegia (n=10) and wheelchair rugby players with tetraplegia (n=14). | NA | NA | 24 | 24, 100% | NA | Cervical SCI. No information on the type of trauma. | 4-6 | 29 ±5.3 | Oxidative stress, blood antioxidants | 6 |
| Huonker et al, 1998(18) | CS | Germany | Athletes with paraplegia of various disciplines (cross-country sledding, n=12; wheelchair racing, n=10; wheelchair basketball, n=7; all members of the German Paralympic team) vs. sedentary male subjects with paraplegia (n=20) | NA | NA | 49 | 49, 100% | NA | Thoracic, Lumbar, Sacral. Traumatic SCI | NA | 33.0 ± 8.6 | Heart structure and function, vascular function, cardiorespiratory fitness | 6 |
| Ingles et al, 2016 (28) | CS | Spain | Active (n=9) and inactive (n=7) males and females with paraplegia | WIthout symptoms of cardiorespiratory disease | NA | 16 | 14, 87.5% | 14.5 ± 10.9 | Traumatic and non-traumatic, motor complete and incomplete thoracic SCI | >3 | 42.1 ± 11.7 | Oxidative stress, cardiorespiratory fitness | 6 |
| Koury et al, 2013 (19) | CS | Brazil | Physically active (n=13) and non-physically active (n=9) individuals with cervical SCI | NA | NA | 22 | 22, 100% | 11.6±8.7 | Traumatic, motor complete and incomplete cervical SCI | 12.5±6.7 | 33.7±9.9 | Blood glucose and insulin, inflammatory markers | 6 |
| Lovell et al, 2012(20) | CS | Australia | Highly trained handcyclists (n=10) vs untrained SCI men (n=10) | NA | NA | 20 | 20, 100% | NA | Traumatic and non-traumatic, complete and incomplete SCI. Thoracic and Lumbar, Spina bifida (lumbar) | NA | 39.0 ± 7.1 | Heart function, cardiorespiratory fitness | 6 |
| Maggioni et al, 2012(21) | CC | Italy | Aerobically trained paraplegic and tetraplegic patients (n=10) vs untrained paraplegic and tetraplegic patients (n=7) | NA | NA | 17 | 17, 100% | NA | Thoracic, Lumbar lesions. No information on the type of trauma. | 4.5 | 34.2 ± 8.3 | Heart structure and function, vascular function, cardiorespiratory fitness | 7 |
| Nightingale et al, 2017(37) ¥ | CS | United Kingdom | Sedentary individuals with SCI compared to individuals with spinal cord injury performing light intensity or moderate to vigorous physical activity | Recruited SCI individuals were not under any medical care and  were not taking type 2 Diabetes medication. | NA | 33 | 27, 82% | 15 ± 10 | Motor complete and incomplete thoracic and lumbar SCI. No information on the type of trauma. | 2 ± 3.2 | 44 ± 9 | Glucose homeostasis markers, blood lipids, inflammatory markers, cardiorespiratory fitness | 6 |
| Paim et al, 2019(23) | CS | Brazil | Athletes with SCI (n=23) and sedentary individuals with spinal cord injury (n=17) | SCI individuals were excluded if they had hypertension, type 2 diabetes, cancer or coronary heart disease | NA | 40 | 40, 100% | 9.2 ± 4.5 | Chronic cervical and thoracic SCI. No information on the type of trauma. | 11.2±6.4 | 31.8 ± 6.8 | Blood lipids, heart rate, blood pressure and blood glucose | 7 |
| Rio et al, 1997 (38) ¥ | CS | Portugal | Outpatients with SCI (no physical activity, n=28) vs Outpatients with spinal cord injury (with physical activity, n=18). | Individuals who had pulmonary or cardiovascular disease were excluded | Individuals taking medication that influenced lipid metabolism were excluded | 46 | 36, 78% | NA | Motor Incomplete thoracic and lumbar SCI. No information on the type of trauma | NA | 32.6 ± 7.4 | Blood lipids | 6 |
| Sadowska-Krępa et al, 2015(24) | CS | Poland | `low-point’ (LP, n=15) and `high-point’ (HP, n=8) Wheelchair rugby players vs sedentary manual wheelchair users (n=9) | NA | NA | 32 | 32, 100% | 13.1±4.7 | Traumatic, chronic cervical SCI | 3 | 32.0 ± 5.0 | Blood lipids, blood glucose, oxidative stress | 7 |
| Schreiber et al, 2014(25) | CS | Brazil | Physically active SCI individuals comprising of athletes (n=23) and sedentary SCI individuals (n=19) | Individuals with diabetes mellitus, systemic hypertension, hyperlipidemia were excluded | NA | 42 | 42, 100% | 8.3 ± 1.0 | Chronic cervical and thoracic SCI. No information on the type of trauma | 11.9 ± 1.4 | 31.5 ± 1.7 | Vascular function, heart rate, Blood lipids, inflammatory markers, Cardiac function and blood glucose | 8 |
| Schreiber et al, 2018(26) | CS | Brazil | Physically active SCI individuals comprising of athletes (n=25) and sedentary individuals with SCI (n=16) | Individuals with diabetes mellitus, systemic hypertension, hyperlipidemia were excluded | NA | 41 | 41, 100% | 9.1 ± 4.0 | Chronic cervical and thoracic SCI. No information on the type of trauma | 11.0±5.9 | 31.9 ± 6.8 | Blood pressure, heart rate, blood glucose, blood lipids, and cardiac function | 7 |
| Schumacher et al, 2009(27) | CS | Germany | Endurance trained paraplegic and tetraplegic individuals (n=25) vs untrained paraplegic and tetraplegic individuals (n=10) | None of the participants had history of autonomic dysreflexia. All participants underwent a medical examination including a blood test to exclude any medical condition that would preclude their participation in the study. | NA | 35 | 35, 100% | NA | Cervical, thoracic, sacral. No information on the type of trauma | >15 | 39.1 ±13.5 | Heart structure and function | 7 |
| Zwiren et al, 1975(29) | CS | Israel | Wheelchair-bound athletes (n=11) who engaged in vigorous training and competed internationally and wheelchair-bound sedentary (n=9) who did not undergo any physical training | NA | NA | 20 | 20,100% | 10.3±NA | Thoracic, no information on the type of trauma | NA | 28.2±9.2 | Cardiorespiratory fitness | 5 |
| Wecht et al. 2006(30) | CS | USA | Individuals (n=9) who participated in aerobic conditioning more than 3 d/week for at least 30 min per session for the last 6 months were considered endurance trained (wheelchair pushing, arm cycle conditioning, and swimming) and sedentary individuals (n=9) with paraplegia were defined as not having participated in regular aerobic conditioning for the past year | Subjects with no known history of cardiovascular disease, pulmonary disease, or diabetes mellitus. | NA | 18 | 18,100% | 11 ±7.2 | Thoracic, no information on type of trauma | ≥150 min/week | 39 ±7.9 | Cardiorespiratory fitness | 7 |
| Tanhoffer et al, 2014(31) | CS | Australia | Sedentary (n = 7) and active, regularly engaged in any exercise program, for at least 150 min/week (n=5) | NA | NA | 13 | 13, 100% | 10 ± 9 | Cervical and thoracic, no information on type of trauma | 123 ± 115 | 40 ± 13 | Cardiorespiratory fitness | 7 |
| Lee et al. 2015(32) | CS | Korea | Subjects that regularly exercised, at least 3 sessions of one or more sports activities per week lasting a minimum of 1 hour (n=24) and sedentary individuals (n=16) | NA | NA | 30 | 30, 100% | >1 year | Thoracic, no information on type of trauma | NA | 44±9.92 | Cardiorespiratory fitness | 7 |
| # We extracted baseline data for inclusion in the meta-analyses. ¥indicates that study did not contribute to meta-analysis | | | | | | | | | | | | | |

Supplemental table 3. Study characteristics of clinical trials

| **Lead Author, Publication Date** | **Location** | **Study design** | **Sample size (N)** | **Percentage of male (%)** | **Duration**  **(weeks)** | **Intervention characteristics** | | **SCI duration**  **(Years)** | **SCI injury type** | **Mean**  **age,** y | **Mean BMI**,  kg/m^2^ | **Health status** | **Overall risk of bias** |
| --- | --- | --- | --- | --- | --- | --- | --- | --- | --- | --- | --- | --- | --- |
|  |  |  |  |  |  | **Intervention** | **Control** |  |  |  |  |  |  |
| Akkurt et al, 2017(39) ¥ | Turkey | Single blind randomized controlled trial | 33 | 29, 88% | 12 | General rehabilitation exercis­es and aerobic exercise with the arm ergometer for 2 sessions/day 5 days/week for 12 weeks. | Only general rehabilitation exercises for 2 sessions/day 5 days/week for 12 weeks. | 3.43± 3.14 | Traumatic, motor complete and incomplete cervical, thoracic and lumbar SCI | 34.7±10.3 | 23.7± 3.8 | Otherwise healthy (i.e., pressure sores,  Bladder infections, cardiovascular diseases or contraindication for exercise) | High |
| *Davis et al, 1987(40) | Canada | Non-randomized clinical trial | 14 | 14, 100% | 16 | The intervention group performed either 20 or 40 mins of arm cranking/ session, 3 times/week at 50% or 70% of their directly measured VO2 peak for a period of 16 weeks. | The control group did not perform the training regimen. | 15.4±2.9 | Motor incomplete thoracic to lumbar SCI (International Stoke Mandeville categories III--V). No information on the type of trauma | 29.8±2.9 | NA | Comorbidities were not reported/discussed. | High |
| *De Almeida et al, 2011(41) | Brazil | Non-randomized clinical trial | 17 | 17, 100% | 8 | Training group had interval swimming training 3 times a week for 6 weeks where they practiced breaststroke of moderate to severe in work periods, and in the backstroke in periods of active recovery | The control group did not participate in any physical activity | NA | Motor complete and incomplete cervical to lumbar SCI (C4-L1;ASIA A-C). No information on the type of trauma | NA | NA | Comorbidities were not reported/discussed. | High |
| Gorgey et al, 2016(42)¥ | USA | Randomized longitudinal clinical trial | 11 | 11, 100% | 16 | The intervention group partook in two different exercise interventions (functional electrical stimulation cycling versus arm cycling ergometer), 5 days/week for 16 weeks (exercise group) | The control group was only involved in two overnight stays/pre-training (baseline and follow-up measurements) and were used to control for the effects of aging with SCI | 5.5 ± 4 | Chronic motor complete SCI (C6–T10; AIS A or B). No information on the type of trauma | 38 ± 9 | 25.7 ± 4.3 | Comorbidities were not reported/discussed). | Some concerns |
| Hicks et al, 2003(43) | Canada | Non-blinded randomized control trial | 34 | NA | 36 | Supervised progressive 90-120 mins exercise training twice weekly for 9 months. Subjects began each exercise session with a warm-up (wheeling around the indoor track or low-intensity arm ergometry) and gentle upper extremity stretching followed an aerobic training, which involved arm ergometry for 15 - 30 min, at an intensity of approximately 70% maximum heart rate | Control group was offered a bi-monthly education session (together with the EX group) on topics including exercise physiology for persons with SCI, osteoporosis after SCI, and relaxation techniques | 9.4±5.9 | Traumatic, motor complete and incomplete cervical to lumbar SCI) | 39.3±10.7 | NA | Otherwise healthy subjects Individuals with ischemic heart disease, unstable angina, dysrhythmia, or autonomic dysreflexia, recent osteoporotic fracture, and tracheostomy were excluded). | High |
| Kim et al, 2015(44) | South Korea | Randomized controlled trial | 15 | 9, 60% | 6 | The 60 mins of exercise/day, 3 days a week for 6 weeks under the supervision of an exercise trainer consisting of 8 mins warm up, 44 mins on of handbike exercise and 8 min cool down | Usual activities | 6.5±3.8 | Motor complete and incomplete cervical and thoracic SCI. No information on the type of trauma | 33.1±5.4 | 21.4±3.2 | Otherwise healthy SCI individuals (e.g., cardiovascular disease, uncontrolled type 2 diabetes and hypertension excluded) | High |
| Kim et al, 2019(45) | South Korea | Non-blinded Randomized control trial | 17 | 12, 65% | 6 | Individualized exercise program designed based on their level of injury, comorbidities, joint conditions, and primary reason for exercise. Participants underwent 60 min exercise/session, three sessions/week. They engaged in 25-min of warm-up consisting of joint exercises, arm ergometer, and stretching, followed by a 30-min exercise program consisting of resistance, circuit and aerobic training and subsequently a 5 min of stretching. | Standard care without exercise | 10.53±6.9 | Motor complete and incomplete cervical, thoracic, and lumbar SCI. No information on the type of trauma | 36.8 ± 6.9 | 21.9 ± 2.82 | Otherwise healthy SCI individuals. Individuals with cardiovascular disease, uncontrolled type 2 diabetes and hypertension, pressure ulcers, and orthopedic problems were excluded | High |
| Nightingale et al, 2017(46) and 2018(47) | United Kingdom | Non-blinded randomized control trial | 21 | 15, 71% | 6 | Participants performed home-based moderate intensity exercise using a portable arm-crank ergometer four times a week. The first exercise session was supervised by an experimenter and extended by 5 minutes per session throughout the first week (i.e. from 30 to 45 minutes). The last stretch of exercise was >36hrs before follow-up laboratory testing. | The control group were encouraged to maintain their normal lifestyle. | 16.29±10.9 | Motor incomplete thoracic SCI. No information on the type of trauma. | 46.8 ± 7.7 | NA | Individuals without acute health issues (i.e., pressure sores, urinary tract infections, and cardiovascular contraindications for testing) or musculoskeletal complaints, and not taking antihyperglycemic medication. | High |
| Ordonez et, 2013(48) ® | Spain | Single blinded randomized controlled trial | 17 | 17, 100% | 12 | The 3 sessions/week, consisting of warming-up (10 to 15 min) followed by arm-crank (20 to 30 min [increasing 2 min and 30s every 3weeks]) at moderate work intensity of 50% to 65% of the heart rate reserve (Starting at 50% and increasing 5% every 3weeks) and by a cooling down period (5 to 10 min). | Individuals matched on age, sex, and injury level who did not take part in any training program | 4.6±0.29 | Traumatic, motor complete SCI below the fifth thoracic level (T5) | 29.9 ± 2.6 | 27.7± 4.0 | Healthy (individuals with smoking habits and alcohol consumers and individuals receiving medication and/or antioxidant  consumption that may interfere with the redox homeostasis were excluded). | Low |
| Rosety-Rodriguez et al, 2014(49) | Spain | Randomized longitudinal clinical trial | 17 | 17, 100% | 12 | Arm cranking exercise program of 3 sessions/week consisting of warm-up (10-15 min), arm crank (20-30 min; increasing 2 min and 30 s every 3 weeks) at a moderate work intensity of 50% to 65% of heart rate reserve (starting at 50% and increasing 5% every 3 weeks), plus cool-down (5-10min). | The control participants completed baseline assessments but did not take part in the training program | 4.6±0.29 | Traumatic, complete SCI at or below T5 | 29.9±3.7 | 27.7±4.2 | Otherwise healthy (individuals with pressure ulcers and/  or coexisting infections, smoking/alcohol intake and receiving medication that may interfere with metabolism, participation in a training program in the 6 months prior to participation in the trial were excluded). | Low |
| Totosy de Zepetnek et al, 2015(50) | Canada | Non-blinded randomized control trial | 23 | 21, 91% | 16 | The training involved ≥20 min of moderate-vigorous aerobic exercise (rating of perceived exertion 3e6 on 10-point scale) and 3-10 repetitions of upper-body strengthening exercises (50%-70% 1 repetition maximum) 2 times per week | Control group maintained existing physical activity levels with no guidance on training intensity. | 12.0 ± 9.9 | Motor complete and incomplete cervical and thoracic SCI. No information on the type of trauma | 41.4±11.6 | 26.5±5.1 | Individuals with any progressive loss of neurologic function within the previous 6 months were excluded. | High |
| *Hopman et al, 1996(51) | The Netherlands | Non-randomized trial | 21 | 18, 85.7% | 24 | The group that was physically active at baseline trained 2h and 15min each week, while the group that was untrained at baseline trained l 1h each week. | The control group did not train during the trial (was classified as sedentary at baseline) | 7.9 ± 7.4 | Cervical lesions, complete and incomplete | 31.8±10.4 | NA | NA | High |
| Lavado et al, 2013(52) | Brazil | Single blind RCT | 42 | 35, 83.3% | 16 weeks | Aerobic physical conditioning with moderate intensity of for one hour, twice or three times a week | Control group maintained their daily life activities. | 4.4± 1.9 | Cervical and thoracic, motor complete and incomplete | 36.3±7.6 | NA | Comorbidities were not reported/discussed | Some concerns |
| ®Pelletier et al, 2015(53) | Canada | Single blind RCT | 23 | 21,91/3% | 16 | Training involved ≥20 min of moderate-vigorous aerobic exercise (rating of perceived exertion 3e6 on 10-point scale) and 3-10 repetitions of upper-body strengthening exercises (50%-70% 1 repetition maximum) 2 times per week | Control group maintained existing physical activity levels with no guidance on training intensity. | 12.0±10.0 | Cervical, thoracic, motor complete and incomplete | 40.4±11.6 | NA | Comorbidities were not reported/discussed | Some concerns |
| *****indicates that a study was a non-randomized clinical trial; **¥**indicates that study did not contribute to meta-analysis (Gorgey et al. used two types of exercise and did not disaggregate data for functional electrical stimulation; Akkurt et al used general rehabilitation exercises as control group); ®partially overlapping population with study by Totosy de Zepetnek et al, 2015 | | | | | | | | | | | | | |

**Supplemental table 4.** Descriptive summary of clinical trials investigating the associations between physical exercise and intermediate cardiovascular risk factors in individuals with SCI

| **Lead author, Publication year** | **Clinical trial characteristics** | | **Main findings** |
| --- | --- | --- | --- |
|  | **Intervention type** | **Control type** |  |
| ¥Akkurt et al, 2017(39) | General rehabilitation exercises and aerobic exercise with the arm ergometer for 12 weeks. | General rehabilitation exercises for 12 weeks. | There were no statistically significant intergroup differences at Weeks 0-6, Weeks 6-12 and Weeks 0-12, both in the intervention group and the control group with regard to metabolic syndrome parameters (TC, TG, HDL, LDL, glucose, waist circumference, SBP, DBP). |
| *Davis et al, 1987(40) | 20 or 40 min of arm cranking per session, 3 times per week at 50% or 70% of their directly measured VO2peak for a period of 16 weeks | did not perform the training regimen | A significant increase of Vo2 peak was observed in intervention group (19% and 31% after 8 and 16 weeks, respectively). LV mass, dimensions and indices of LV performance were unchanged by training, either at rest or during the isometric handgrip. Stroke volumes were significantly increased by 12--16% after training, both in isometric and in rhythmic work; at the highest intensity of arm ergometry, there was also a suggestion of increased cardiac output. |
| *De Almeida et al, 2011(41) | Training group had interval swimming training 3 times a week for 6 weeks where they practiced breaststroke of moderate to severe in work periods, and in the backstroke in periods of active recovery | The control group did not participate in any physical activity | Triglyceride, TC and LDL-cholesterol concentrations were unchanged from the evaluation to the reassessment, in both groups. HDL increased significantly in treatment group, while there was no significant change in control group. |
| ¥Gorgey et al, 2016(42) | The intervention group took part in two different exercise interventions (functional electrical stimulation cycling versus arm cycling ergometer), 5 days/week for 16 weeks (exercise group) | The control group was only involved in two overnight stays/pre-training (baseline and follow-up measurements) and were used to control for the effects of aging with SCI. | There were no changes in the lipid profile in either the exercise or the control groups following the post-intervention or in the follow-up assessment visits. |
| Hicks et al, 2003(43) | Supervised progressive 90-120 min exercise training twice weekly for 9 months. Subjects began each exercise session with a warm-up (wheeling around the indoor track or low-intensity arm ergometry) and gentle upper extremity stretching followed an aerobic training, which involved arm ergometry for 15 - 30 min, at an intensity of approximately 70% maximum heart rate | Control group was offered a bi-monthly education session (together with the exercise group) on topics including exercise physiology for persons with SCI, osteoporosis after SCI, and relaxation techniques | There were no differences between groups in resting measures of heart rate, systolic or diastolic pressure at baseline, nor were there any changes in these variables in either group over the 9 months. Subjects with tetraplegia had similar resting HRs as those with paraplegia, but significantly lower systolic and diastolic blood pressures; there was no effect of time or group assignment on these measures. |
| Kim et al, 2015(44) | 60-minute exercise sessions on an indoor hand-bike. Participants in the exercise group exercised 3 times per week for 6 weeks. | Usual activities | Participation in a six-week exercise program significantly decreased BMI (baseline: 22.0±3.7 m/kg2 vs. post-intervention: 21.7±3.5 m/kg2), fasting insulin (baseline: 5.4±2.9 µU/ml vs. post-intervention: 3.4±1.5 µU/ml,), and HOMA-IR (baseline: 1.0±0.6 vs. post-intervention: 0.6±0.3) levels compared to the control group. HDL-C level (baseline: 42.4±11.5 mg/dl vs. post-intervention: 46.1±12.3 mg/dl) increased significantly after training. No significant changes in glucose, TC, TG, or LDL-C levels were observed in the exercise group. VO2 peak (baseline: 16.8±7.2 ml/kg/min vs. post-intervention: 21.2±9.1 ml/kg/min) increased significantly in the exercise group compared to the control group (mean difference vs. control, -2.9 ml/kg/min). |
| Kim et al, 2019(45) | Daily exercise program consisted of a 25-min warm-up consisting of 5 min of joint exercises, 15 min of exercise on an arm ergometer, and 5 min of stretching, followed by a 30-min exercise program (resistance, circuit, and aerobic training), and a 5 min of cool down (stretching), but the contents of the 30-min exercise were customized for each individual depending on the comorbidities and other factors. | Standard care | The 6-week exercise program significantly decreased the average fasting insulin (baseline: 7.5±4.7 µU/ml vs. post intervention: 4.5±2.2 µU/ml, p<0.05) and HOMA-IR (baseline: 1.5±1.0 vs. post-intervention: 0.9±0.4, p<0.05) in the exercise group, whereas there was no change in control group (between group difference, mean fasting insulin: −3.2 µU/ml, p=0.003; mean HOMA-IR: −0.66, p=0.001). HDL-C has increased in exercise group and decreased in control group during the follow up (pre-post difference was 5.5 mg/dl ± 8.0 in exercise group and -1.7mg/dl ± 1.9 in control group, p=0.021). There were no differences in glucose, TC and LDL |
| Nightingale et al, 2017(46) and 2018(54) | Moderate-intensity upper-body home-based exercise. Four times per week on a portable desktop arm-crank ergometer in their own home. The duration of each exercise session was extended by 5 min per session throughout the first week (i.e., from 30 to 45 min). The exercise intensity was also increased from ~60% V˙O2peak during the first 3 weeks to ~65% V˙ O2peak for the final 3 week | lifestyle maintenance | Compared with controls, intervention group significantly decreased serum fasting insulin (Δ, 3.1 ±10.7 pmolL-^1^ for control and -12.7±18.7 pmolL-^1^ for intervention) and homeostasis model assessment of insulin resistance (HOMA2-IR; Δ, 0.06 ± 0.20 for control and -0.23 ± 0.36 for intervention). Adipose tissue metabolism, composite insulin sensitivity index (C-ISIMatsuda), and other cardiovascular disease risk biomarkers were not different between groups. The exercise group also increased the V O2 peak (Δ, 3.4 ml·kg-1·min-1). |
| Ordonez et, 2013(48) | Twelve-week arm-cranking exercise program, 3 sessions/week, consisting of warming-up (10 to 15 min) followed by arm-crank (20 to 30 min [increasing 2min and 30 s every 3wk]) at moderate work intensity of 50% to 65% of the heart rate reserve. (Starting at 50% and increasing 5% every 3weeks) and by a cooling down period (5 to 10 min). | Individuals matched on age, sex, and injury level who did not take part in any training program | Both total antioxidant status (0.64±0.2mmol/L vs 0.88±0.1mmol/L) and erythrocyte GPX activity (23.6±2.4U/g haemoglobin vs 27.8±2.2U/g haemoglobin) were significantly increased at the end of the training program. Lipid peroxidation, expressed as plasmatic levels of malondialdehyde, was significantly reduced (0.48±0.13mmol/L vs 0.35±0.11mmol/L). Similarly, protein oxidation, expressed as plasmatic carbonyl group level, was decreased after exercise (1.92±0.3nmol/mg protein vs 1.33±0.2nmol/mg). In the control group, no significant changes in any of the tested parameters were found. |
| Rosety-Rodriguez et al, 2014(49) | The intervention group was exposed to a 12-week arm cranking exercise program of 3 sessions/week consisting of warm-up (10-15 min), arm crank (20-30 min; increasing 2 min and 30 s every 3 weeks) at a moderate work intensity of 50% to 65% of heart rate reserve (starting at 50% and increasing 5% every 3 weeks), plus cool-down (5-10 min). | The control participants completed baseline assessments but did not take part in the training program | When compared with baseline, plasma levels of leptin, TNF-a, and IL-6 were significantly decreased in the intervention group. In contrast, no significant changes were found in plasma concentrations of adiponectin and PAI-1. |
| Totosy de Zepetnek et al, 2015(50) and Pelletier et al 2015(53) | Training involved ≥20 min of moderate-vigorous aerobic exercise (rating of perceived exertion 3e6 on 10-point scale) and 3-10 repetitions of upper-body strengthening exercises (50%-70% 1 repetition maximum) 2 times per week | Control group maintained existing physical activity levels with no guidance on training intensity. | When implemented as part of a supervised training program, the physical activity guidelines for adults with SCI has a positive influence on some aspects of body composition and carotid vascular health. Despite these benefits, 16 weeks of adherence to the physical activity guidelines did not elicit changes in other CVD risk factors. There was a significant increase in peak aerobic capacity (relative VO2peak: 17.2%, absolute VO2peak: 9.9%) and submaximal power output (26.3%) in the control group only. |
| *Hopman et al, 1996 | The group that was physically active at baseline trained 2h and 15min each week, while the group that was untrained at baseline trained l 1h each week. No further information | The control group did not train during the trial (was classified as sedentary at baseline). | During maximal exercise, peak power output and peak oxygen uptake were significantly higher in trained group (49.9 W and 14.2ml-min-l'kg-~ respectively) as compared to untrained group (20.7W and 8.8ml'min-l'kg -1 respectively) and sedentary group (15.9 W and 7.4 ml.min-1 .kg-~ respectively), whereas all other peak responses showed tendencies to be higher in trained group. |
| Lavado et al, 2013(52) | Aerobic physical conditioning with moderate intensity | Control group maintained their daily life activities. | The increase of oxygen consumption in the intervention group compared to the control group was observed only at the end of the programme. In the values before and after the training period of the intervention group significant differences were also observed. |
| *****indicates that a study was a non-randomized clinical trial; **¥**indicates that study did not contribute to meta-analysis (Gorgey et al. used two types of exercise and did not disaggregate data for functional electrical stimulation; Akkurt et al used general rehabilitation exercises as control group)  **Abbreviations**: BMI: body mass index; TC: total cholesterol; LDL: Low density lipoprotein; HDL: High density lipoprotein: HOMA-IR: Homeostatic model assessment for insulin resistance | | | |

**Supplemental table 5.** Cardiorespiratory fitness assessment in observational studies and clinical trials

| Lead author, year of publication | Study/exercise protocol |
| --- | --- |
| *¥Akkurt et al, 1997 | Exercise capacity was measured in accordance to the cardiopulmonary function test and an arm ergometer test in a sitting position. A power output of 30 W was used for two minutes and then increased by 10 W every two minutes. Peak Oxygen uptake was analyzed breath by breath every 30 seconds |
| Bhambhani et al, 1995 | Each subject completed an incremental velocity test to volitional exhaustion according to the following protocol: in the untrained group, the test was initiated at a wheeling velocity of 5 km/hr for 2 min, followed by increments of 2 km/ hr every 2 min, until the required wheeling velocity could not be maintained (i.e., volitional fatigue); in the endurance-trained group, a similar protocol was used except that the test was initiated at wheeling velocity of 10 km/hr |
| Dallmeijer et al, 1997 | Peak oxygen uptake was measured on a computer-controlled, stationary wheelchair ergometer, which allowed for direct measurements of torques applied on the rim, as well as resultant velocity of the wheels, for the left and right side separately. The  wheel and hand rim radii were 0.31 and 0.26 m, respectively. Ergometer settings were individually adjusted |
| ¥Davis et al, 1987_1_ | Peak oxygen uptake (Vo2 peak) was determined using open-circuit spirometry during a progressive test (1 min stages of 8.5 W) to volitional fatigue. |
| Davis et al, 1988 | Peak oxygen intake (VO2 peak) was evaluated using a multistage arm cranking protocol (Monark model 881 arm crank ergometer). Ergometer was operated at a cadence of 80 rev*min-1 beginning at 0W and increasing the power output by 8.5 W every minute. |
| Huonker et al, 1998 | Warm-up period: incremental exercise test after a 5-min resting period in a special wheelchair ergometer (Ergotronic 9000, Sopur) followed by an unloaded wheelchair exercise (3 min).Heart rate was continuously monitored via electrocardiogram. All spirometric measurements were done using an automated gas analyzer (Oxycon Sigma, Mijnhardt, The Netherlands). Oxygen uptake was averaged over 10-s intervals.  Incremental wheelchair exercise test was performed in all subjects with 20-W workload initially. Exercise intensity was increased by 10 W every 3 min until exhaustion. Subjects were verbally encouraged to give maximal effort.  Mean exercise time ranged from 11.9 ± 5.3 min in sedentary individuals to 20.5 ± 4.8 min in physically active.  Peak oxygen uptake (˙VO2peak) and peak heart rate were defined as the highest values for the oxygen uptake and the heart rate achieved by a subject until exhaustion. |
| Ingles et al, 2016 | **The graded exercise test (GET)** was performed using an arm-ergometer (Monark 881E, Stockholm, Sweden).  The warm-up, 10 min pedaling with their arms with a power of 0  watts. Immediately, the GET started. They were asked to maintain the same pedal cadence (60 r.p.m.) during the test. Participants started with a initial workload of 30 watts, and the clinical researchers increased the power 15 watts every 2 min until volitional exhaustion.  The VO2 peak was taken as the highest VO2 value and was stated as being achieved by three of the following four end point criteria: (i) blood lactate concentration of 7–8 mM, (ii) respiratory Quotient > 1.1, (iii) plateau of oxygen consumption despite increasing workload and (iv) achievement of 90% of age-adjusted maximal heart rate |
| *Kim et al, 2015 | All participants underwent maximal graded exercise tests according to the RAMP protocol. The criteria for VO2 peak were as follows: achievement of VO2 plateau despite increased exercise intensity; R > 1.15; heart rate achieved or an RPE grade 19–20 |
| *Lavado et al, 2013 | The protocol used in the ergometry was progressive loading with an increase of 150 kg in each 3 minutes of cycling followed by 1 minute of rest in the final of each stage. During each rest phase the heart rate and blood pressure were measured. For security reasons, there was the option to work with a heart rate of 85–90% of the maximum, accompanied by the perceived exertion scale. The computed value to be analysed was the estimated peak oxygen consumption |
| Lee et al, 2015 | Arm ergometer 917900 (Lode BV, Groningen, The Netherlands) was used to assess the fitness. The study started with a warm-up exercise for three minutes, and the intensity was increased at two-minute intervals until exhaustion. The initial loading was 30 W and the speed was maintained at 60 revolutions per minute (rpm). The loading was increased by 15 W for each exercise stage and the protocol proceeded for 7 stages excluding the warm-up exercise. |
| Lovell et al, 2012 | The upper body VO2 peak test was conducted on a modified electromagnetically braked cycle ergometer (EE). The EE was fixed to a table with the table fixed to the ground to prevent any movement in the EE during the  VO2 peak test. A modified chair was also fixed to the ground and participants were advised to keep their feet flat on the ground and remain seated throughout the test. For the trained SCI men the test began with a 2-min warm-up at a constant power of 45 W. This was followed by a ramp protocol beginning at 60 W with increments of 12 W every minute (1 W every 5 s). The untrained SCI men began with a 2-min warm-up at a constant power of 30 W and a ramp protocol beginning at 45 W with increments of 6 W every minute (1 W every 10 s). All participants hand cycled at a self-selected crank rate until volitional exhaustion or until fly wheel revolutions dropped below 60 rpm. |
| Maggioni et al, 2012 | An incremental exercise test procedure was performed by an adapted wheelchair ergometer (Ergotronic 4000, Sopur,  Heidelberg, Germany). The exercise protocol began at an initial velocity of 2 km h1 and continued with 3-min steps, with a speed increment of 2 km h1 per step; the test was stopped at the volitional exhaustion. The maximal velocity achieved and the peak O2 consumption (pVO2) and the resting and peak heart rate (HR) were reported. |
| *Nightingale et al, 2018 | Participants performed a discontinuous, incremental submaximal arm-crank ergometry test on the same portable desktop ergometer provided to them during the intervention. Following a short rest, peak oxygen uptake (Vo2peak), and workload were measured at the point of volitional exhaustion during a continuous, incremental exercise protocol,24 performed on an electrically braked arm-crank ergometer |
| *Pelletier et al, 2015 | To measure peak oxygen consumption (VO2peak), participants performed a symptom-limited, graded exercise test on an arm cycle ergometer (Lode B.V., Groningen, the Netherlands) at baseline (pre-training) and 16 weeks (post-training). Resistance was increased by 5 W min− 1 for participants with tetraplegia and 10 W min− 1 for participants with paraplegia.11 In some cases, this protocol was modified to ensure the test was 8–12 min in duration. Participants were asked to continue until volitional fatigue or they were unable to maintain a cadence of 40 r.p.m |
| Tanhoffer et al, 2014 | An arm-crank maximal test was performed on the second visit to assess their fitness level. No further details were provided |
| Wecht et al. 2006 | An arm cycle ergometry (ACE) ramp protocol was used to elicit peak V˙ O2. ramp protocol was then initiated using a 12-WIminj1 increment for the unfit subjects and a 24-W min-1 increment for the fit subjects; both groups were asked to maintain a pedal cadence of 60 rpm. The incremental ramp protocols differed between the groups in an effort to keep the test duration under 10 min. Test termination criteria included an RER ≥ 1.1, V˙ O2 plateau with increasing work, failure to maintain the 60 rpm, and/or volitional fatigue |
| Zwiren et al, 1975 | Monark ergometer was modified for use with the arms. Submaximal test: the loads were 75 kmp/min, 150 kmp/min and 225 kmp/min, each lasting 2 minutes. Pedalling rate was 50 RPM.  Maximal test: the initial load was selected according to the individual's HR during submaximal test, thus the overall test duration was between 5 and 7 minutes. The load was increased every to minutes by 150 kmp/min until exhaustion. The highest O2 consumption (Vo2 max arm) was determined as the subject's aerobic capacity during arm exercise. |
| *Indicates randomized clinical trials. ¥indicates that study did not contribute to meta-analysis  _1_ Indicates Non-randomized clinical trial | |

**Supplemental table 6**. Descriptive characteristics of cross-sectional studies included in the meta-analysis (n=29)

| Characteristic | **No. of studies** | **References** |
| --- | --- | --- |
| Physical activity type^*^ | | |
| Professional para-athletes | 11 | Brenes et al, 1986(3), Currie et al, 2015(5), De Rossi et al, 2014(10), Dearwater et al, 1986(11), Hübner-Woźniak et al, 2010(16), Hübner-Woźniak et al, 2012(17), Huonker et al, 1998(18), Paim et al, 2019(23), Schreiber et al, 2014(25), Schreiber et al, 2018(26), Zwiren et al, 1975(29), |
| Individuals with SCI engaged in physical activity | 18 | Currie et al, 2017(6), Lovell et al, 2012(20), Maggioni et al, 2012(21), Schumacher et al, 2009(27)  Bell et al, 2011(1), Buchholz et al, 2009^3^ ,  Dallmeijer et al, 1997 ^86^, D'Oliveira et al, 2014(12), Flank et al, 2014^10^ , Koury et al, 2013 (19), Sadowska-Krępa et al, 2015(24), Bhambhani et al, 1995(2), Davis et al, 1988(9), Ingles et al, 2016(28), Wecht et al, 2006(30), Tanhoffer et al, 2014(31), Lee et al, 2015(32), Dallmeijer et al, 1997(8) |
| Level of SCI | | |
| Cervical | 10 | Currie et al, 2015(5), Currie et al, 2017(6), Dallmeijer et al, 1997(7), D'Oliveira et al, 2014(12), Hübner-Woźniak et al, 2010(16), Hübner-Woźniak et al, 2012(17), Koury et al, 2013 (19), Sadowska-Krępa et al, 2015(24), Bhambhani et al, 1995(2), Dallmeijer et al, 1997(8) |
| Thoracic | 7 | Flank et al, 2014(13), Huonker et al, 1998(18), Lovell et al, 2012(20), Ingles et al 2016(28), Zwiren et al, 1975(29), Wecht et al, 2006(30), Lee et al, 2015(32) |
| Cervical and thoracic | 8 | Bell et al, 2011(1), De Rossi et al, 2014(10), Maggioni et al, 2012(21), Paim et al, 2019(23), Schreiber et al, 2014(25), Schreiber et al, 2018(26), Schumacher et al, 2009(27), Tanhoffer et al, 2014(31) |
| Thoracic and Lumbar | 1 | Davis et al, 1988(9) |
| Not reported | 3 | Brenes et al, 1986(3), Buchholz et al, 2009(4), Dearwater et al, 1986(11), |
| SCI duration, years | | |
| ≤median (10.7) (Q1, Q3:5.8, 19.2) | 10 | Dallmeijer et al, 1997(7), De Rossi et al, 2014(10), D'Oliveira et al, 2014(12), Hübner-Woźniak et al, 2010(16), Paim et al, 2019(23), Schreiber et al, 2014(25), Schreiber et al, 2018(26), Dallmeijer et al, 1997(8), Zwiren et al, 1975(29) , Tanhoffer et al, 2014(31), |
| >median (10.7) (Q1, Q3:5.8, 19.2) | 10 | Bell et al, 2011(1), Buchholz et al, 2009(4), Currie et al, 2015(5), Currie et al, 2017^5^, Flank et al, 2014(13), Koury et al, 2013 (19), Sadowska-Krępa et al, 2015(24), Bhambhani et al, 1995(2), Ingles et al, 2016(28), Davis et al, 1988(9), |
| Not reported | 9 | Brenes et al, 1986(3), Dearwater et al, 1986(11), Hübner-Woźniak et al, 2012(17), Huonker et al, 1998^14^, Lovell et al, 2012(20), Maggioni et al, 2012(21), Schumacher et al, 2009(27), Lee et al, 2015(32), Wecht et al, 2006(30) |
| Sex | | |
| Men only | 23 | Currie et al, 2015(5), Currie et al, 2017^5^,  Dallmeijer et al, 1997(7), De Rossi et al, 2014(10), Dearwater et al, 1986(11), D'Oliveira et al, 2014(12), Hübner-Woźniak et al, 2010(16), Hübner-Woźniak et al, 2012(17), Huonker et al, 1998(18), Koury et al, 2013 (19), Lovell et al, 2012(20), Maggioni et al, 2012(21), Paim et al, 2019(23), Sadowska-Krępa et al, 2015(24), Schreiber et al, 2014(25), Schreiber et al, 2018^25^ , Schumacher et al, 2009(27), Bhambhani et al, 1995(2), Davis et al, 1988(9), Zwiren et al, 1975(29), Wecht et al, 2006(30), Tanhoffer et al, 2014(31), Lee et al, 2015(32) |
| Women only | 0 | --- |
| Both | 6 | Bell et al, 2011(1), Brenes et al, 1986(3), Buchholz et al, 2009(4), Flank et al, 2014(13), Ingles et al, 2016(28), Dallmeijer et al, 1997(8) |
| Sex not reported | 0 | --- |
| Study size | | |
| ≤30 | 17 | Currie et al, 2015(5), Currie et al, 2017(6), Dallmeijer et al, 1997(7), D'Oliveira et al, 2014(12), Hübner-Woźniak et al, 2010(16), Hübner-Woźniak et al, 2012(17), Koury et al, 2013 (19), Lovell et al, 2012(20), Maggioni et al, 2012(21), Sadowska-Krępa et al, 2015(24), Bhambhani et al, 1995(2), Davis et al, 1988(9), Ingles et al, 2016(28), Zwiren et al, 1975(29), Wecht et al, 2006(30), Tanhoffer et al, 2014(31), Dallmeijer et al, 1997(8) |
| >30 | 12 | Brenes et al, 1986(3), Buchholz et al, 2009(4), De Rossi et al, 2014(10), , Dearwater et al, 1986(11), Bell et al, 2011(1), Flank et al, 2014(13) , Huonker et al, 1998(18), Paim et al, 2019(23), Schreiber et al, 2014(25), Schreiber et al, 2018(26), Schumacher et al, 2009(27), Lee et al, 2015(32) |
| Age, years | | |
| ≤median (33.0) (Q1,Q3: 27.7, 47.8) | 13 | Brenes et al, 1986(3), De Rossi et al, 2014(10), Dearwater et al, 1986(11), D'Oliveira et al, 2014(12), Hübner-Woźniak et al, 2010(16), Hübner-Woźniak et al, 2012(17), Paim et al, 2019(23), Sadowska-Krępa et al, 2015(24), Schreiber et al, 2014(25), Schreiber et al, 2018(26), Davis et al, 1988(9), Zwiren et al, 1975(29), Bhambhani et al, 1995(2) |
| >median (33.0) (Q1,Q3: 27.7, 47.8) | 16 | Bell et al, 2011(1), Buchholz et al, 2009(4), Currie et al, 2015(5), Currie et al, 2017(6), Dallmeijer et al, 1997(7), Flank et al, 2014(13), Huonker et al, 1998(18), Koury et al, 2013 (19), Lovell et al, 2012(20), Maggioni et al, 2012(21), Schumacher et al, 2009^26^ , Ingles et al, 2016(28), Dallmeijer et al, 1997(8), Tanhoffer et al, 2014(31), Lee et al, 2015(32), Wecht et al, 2006(30) |
| Location | | |
| Europe | 9 | Dallmeijer et al, 1997(7), Flank et al, 2014(13), Hübner-Woźniak et al, 2010(16), Hübner-Woźniak et al, 2012(17), Huonker et al, 1998(18), Maggioni et al, 2012(21), Sadowska-Krępa et al, 2015(24), Schumacher et al, 2009(27), Ingles et al, 2016(28), Dallmeijer et al, 1997(8) |
| North America | 9 | Bell et al, 2011(1), Brenes et al, 1986(3), Buchholz et al, 2009(4), Currie et al, 2015(5), Currie et al, 2017(6), Dearwater et al, 1986(11), Bhambhani et al 1995(2), Davis et al, 1988(9), Wecht et al, 2006(30) |
| South America | 6 | De Rossi et al, 2014(10), D'Oliveira et al, 2014(12), Koury et al, 2013 (19), Paim et al, 2019(23), Schreiber et al, 2014(25), Schreiber et al, 2018(26) |
| Asia | 1 | Lee et al, 2015(32) |
| Africa | 0 | ---- |
| Middle East | 1 | Zwiren et al, 1975(29) |
| Australia | 2 | Lovell et al, 2012(20), Tanhoffer et al, 2014(31) |
| Outcomes | | |
| Blood lipids | 9 | Brenes et al, 1986(3), Buchholz et al, 2009(4), Dallmeijer et al, 1997(7), De Rossi et al, 2014(10), Dearwater et al, 1986(11), Flank et al, 2014(13), Hübner-Woźniak et al, 2010(16), Paim et al, 2019(23), Sadowska-Krępa et al, 2015(24) |
| Glucose homeostasis | 8 | Buchholz et al, 2009(4), Dearwater et al, 1986(11), D'Oliveira et al, 2014(12), Flank et al, 2014(13), Koury et al, 2013 (19), Paim et al, 2019(23), Sadowska-Krępa et al, 2015(24), De Rossi et al, 2014(10) |
| Blood pressure | 8 | Bell et al, 2011(1), Currie et al, 2015(5), Currie et al, 2017^5^, De Rossi et al, 2014(10), Flank et al, 2014(13), Paim et al, 2019(23), Huonker et al, 1998(18), Buchholz et al, 2009(4) |
| Heart rate | 7 | Currie et al, 2015(5), Currie et al, 2017(6), Huonker et al, 1998(18), Lovell et al, 2012(20), De Rossi et al, 2014(10), Maggioni et al, 2012(21), Paim et al, 2019^21^, |
| Cardiac structure | 4 | Currie et al, 2017(6), De Rossi et al, 2014(10), Maggioni et al, 2012(21), Schumacher et al, 2009(27) |
| Global systolic and diastolic function | 4 | Currie et al, 2017(6), De Rossi et al, 2014(10), Huonker et al, 1998(18), Maggioni et al, 2012(21) |
| Inflammatory markers (HsCRP) | 3 | Buchholz et al, 2009(4), Koury et al, 2013 (19), Schreiber et al, 2014(25) |
| Oxidative stress (Catalase) | 2 | Hübner-Woźniak et al, 2012(17), Sadowska-Krępa et al, 2015(24) |
| Atherosclerosis/Carotid intima media thickness (IMT) | 2 | Bell et al, 2011(1), Schreiber et al, 2018(26) |
| Cardiorespiratory fitness | 11 | Bhambhani et al, 1995(2), Davis et al, 1988(9), Dallmeijer et al, 1997(8), Ingles et al, 2016(28), Wecht et al, 2006(30), Tanhoffer et al, 2014(31), Zwiren et al, 1975(29), Lee et al, 2015(32), Huonker et al, 1998(55), Maggioni et al, 2012(21), Lovell et al, 2012(20) |
| Study quality | | |
| Poor | 0 | ---- |
| Moderate | 27 | Bell et al, 2011(1), Brenes et al, 1986(3), Currie et al, 2017(6), De Rossi et al, 2014^72^, Dearwater et al, 1986(11), Flank et al, 2014(13), Maggioni et al, 2012(21), Paim et al, 2019(23), Sadowska-Krępa et al, 2015(24), , Schreiber et al, 2018(26), Schumacher et al, 2009(27) ,Currie et al, 2015(5), Dallmeijer et al, 1997(7), D'Oliveira et al, 2014(12), Hübner-Woźniak et al, 2010(16), Hübner-Woźniak et al, 2012(17), Huonker et al, 1998(18), Koury et al, 2013^16^, Lovell et al, 2012(20), Dallmeijer et al, 1997(8), Bhambhani et al, 1995(2), Davis et al, 1988(9), , Ingles et al, 2016(28), Wecht et al, 2006(30), Tanhoffer et al, 2014(31), Zwiren et al, 1975(29), Lee et al, 2015(32) |
| Good | 2 | Buchholz et al, 2009^3^, Schreiber et al, 2014(25) |
| Para-athletes were defined as individuals with SCI engaged in regular training sessions or as professionally trained individuals participating in national and international competitions; Individuals with SCI engaged in physical activity refers to SCI individuals who were classified as physically active but without professional component across different studies. | | |
|  | | |

**Supplemental table 7.** Mean values of cardiovascular risk factors, cardiac structure and function and cardiorespiratory fitness in physically active and control group (observational studies)

| **Author, year of publication** | **Outcome, unit** | **Active SCI individuals** | | | **Inactive SCI individuals** | | | **Outcome, unit** |
| --- | --- | --- | --- | --- | --- | --- | --- | --- |
|  |  | **Number** | **Mean** | **SD** | **Number** | **Mean** | **SD** |  |
| de Rossi et al, 2014^2^ | Glucose, mg/dL | 29 | 80.2 | 5.39 | 29 | 82.8 | 8.62 | Glucose, mg/dL |
| Oliveira et al, 2014 | Glucose, mg/dL | 14 | 84.3 | 6.5 | 8 | 89 | 9.5 | Glucose, mg/dL |
| Dearwater et al, 1986 | Glucose, mg/dL | 17 | 79.6 | 10.4 | 77 | 82.8 | 11.9 | Glucose, mg/dL |
| Koury et al, 2013 | Glucose, mg/dL | 13 | 84.3 | 6.2 | 9 | 89 | 8.9 | Glucose, mg/dL |
| Sadowska-Krepa et al, 2015 |  | 15 | 91.9 | 13.3 | 9 | 89.5 | 12.8 | Glucose, mg/dL |
| Sadowska-Krepa et al, 2015 |  | 8 | 91.2 | 6.8 | 9 | 89.5 | 12.8 | Glucose, mg/dL |
| Buchholz et al, 2009 |  | 5 | 97.7 | 19.8 | 11 | 98.4 | 22.5 | Glucose, mg/dL |
| Buchholz et al, 2009 |  | 15 | 91.5 | 13.9 | 10 | 101.8 | 29.7 | Glucose, mg/dL |
| Paim et al, 2019 |  | 23 | 79.1 | 4.9 | 17 | 84.2 | 9.5 | Glucose, mg/dL |
| Flank et al, 2014 |  | 27 | 90.1 | 18 | 107 | 93.7 | 27 | Glucose, mg/dL |
| Oliveira et al, 2014 |  | 14 | 1.1 | 0.5 | 8 | 1.7 | 0.7 | HOMA-IR |
| Koury et al, 2013 |  | 13 | 1.1 | 0.7 | 9 | 0.7 | 0.7 | HOMA-IR |
| Sadowska-Krepa et al, 2015 |  | 15 | 1.5 | 0.9 | 9 | 1.69 | 0.9 | HOMA-IR |
| Sadowska-Krepa et al, 2015 |  | 8 | 1.7 | 1.1 | 9 | 1.69 | 0.9 | HOMA-IR |
| Buchholz et al, 2009 |  | 5 | 1.44 | 1.36 | 11 | 3.39 | 2.68 | HOMA-IR |
| Buchholz et al, 2009 |  | 15 | 1.94 | 1.84 | 10 | 2.22 | 1.94 | HOMA-IR |
| Oliveira et al, 2014 |  | 14 | 8.3 | 4.4 | 8 | 13.4 | 5.5 | Insulin, µU/mL |
| Dearwater et al, 1986 |  | 17 | 10.5 | 15 | 77 | 8.9 | 8.6 | Insulin, µU/mL |
| Koury et al, 2013 |  | 13 | 8.3 | 4.4 | 9 | 13.4 | 5.5 | Insulin, µU/mL |
| Sadowska-Krepa et al, 2015 |  | 15 | 6.5 | 2.9 | 9 | 7.4 | 3.1 | Insulin, µU/mL |
| Sadowska-Krepa et al, 2015 |  | 8 | 8.1 | 3.9 | 9 | 7.4 | 3.1 | Insulin, µU/mL |
| Buchholz et al, 2009 |  | 5 | 5.64 | 3.89 | 10 | 12.2 | 9.76 | Insulin, µU/mL |
| Buchholz et al, 2009 |  | 14 | 7.17 | 5.73 | 10 | 8.6 | 6.84 | Insulin, µU/mL |
| Brenes et al, 1986 |  | 17 | 151 | 27 | 56 | 172 | 39 | TC, mg/dl |
| Brenes et al, 1986 |  | 5 | 145 | 23 | 10 | 154 | 28 | TC, mg/dl |
| Buchholz et al, 2009 |  | 5 | 182.2 | 37.5 | 11 | 181.5 | 40.5 | TC, mg/dl |
| Buchholz et al, 2009 |  | 14 | 192.7 | 42.9 | 10 | 193.8 | 41.3 | TC, mg/dl |
| Dallmeijer et al, 1997^2^ |  | 11 | 177.88 | 42.54 | 13 | 189.5 | 50.27 | TC, mg/dl |
| Dearwater et al, 1986 |  | 17 | 151 | 27.3 | 77 | 170 | 40.5 | TC, mg/dL |
| Flank et al, 2014 |  | 27 | 88.3 | 12.6 | 107 | 86.4 | 18 | TC, mg/dl |
| Hübner-Woźniak et al, 2010 |  | 10 | 70.8 | 8.1 | 10 | 78 | 9 | TC, mg/dl |
| Sadowska-Krepa et al, 2015 |  | 15 | 151.6 | 22.7 | 9 | 160.5 | 22.4 | TC, mg/dL |
| Sadowska-Krepa et al, 2015 |  | 8 | 171.5 | 37.8 | 9 | 160.5 | 22.4 | TC, mg/dL |
| Brenes et al, 1986 |  | 17 | 42.7 | 6.9 | 56 | 34.8 | 6.8 | HDL, mg/dl |
| Brenes et al, 1986 |  | 5 | 50.8 | 4 | 10 | 40.9 | 6.8 | HDL, mg/dl |
| Buchholz et al, 2009 |  | 5 | 44 | 10 | 11 | 48.3 | 18.5 | HDL, mg/dl |
| Buchholz et al, 2009 |  | 14 | 44.4 | 7.7 | 10 | 50.6 | 13.1 | HDL, mg/dl |
| Dallmeijer et al, 1997^2^ |  | 11 | 43.7 | 8.12 | 13 | 36.74 | 7.73 | HDL, mg/dl |
| de Rossi et al, 2014^2^ |  | 29 | 39.4 | 8.09 | 29 | 39.7 | 6.47 | HDL, mg/dl |
| Dearwater et al, 1986 |  | 17 | 42.7 | 6.9 | 77 | 34.2 | 7.9 | HDL, mg/dl |
| Flank et al, 2014 |  | 27 | 19.8 | 5.4 | 107 | 21.6 | 7.2 | HDL, mg/dl |
| Hübner-Woźniak et al, 2010 |  | 10 | 24.9 | 7.2 | 10 | 20.2 | 3.6 | HDL, mg/dl |
| Paim et al, 2019^2^ |  | 23 | 40 | 8 | 17 | 41 | 6 | HDL, mg/dl |
| Sadowska-Krepa et al, 2015 |  | 15 | 44.1 | 11 | 9 | 36.3 | 6.2 | HDL, mg/dl |
| Sadowska-Krepa et al, 2015 |  | 8 | 47.8 | 14.7 | 9 | 36.3 | 6.2 | HDL, mg/dl |
| Buchholz et al, 2009 |  | 4 | 107.7 | 41.3 | 11 | 106.9 | 45.6 | LDL, mg/dL |
| Buchholz et al, 2009 |  | 14 | 119.7 | 37.1 | 9 | 114.7 | 44.4 | LDL, mg/dL |
| Dallmeijer et al, 1997^2^ |  | 11 | 112.14 | 34.8 | 13 | 123.74 | 42.54 | LDL, mg/dL |
| de Rossi et al, 2014^2^ |  | 29 | 98.8 | 27.49 | 29 | 104.9 | 29.65 | LDL, mg/dL |
| Flank et al, 2014 |  | 27 | 57.7 | 10.8 | 107 | 54.1 | 18 | LDL, mg/dL |
| Hübner-Woźniak et al, 2010 |  | 10 | 39.6 | 7.2 | 10 | 48.5 | 22.3 | LDL, mg/dL |
| Paim et al, 2019^2^ |  | 23 | 99 | 27 | 17 | 118 | 36 | LDL, mg/dL |
| Sadowska-Krepa et al, 2015 |  | 15 | 83.4 | 25.4 | 9 | 102.4 | 23 | LDL, mg/dL |
| Sadowska-Krepa et al, 2015 |  | 8 | 99.9 | 35.9 | 9 | 102.4 | 23 | LDL, mg/dL |
| Brenes et al, 1986 |  | 17 | 115 | 84 | 56 | 129 | 54 | TG, mg/dl |
| Brenes et al, 1986 |  | 5 | 72 | 30 | 10 | 90 | 39 | TG, mg/dl |
| Buchholz et al, 2009 |  | 5 | 69.5 | 61.4 | 11 | 56.8 | 28.2 | TG, mg/dl |
| Buchholz et al, 2009 |  | 14 | 62.5 | 29.3 | 10 | 64.9 | 47.5 | TG, mg/dl |
| Dallmeijer et al, 1997^2^ |  | 11 | 118.7 | 59.34 | 13 | 196 | 89.46 | TG, mg/dL |
| Dearwater et al, 1986 |  | 17 | 114.8 | 84.3 | 77 | 130.8 | 61.9 | TG, mg/dL |
| Flank et al, 2014 |  | 27 | 25.2 | 14.4 | 107 | 23.4 | 14.4 | TG, mg/dl |
| Hübner-Woźniak et al, 2010 |  | 10 | 13.7 | 6.3 | 10 | 18.4 | 8.3 | TG, mg/dl |
| Sadowska-Krepa et al, 2015 |  | 15 | 121.7 | 47.7 | 9 | 112.4 | 27.9 | TG, mg/dL |
| Sadowska-Krepa et al, 2015 |  | 8 | 100.8 | 27.1 | 9 | 112.4 | 27.9 | TG, mg/dL |
| Sadowska-Krępa et al, 2015 |  | 15 | 61.7 | 11.8 | 9 | 59.5 | 7.9 | Catalase (UgHb− 1) |
| Sadowska-Krępa et al, 2015 |  | 8 | 60.9 | 11.1 | 9 | 59.5 | 7.9 | Catalase (UgHb− 1) |
| Hübner-Woźniak et al, 2012 |  | 14 | 0.313 | 0.032 | 10 | 0.244 | 0.058 | Catalase (UgHb-1) |
| Koury et al, 2013 |  | 13 | 0.3 | 0.6 | 9 | 1.3 | 2.7 | hsCRP, mg/dl |
| Buchholz et al, 2009 |  | 4 | 0.048 | 0.036 | 10 | 0.052 | 0.042 | hsCRP, mg/dl |
| Buchholz et al, 2009 |  | 13 | 0.012 | 0.05 | 6 | 0.05 | 0.046 | hsCRP, mg/dl |
| Schreiber et al, 2014^2^ |  | 23 | 0.17 | 0.77 | 19 | 0.74 | 0.86 | hsCRP, mg/dl |
| Bell et al, 2011^1^ |  | 55 | 0.56 | 0.1 | 50 | 0.62 | 0.11 | CIMT,mm |
| Schreiber et al, 2018^2^ |  | 25 | 0.56 | 0.11 | 16 | 0.69 | 0.1 | CIMT,mm |
| de Rossi et al, 2014^2^ |  | 29 | 109.9 | 17.8 | 29 | 106.7 | 15.1 | SBP, mmHg |
| Currie et al, 2017^2^ |  | 8 | 96 | 16 | 9 | 94 | 15 | SBP, mmHg |
| Huonker et al, 1998 |  | 29 | 129.8 | 14.1 | 20 | 128.5 | 13.9 | SBP, mmHg |
| Buchholz et al, 2009 |  | 9 | 108.9 | 26.2 | 17 | 99.4 | 12.3 | SBP, mmHg |
| Buchholz et al, 2009 |  | 19 | 117.6 | 18.2 | 11 | 135.6 | 29.8 | SBP, mmHg |
| Bell et al, 2011^1^ |  | 55 | 125 | 15.4 | 50 | 129 | 21.3 | SBP, mmHg |
| Currie et al, 2015 |  | 13 | 108 | 21 | 8 | 109 | 24 | SBP, mmHg |
| Paim et al, 2019^2^ |  | 23 | 105 | 16 | 17 | 106 | 15 | SBP, mmHg |
| Flank et al, 2014 |  | 27 | 121.6 | 11.8 | 107 | 131.7 | 24.6 | SBP, mmHg |
| de Rossi et al, 2014^2^ |  | 29 | 68.5 | 12.9 | 29 | 65.9 | 11.3 | DBP, mmHg |
| Currie et al, 2017^2^ |  | 8 | 48 | 15 | 9 | 55 | 6 | DBP, mmHg |
| Huonker et al, 1998 |  | 29 | 85 | 11.9 | 20 | 84.2 | 15.6 | DBP, mmHg |
| Buchholz et al, 2009 |  | 9 | 70.2 | 11.1 | 17 | 67.5 | 10.2 | DBP, mmHg |
| Buchholz et al, 2009 |  | 19 | 77.4 | 12.5 | 11 | 82.9 | 15.6 | DBP, mmHg |
| Currie et al, 2015 |  | 13 | 65 | 14 | 8 | 64 | 15 | DBP, mmHg |
| Paim et al, 2019^2^ |  | 23 | 67 | 13 | 17 | 68 | 9 | DBP, mmHg |
| Flank et al, 2014 |  | 27 | 71.9 | 9.2 | 107 | 79.3 | 12 | DBP, mmHg |
| Maggioni et al, 2012 |  | 10 | 67 | 7 | 10 | 77 | 10 | HR resting, b.p.m |
| Huonker et al, 1998 |  | 29 | 68.4 | 13.5 | 20 | 77.6 | 9.8 | HR resting, b.p.m |
| de Rossi et al, 2014^2^ |  | 29 | 68.8 | 12.9 | 29 | 76.5 | 11.3 | HR resting, b.p.m |
| Currie et al, 2017 |  | 8 | 67 | 8 | 9 | 80 | 14 | HR resting, b.p.m |
| Currie et al, 2015 |  | 13 | 67 | 10 | 8 | 72 | 11 | HR resting, b.p.m |
| Lovell et al, 2012 |  | 10 | 160 | 14 | 10 | 144 | 20 | HR resting, b.p.m |
| Paim et al, 2019^2^ |  | 23 | 67 | 12 | 17 | 74 | 11 | HR resting, b.p.m |
| de Rossi et al, 2014^2^ |  | 14 | 30.3 | 2.62 | 14 | 29.6 | 3.37 | Aortic root diameter, mm |
| de Rossi et al, 2014^2^ |  | 15 | 31.8 | 3.48 | 15 | 30.4 | 3.48 | Aortic root diameter, mm |
| Currie et al, 2017^2^ |  | 8 | 22 | 19 | 9 | 22 | 8 | Aortic root diameter, mm |
| Maggioni et al, 2012 |  | 10 | 46.6 | 5.1 | 7 | 41.4 | 5.3 | LV end diastolic diameter,mm |
| de Rossi et al, 2014^2^ |  | 14 | 45.8 | 3.37 | 14 | 44.2 | 3.74 | LV end diastolic diameter,mm |
| de Rossi et al, 2014^2^ |  | 15 | 49.4 | 4.64 | 15 | 45 | 3.87 | LV end diastolic diameter,mm |
| Currie et al, 2017^2^ |  | 8 | 8 | 0.8 | 9 | 9.2 | 1.3 | Posterior wall thickness, mm |
| de Rossi et al, 2014 |  | 14 | 8.1 | 0.75 | 14 | 8.3 | 0.37 | Posterior wall thickness, mm |
| de Rossi et al, 2014 |  | 15 | 7.9 | 0.77 | 15 | 7.7 | 0.77 | Posterior wall thickness, mm |
| Maggioni et al, 2012 |  | 10 | 9.8 | 1.2 | 7 | 8.4 | 1.1 | Posterior wall thickness, mm |
| Currie et al, 2017^2^ |  | 8 | 9.7 | 0.8 | 9 | 10.4 | 1.7 | Septal wall thickness, mm |
| Schumacher et al, 2009^2^ |  | 25 | 9.5 | 1 | 10 | 10.6 | 0.9 | Septal wall thickness, mm |
| de Rossi et al, 2014^2^ |  | 14 | 8.2 | 0.75 | 14 | 8.5 | 0.75 | Septal wall thickness, mm |
| de Rossi et al, 2014^2^ |  | 15 | 8.2 | 0.77 | 15 | 8.3 | 1.16 | Septal wall thickness, mm |
| Maggioni et al, 2012 |  | 10 | 10.2 | 1.1 | 7 | 8.6 | 0.8 | Septal wall thickness, mm |
| de Rossi et al, 2014^2^ |  | 14 | 89.6 | 16.46 | 14 | 79.4 | 11.97 | LV mass index, g m-2 |
| de Rossi et al, 2014^2^ |  | 15 | 81.5 | 13.93 | 15 | 76.7 | 16.64 | LV mass index, g m-2 |
| Maggioni et al, 2012 |  | 10 | 83.1 | 15.7 | 7 | 56.3 | 17.5 | LV mass index, g m-2 |
| Currie et al, 2017^2^ |  | 8 | 74.6 | 6.6 | 9 | 86.1 | 10 | LV mass index, g m-2 |
| Currie et al, 2017^2^ |  | 8 | 105 | 10 | 9 | 104 | 21 | End diastolic volume, mL |
| Maggioni et al, 2012 |  | 10 | 72.6 | 21.7 | 7 | 80.4 | 18.7 | End diastolic volume, mL |
| Currie et al, 2017^2^ |  | 8 | 63 | 9 | 9 | 59 | 9 | SV, mL |
| Huonker et al, 1998 |  | 29 | 79.1 | 15.3 | 20 | 63.3 | 15.1 | SV, mL |
| de Rossi et al, 2014^2^ |  | 14 | 77 | 16.83 | 14 | 62.8 | 11.97 | SV, mL |
| de Rossi et al, 2014^2^ |  | 15 | 63.9 | 12.38 | 15 | 59.6 | 13.16 | SV, mL |
| Currie et al, 2017^2^ |  | 8 | 3.6 | 1.3 | 9 | 3.9 | 1.1 | Q, L/min |
| de Rossi et al, 2014^2^ |  | 14 | 5.1 | 1.12 | 14 | 5.2 | 1.12 | Q, L/min |
| de Rossi et al, 2014^2^ |  | 15 | 4.5 | 1.55 | 15 | 4.2 | 1.55 | Q, L/min |
| Currie et al, 2017^2^ |  | 8 | 60 | 7 | 9 | 57 | 7 | EF, % |
| Huonker et al, 1998 |  | 29 | 60.3 | 8.8 | 20 | 59.9 | 5.6 | EF, % |
| de Rossi et al, 2014^2^ |  | 14 | 66.4 | 4.49 | 14 | 67.6 | 4.49 | EF, % |
| de Rossi et al, 2014^2^ |  | 15 | 65.7 | 3.48 | 15 | 67.7 | 5.03 | EF, % |
| Maggioni et al, 2012 |  | 10 | 60.1 | 8.8 | 7 | 61.4 | 4.6 | EF, % |
| Currie et al, 2017^2^ |  | 8 | 2.07 | 0.42 | 9 | 1.55 | 0.28 | E/A ratio |
| de Rossi et al, 2014^2^ |  | 14 | 1.54 | 0.34 | 14 | 1.48 | 0.49 | E/A ratio |
| de Rossi et al, 2014^2^ |  | 15 | 1.72 | 0.46 | 15 | 1.58 | 0.46 | E/A ratio |
| Maggioni et al, 2012 |  | 10 | 1.49 | 0.3 | 7 | 1.64 | 0.8 | E/A ratio |
| Currie et al, 2017^2^ |  | 8 | 88 | 11 | 9 | 101 | 7 | Isovolumic relaxation time, ms |
| Maggioni et al, 2012 |  | 10 | 100.6 | 15.7 | 7 | 107.9 | 17.7 | Isovolumic relaxation time, ms |
| Huonker et al, 1998 |  | 29 | 34.5 | 4.3 | 20 | 23.9 | 3.8 | Peak oxygen uptake, ml/kg/min |
| Ingles et al, 2016^2^ |  | 9 | 21.94 | 11.73 | 7 | 20.29 | 6.02 | Peak oxygen uptake, ml/kg/min |
| Maggioni et al, 2012 |  | 10 | 21.8 | 4.8 | 7 | 13.3 | 3.3 | Peak oxygen uptake, ml/kg/min |
| Dallmeijer et al, 1997^2^ |  | 11 | 14.4 | 4.5 | 13 | 9.1 | 2.5 | Peak oxygen uptake, ml/kg/min |
| Davis et al, 1988 |  | 15 | 22.4 | 5.42 | 15 | 15.6 | 3.49 | Peak oxygen uptake, ml/kg/min |
| Zwiren et al, 1975 |  | 11 | 35 | 7.6 | 9 | 19.6 | 5.5 | Peak oxygen uptake, ml/kg/min |
| Tanhoffer et al, 2014 |  | 6 | 21 | 3 | 7 | 15 | 4 | Peak oxygen uptake, ml/kg/min |
| Lee et al, 2015 |  | 24 | 27.43 | 6.6 | 6 | 18.38 | 3.96 | Peak oxygen uptake, ml/kg/min |
| Bhambhani et al, 1995 |  | 8 | 11.4 | 2.5 | 8 | 9 | 2.5 | Peak oxygen uptake, ml/kg/min |
| Lovell et al, 2012 |  | 10 | 40.4 | 5.5 | 10 | 21.23 | 4.7 | Peak oxygen uptake, ml/kg/min |
| Wecht et al, 2006 |  | 9 | 2.23 | 0.27 | 9 | 1.48 | 0.44 | Peak oxygen uptake, L/min |
| Lovell et al, 2012 |  | 10 | 3.17 | 0.43 | 10 | 1.7 | 0.41 | Peak oxygen uptake, L/min |
| Bhambhani et al, 1995 |  | 8 | 1.43 | 0.35 | 8 | 1.06 | 0.37 | Peak oxygen uptake, L/min |
| Davis et al, 1988 |  | 15 | 2.24 | 0.14 | 15 | 1.56 | 0.09 | Peak oxygen uptake, L/min |
| Huonker et al, 1998 |  | 29 | 183.3 | 15.2 | 20 | 161.8 | 22.9 | Peak HR, b.p.m. |
| Maggioni et al, 2012 |  | 10 | 150 | 16 | 7 | 140 | 19 | Peak HR, b.p.m. |
| Zwiren et al, 1975 |  | 11 | 182.7 | 14 | 9 | 174.1 | 15.3 | Peak HR, b.p.m. |
| Wecht et al. 2006 |  | 9 | 161 | 11 | 9 | 157 | 16 | Peak HR, b.p.m. |
| Davis et al, 1988 |  | 15 | 181.7 | 4 | 15 | 183 | 3 | Peak HR, b.p.m. |
| Lovell et al, 2012 |  | 10 | 184 | 11 | 10 | 172 | 12 | Peak HR, b.p.m. |
| Huonker et al, 1998 |  | 29 | 88.5 | 16.3 | 20 | 49.6 | 18.2 | Peak workload, W |
| Ingles et al, 2016^2^ |  | 9 | 75 | 25.62 | 7 | 21.94 | 11.73 | Peak workload, W |
| Davis et al, 1988 |  | 15 | 97.1 | 24.4 | 15 | 61.7 | 20.14 | Peak workload, W |
| Wecht et al. 2006^2^ |  | 9 | 164 | 21 | 9 | 105 | 28 | Peak workload, W |
| Lovell et al, 2012 |  | 10 | 210 | 22 | 10 | 121 | 30 | Peak workload, W |
| **Abbreviations:** HOMA-IR: Homeostatic Model Assessment for Insulin Resistance; TC: total cholesterol; HDL: High-density lipoprotein: LDL: Low density lipoprotein; TG: triglycerides; SBP: systolic blood pressure, DBP: diastolic blood pressure; HR: heart rate; hsCRP: High-sensitivity C-reactive protein; LV: Left ventricular...; E/A ratio: Early to late ventricular filling ratio  **Desirable levels of blood biomarkers**: U.S. Centre for Disease Control and Prevention (CDC) suggest the following cholesterol levels as desirable: fasting glucose <99 mg/dL, total cholesterol <200 mg/dL, LDL <100 mg/dL, HDL≥60 mg/dL, triglycerides <150 mg/dL and systolic and diastolic blood pressure <120 mmHg and < 80 mmHg respectively. The American Heart Association and CDC define the risk of cardiovascular diseases to be: low when hs-CRP < 0.1 mg/dL, moderate when hs-CRP 0.1-0.3 mg/dL and high when hs-CRP > 0.3 mg/dL.  **Cardiometabolic disease status**: ^1^Individuals with cardiometabolic diseases were included in the study;  ^2^Individuals with cardiometabolic diseases were not included in the study; if not indicated the study did not provide information on comorbidities among study sample. | | | | | | | | |

Supplemental Table 8. Risk of bias assessment of the randomized controlled trials (RCT) based on the Cochrane Collaboration’s tool (RoB 2

| **Lead author name, year of publication** | **Randomization process^1^** | **Deviations from intended interventions** | **Missing outcome data** | **Measurement of the outcome** | **Selection of the reported result** | **Overall risk of bias** |
| --- | --- | --- | --- | --- | --- | --- |
| **Akkurt et al, 2017(39)** | Some concerns | Low | Low | Low | Low | Some concerns |
| **Gorgey et al, 2016(42)** | Some concerns | Low | Low | Low | Low | Some concerns |
| **Hicks et al, 2003(43)** | Some concerns | Low | Low | Low | Low | Some concerns |
| **Kim et al, 2015(44)** | Some concerns | Low | Low | Low | Low | Some concerns |
| **Kim et al, 2019(45)** | Some concerns | Low | Low | Low | Low | Some concerns |
| **Nightingale et al, 2017(46) & 2018(54)** | Some concerns | Low | Low | Low | Low | Some concerns |
| **Ordonez et al, 2013(48)** | Low | Low | Low | Low | Low | Low |
| **Rosety-Rodriguez et al, 2014(49)** | Low | Low | Low | Low | Low | Low |
| **Totosy de Zepetnek et al, 2015(50)** | Some concerns | Low | Low | Low | Low | Some concerns |
| **Lavado et al, 2013(52)** | Some concerns | Low | Low | Low | Low | Some concerns |
| **Pelletier et al, 2015(53)** | Some concerns | Low | Low | Low | Low | Some concerns |
| The Rob 2.0 tool evaluates randomization process, deviations from intended interventions, missing outcome data, measurement of outcome and selection of reported results and providing overall risk of bias as low, high or some concerns.  Low risk of bias: The study is judged to be at low risk of bias for all domains for this result.  Some concerns: The study is judged to raise some concerns in at least one domain for this result, but not to be at high risk of bias for any domain.  High risk of bias The study is judged to be at high risk of bias in at least one domain for this result. OR The study is judged to have some concerns for multiple domains in a way that substantially lowers confidence in the result.  ^1^Some concerns arise due to the fact that the study does not provide detailed information on randomization or allocation procedure, rather it just state that the study was randomized without providing additional details. | | | | | | |

**Supplemental Table 9**. Risk of bias assessment of the non-randomized controlled trials based on the Cochrane Collaboration’s tool (ROBINS-I)

| **Lead author name, year of publication** | **Bias due to confounding** | **Bias in selection of participants into the study** | **Bias in classification of interventions** | **Bias due to deviations from intended interventions** | **Bias due to missing data** | **Bias in measurement of outcomes** | **Bias in selection of the reported result** | **Overall risk of bias** |
| --- | --- | --- | --- | --- | --- | --- | --- | --- |
| ***Davis et al, 1987(40)** | Critical | Moderate | Low | Low | Low | Low | Low | High |
| ***De Almeida et al, 2011(41)** | Critical | Serious | Low | Low | Low | Low | Low | High |
| ***Hopman et al, 1996(51)** | Critical | Moderate | Low | Low | Low | Low | Low | High |
| The ROBINS assess the confounding, participant selection, intervention measurement, departures from the intended intervention, missing data, outcome measurement and selection of the reported results. Thus, classifying the risk of bias into one of five categories low, moderate, serious and critical risk of bias or no information.  Low risk of bias (the study is comparable to a well- performed randomized trial): The study is judged to be at low risk of bias for all domains.  Moderate risk of bias (the study appears to provide sound evidence for a non-randomized study but cannot be considered comparable to a well- performed randomized trial): The study is judged to be at low or moderate risk of bias for all domains.  Serious risk of bias (the study has some important problems): The study is judged to be at serious risk of bias in at least one domain, but not at critical risk of bias in any domain.  Critical risk of bias (the study is too problematic to provide any useful evidence and should not be included in any synthesis): The study is judged to be at critical risk of bias in  at least one domain.  No information on which to base a judgement about risk of bias: There is no clear indication that the study is at serious or critical risk of bias and there is a lack of information in one or more key domains of bias (a judgement is required for this). | | | | | | | | |

**Supplemental table 10.** Risk of bias assessment of observational studies using Ottawa scale (NOS)

| **Author, publication year** | **Selection (Maximum 5 stars)** | **Comparability (Maximum 2 stars)** | **Outcome (Maximum 3 stars)** | **Overall quality** |
| --- | --- | --- | --- | --- |
| Bell et al, 2011 | ** | ** | *** | Fair |
| Bhambhani et al, 1995 | ** | * | *** | Fair |
| Brenes et al, 1986 | ** | ** | *** | Fair |
| Buchholz et al, 2009 | **** | * | *** | Good |
| Currie et al, 2015 | ** | * | *** | Fair |
| Currie et al, 2017 | ** | ** | *** | Fair |
| Dallmeijer et al, 1997^a^ | ** | * | *** | Fair |
| Dallmeijer et al, 1997^b^ | ** | * | *** | Fair |
| Davis et al, 1998 | *** | * | *** | Fair |
| De Rossi et al, 2014 | ** | ** | *** | Fair |
| Dearwater et al, 1986 | ** | ** | *** | Fair |
| D'Oliveira et al, 2014 | ** | * | *** | Fair |
| Flank et al, 2014 | *** | * | *** | Fair |
| Hubli et al, 2014 | ** | ** | *** | Fair |
| Hübner-Woźniak et al, 2010 | ** | * | *** | Fair |
| Hübner-Woźniak et al, 2012 | ** | * | *** | Fair |
| Huonker et al, 1998 | ** | * | *** | Fair |
| Ingles et al, 2016 | ** | * | *** | Fair |
| Koury et al, 2013 | ** | * | *** | Fair |
| Lovell et al, 2012 | ** | * | *** | Fair |
| Maggioni et al, 2012 | ** | ** | *** | Fair |
| Nightingale et al, 2017 | ** | * | *** | Fair |
| Paim et al, 2019 | *** | * | *** | Fair |
| Rio et al, 1997 | * | ** | *** | Fair |
| Sadowska-Krępa et al, 2015 | ** | ** | *** | Fair |
| Schreiber et al, 2014 | *** | ** | *** | Good |
| Schreiber et al, 2018 | ** | ** | *** | Fair |
| Schumacher et al, 2009 | ** | ** | *** | Fair |
| Lee et al, 2015 | ** | ** | *** | Fair |
| Wecht et al. 2006 | ** | ** | *** | Fair |
| Zwiren et al, 1975 | * | * | *** | Fair |
| Tanhoffer et al, 2014 | ** | ** | *** | Fair |
| The NOS assesses each study based on three categories: selection of the study groups or participants; the comparability of the groups; and the ascertainment of either the exposure or outcome of interest. Based on parameters mentioned above and number of points obtained on NOS scale, the studies were classified as being of good quality (reaching 8-10 points), fair quality (5-7 points) and low quality (less than 5 points). | | | | |

| Supplemental Table 11. Subgroup analyses of individual factors, injury characteristics and study design factors | | | | | | | | |
| --- | --- | --- | --- | --- | --- | --- | --- | --- |
| **Study characteristics** | **Stratum** | **Number of Studies** | | **Weighted mean difference** | | **I^2^ for heterogeneity** | **Chi square for heterogeneity** | |
| **Diastolic Blood Pressure (mmHg)** | | | | | | | | |
| Individual factors | | | | | | | | |
| Age | ≤Median | 4 | | 0.010 (-3.755, 3.776) | | 0.0% | 0.29 | |
|  | ≥Median | 4 | | -3.514 (-8.955,1.926) | | 41.4% |  |  |
| Sex | All male | 5 | | 0.088 (-3.526, 3.703) | | 0.0% | 0.16 | |
|  | Mixed | 3 | | -4.095 (-10.417, 2.227) | | 52.4% |  |  |
| Weekly hours of exercise | ≤Median | 4 | | *ND | | | | |
|  | ≥Median | 3 | |  |  |  |  |  |
| Injury characteristics | | | | | | | | |
| Duration of injury | ≤Median | 4 | | *ND | | | | |
|  | ≥Median | 3 | |  |  |  |  |  |
| Injury level | Paraplegia | 1 | | 0.800 (-7.293, 8.893) | | . | | 0.55 |
|  | Tetraplegia | 4 | | -1.741 (-7.022, 3.541) | | 0.0% | |  |
|  | Both | 3 | | -2.305(-8.681, 4.071) | | 73.6% | |  |
| Study design factors | | | | | | | | |
| Location | North America | 4 | | -1.741 (-7.022, 3.541) | | 0.0% | 0.58 | |
|  | Europe | 2 | | -4.066 (-11.961, 3.828) | | 68.0% |  |  |
|  | South America | 2 | | 0.959 (-3.645, 5.564) | | 0.0% |  |  |
| Participants | ≤Median | 4 | | -1.741 (-7.022, 3.541) | | 0.0% | 0.99 | |
|  | ≥Median | 4 | | -1.746 (-6.941, 3.449) | | 65.1% |  |  |
| Study quality | High (≥8) | 2 | | -0.757( -8.694, 7.179) | | 25.4% | 0.78 | |
|  | Moderate(<8) | 6 | | -2.191 (-6.321, 1.939) | | 47.1% |  |  |
| **Systolic Blood Pressure (mmHg)** | | | | | | | | |
| Individual factors | | | | | | | | |
| Age | ≤Median | 5 | | -0.226 (-4.169, 3.718) | | 0.0% | 0.06 | |
|  | ≥Median | 4 | | -6.085 (-16.134, 3.965) | | 45.3% |  |  |
| Sex | All male | 5 | | 1.285 (-3.312, 5.882) | | 0.0% | 0.04 | |
|  | Mixed | 4 | | -6.251 (-13.714, 1.213) | | 49.9% |  |  |
| Weekly hours of exercise | ≤Median | 4 | | -6.251 (-13.714, 1.213) | | 49.9% | 0.08 | |
|  | ≥Median | 4 | | 1.278 (-4.351, 6.906) | | 0.0% |  |  |
| Injury characteristics | | | | | | | | |
| Duration of injury | ≤Median | 4 | | -0.126 (-8.143, 7.892) | | 39.0% | 0.87 | |
|  | ≥Median | 4 | | -6.071 (-10.790, -1.353) | | 6.3% |  |  |
| Injury level | Paraplegia | 2 | | -6.235 (-24.688, 12.219) | | 69.2% | 0.53 | |
|  | Tetraplegia | 3 | | 3.543 (-6.417, 13.502) | | 0.0% |  |  |
|  | Both | 3 | | -3.069 (-11.569,5.430) | | 69.5% |  |  |
| Study design factors | | | | | | | | |
| Location | North America | 5 | | -2.530 (-9.135, 4.075) | | 14.4% | 0.60 | |
|  | Europe | 2 | | -4.650 (-15.811, 6.511) | | 79.0% |  |  |
|  | South America | 2 | | 1.371 ( -5.012, 7.755) | | 0.0% |  |  |
| Participants | ≤Median | 4 | | -1.249 (-12.091, 9.594) | | 31.8% | 0.80 | |
|  | ≥Median | 5 | | -2.606 ( -7.579, 2.367) | | 50.3% |  |  |
| Study quality | High (≥8) | 2 | | -4.014 (-30.959, 22.932) | | 75.8% | 0.88 | |
|  | Moderate(<8) | 7 | | -2.370 (-6.505, 1.765) | | 29.7% |  |  |
| **Glucose (mg/dl)** | | | | | | | | |
| Individual factors | | | | | | | | |
| Age | ≤Median | 6 | | -2.980 (-5.314, -0.645) | | 0.0% | 0.60 | |
|  | ≥Median | 4 | | -4.474 (-9.435, 0.486) | | 0.0% |  |  |
| Sex | All male | 7 | | -3.165 (-5.370, -0.960) | | 0.0% | 0.80 | |
|  | Mixed | 3 | | -4.205 (-11.556, 3.147) | | 0.0% |  |  |
| Weekly hours of exercise | ≤Median | 6 | | -2.008 (-5.793, 1.778) | | 0.0% | 0.46 | |
|  | ≥Median | 4 | | -3.813 (-6.358, -1.267) | | 0.0% |  |  |
| Injury characteristics | | | | | | | | |
| Duration of injury | ≤Median | 6 | | -3.158 (-5.556, -0.760) | | 0.0% | 0.94 | |
|  | ≥Median | 3 | | -4.205 (-11.556, 3.147) | | 0.0% |  |  |
| Injury level | Paraplegia | 1 | | -10.300 (-30.006, 9.406) | | .. | 0.72 | |
|  | Tetraplegia | 5 | | -2.450 ( -6.467, 1.567) | | 0.0% |  |  |
|  | Both | 4 | | -3.448 (-5.951, -0.945) | | 0.0% |  |  |
| Study design factors | | | | | | | | |
| Location | North America | 3 | | -3.559 (-8.799, 1.682) | | 0.0% | 0.69 | |
|  | Europe | 3 | | -0.316 (-5.790, 5.159) | | 0.0% |  |  |
|  | South America | 4 | | -3.813 (-6.358, -1.267) | | 0.0% |  |  |
| Participants | ≤Median | 5 | | -2.450 (-6.467,1.567) | | 0.0% | 0.66 | |
|  | ≥Median | 5 | | -3.557 (-6.040, -1.074) | | 0.0% |  |  |
| Study quality | High (≥8) | 2 | | -5.997 (-20.634, 8.641) | | 0.0% | 0.72 | |
|  | Moderate(<8) | 8 | | -3.192 (-5.327, -1.058) | | 0.0% |  |  |
| **High density lipoprotein (HDL) (mg/dl)** | | | | | | | | |
| Individual factors | | | | | | | | |
| Age | ≤Median | 6 | | 4.873 (1.096, 8.650) | | 78.9% | 0.47 | |
|  | ≥Median | 6 | | 2.439 (-2.982, 7.860) | | 72.1% |  |  |
| Sex | All male | 7 | | 4.697 (1.126, 8.268) | | 69.4% | 0.48 | |
|  | Mixed | 5 | | 2.057 (-4.340, 8.454) | | 86.9% |  |  |
| Weekly hours of exercise | ≤Median | 4 | | 5.192 (-1.606, 11.990) | | 80.6% | 0.36 | |
|  | ≥Median | 4 | | -1.219 (-3.885, 1.447) | | 0.0% |  |  |
| Injury characteristics | | | | | | | | |
| Duration of injury | ≤Median | 6 | | 3.699 (0.133, 7.264) | | 58.7% | 0.34 | |
|  | ≥Median | 3 | | -2.161 (-4.494, 0.173) | | 0.0% |  |  |
| Injury level | Paraplegia | …. | | ….. | | …. | 0.00 | |
|  | Tetraplegia | 5 | | 7.415 (4.963, 9.868) | | 0.0% |  |  |
|  | Both | 7 | | 1.373 ( -2.750, 5.495) | | 81.5% |  |  |
| Study design factors | | | | | | | | |
| Location | North America | 5 | | 5.478 (0.883, 10.073) | | 68.0% | 0.17 | |
|  | Europe | 5 | | 4.854 (-0.330,10.038) | | 77.4% |  |  |
|  | South America | 2 | | -0.601 (-3.447, 2.245) | | 0.0% |  |  |
| Participants | ≤Median | 4 | | 6.670 (1.768, 11.573) | | 40.3% | 0.35 | |
|  | ≥Median | 8 | | 2.907 ( -0.865, 6.678) | | 83.3% |  |  |
| Study quality | High (≥8) | 2 | | -5.639 (-13.251, 1.972) | | 0.0% | 0.08 | |
|  | Moderate(<8) | 10 | | 4.845 (1.531, 8.160) | | 81.3% |  |  |
| **Low density lipoprotein (LDL) (mg/dl)** | | | | | | | | |
| Individual factors | | | | | | | | |
| Age | ≤Median | 5 | | -10.814 (-18.843,-2.784) | | 0.0% | 0.02 | |
|  | ≥Median | 4 | | 3.178 ( -1.971, 8.327) | | 0.0% |  |  |
| Sex | All male | 6 | | -10.863 (-18.635, -3.091) | | 0.0% | 0.02 | |
|  | Mixed | 3 | | 3.599 (-1.623, 8.821) | | 0.0% |  |  |
| Weekly hours of exercise | ≤Median | 4 | | -4.416 ( -16.934, 8.102) | | 45.3% | 0.53 | |
|  | ≥Median | 4 | | -8.408 (-19.402, 2.587) | | 0.0% |  |  |
| Injury characteristics | | | | | | | | |
| Duration of injury | ≤Median | 5 | | -12.706 (-21.858, -3.553) | | 0.0% | 0.26 | |
|  | ≥Median | 3 | | 3.599 (-1.623, 8.821) | | 0.0% |  |  |
| Injury level | Paraplegia | 1 | | 5.000 (-29.916, 39.916) | | … | 0.10 | |
|  | Tetraplegia | 5 | | -10.606 (-20.631, -0.580) | | 0.0% |  |  |
|  | Both | 3 | | -4.214 (-16.529, 8.100) | | 63.7% |  |  |
| Study design factors | | | | | | | | |
| Location | North America | 2 | | 3.571 (-24.790, 31.932) | | 0.0% | 0.36 | |
|  | Europe | 5 | | -4.892 (-14.745, 4.960) | | 45.1% |  |  |
|  | South America | 2 | | -10.552 (-22.571, 1.468) | | 1.3% |  |  |
| Participants | ≤Median | 4 | | -5.680 (-17.491, 6.132) | | 0.0% | 0.81 | |
|  | ≥Median | 5 | | -7.591 (-18.621, 3.438) | | 59.4% |  |  |
| Study quality | High (≥8) | 2 | | 3.571 (-24.790, 31.932) | | 0.0% | 0.55 | |
|  | Moderate(<8) | 7 | | -6.617 (-14.872,1.638) | | 46.1% |  |  |
| **Total Cholesterol(mg/dl)** | | | | | | | | |
| Individual factors | | | | | | | | |
| Age | ≤Median | 6 | | -10.306 (-16.600, -4.013) | | 7.5% | 0.03 | |
|  | ≥Median | 4 | | 1.482 (-4.160, 7.124) | | 0.0% |  |  |
| Sex | All male | 5 | | -8.524 (-14.670, -2.378) | | 0.0% | 0.61 | |
|  | Mixed | 5 | | -5.695 (-17.224, 5.834) | | 42.9% |  |  |
| Weekly hours of exercise | ≤Median | 4 | | *ND | | | | |
|  | ≥Median | 2 | |  |  |  |  |  |
| Injury characteristics | | | | | | | | |
| Duration of injury | ≤Median | 4 | | *ND | | | | |
|  | ≥Median | 3 | |  |  |  |  |  |
| Injury level | Paraplegia | 1 | | -1.100(-35.162,32.962) | | … | | 0.21 |
|  | Tetraplegia | 6 | | -8.318 (-14.395, -2.241) | | 0.0% | |  |
|  | Both | 3 | | -7.934 ( -24.354, 8.486) | | 71.4% | |  |
| Study design factors | | | | | | | | |
| Location | North America | 5 | | -15.786 (-25.502, -6.070) | | 0.0% | 0.07 | |
|  | Europe | 5 | | -2.403 (-8.243, 3.437) | | 20.8% |  |  |
|  | South America | -- | | -- | | -- |  |  |
| Participants | ≤Median | 7 | | -6.428 (-12.707, -0.148) | | 0.0% | 0.61 | |
|  | ≥Median | 3 | | -11.356 (-28.488, 5.777) | | 82.2% |  |  |
| Study quality | High (≥8) | 2 | | -0.358 (-26.468, 25.753) | | 0.0% | 0.64 | |
|  | Moderate(<8) | 8 | | -7.492 (-14.740, -0.244) | | 48.9% |  |  |
| **Triglycerides (mg/dl)** | | | | | | | | |
| Individual factors | | | | | | | | |
| Age | ≤Median | 6 | | -5.286 (-11.218 , 0.645) | | 0.0% | 0.20 | |
|  | ≥Median | 4 | | -7.988 (-33.210, 17.233) | | 56.0% |  |  |
| Sex | All male | 5 | | -9.131 (-24.521, 6.260) | | 42.3% | 0.19 | |
|  | Mixed | 5 | | 0.966 (-4.843, 6.776) | | 0.0% |  |  |
| Weekly hours of exercise | ≤Median | 4 | | *ND | | | | |
|  | ≥Median | 2 | |  |  |  |  |  |
| Injury characteristics | | | | | | | | |
| Duration of injury | ≤Median | 4 | | *ND | | | | |
|  | ≥Median | 3 | |  |  |  |  |  |
| Injury level | Paraplegia | 1 | | -2.400 (-35.601,30.801) | | …. | | 0.35 |
|  | Tetraplegia | 6 | | -7.374 (-21.024,6.276) | | 31.8% | |  |
|  | Both | 3 | | 0.944 (-4.989, 6.877) | | 0.0% | |  |
|  |  |  | |  | | | | |
| Study design factors | | | | | | | | |
| Location | North America | 5 | | -9.292 (-27.196, 8.611) | | 0.0% | 0.51 | |
|  | Europe | 5 | | -3.333 (-12.646, 5.981) | | 56.8% |  |  |
|  | South America | -- | | -- | | -- |  | |
| Participants | ≤Median | 4 | | -5.266 (-11.405, 0.873) | | 0.0% | 0.24 | |
|  | ≥Median | 6 | | -5.334 (-20.392, 9.725) | | 37.7% |  |  |
| Study quality | High (≥8) | 2 | | 1.492 (-27.111, 30.096) | | 0.0% | 0.78 | |
|  | Moderate(<8) | 8 | | -4.272 (-11.932, 3.388) | | 35.0% |  |  |
| **Relative VO_2_ (ml/kg/min)** | | | | | | | | |
| Individual factors | | | | | | | | |
| Age | ≤Median | | 5 | | 7.71 (3.83, 11.58) | 87.7% | 0.67 | |
|  | ≥Median | | 5 | | 9.33 (4.26, 14.40) | 83.6% |  |  |
| Sex | All male | | 9 | | 8.96 (5.86, 12.06) | 86.7% | 0.31 | |
|  | Mixed | | 1 | | 1.65 (-7.22,10.52) | --- |  |  |
| Weekly hours of exercise | ≤Median | | 3 | | 6.76 (3.56, 9.95) | 23.2% | 0.27 | |
|  | ≥Median | | 2 | | 6.66 (3.56, 9.76) | 39.6% |  |  |
| Injury Characteristics | | | | | | | | |
| Duration of Injury | ≤Median | | 3 | | 8.36 (3.22, 13.50) | 79.4% | 0.69 | |
|  | ≥Median | | 2 | | 5.94 (2.18, 9.70) | 12.24% |  |  |
| Injury level | Paraplegia | | 7 | | 10.515 (7.229,13.801) | 78.1% | 0.00 | |
|  | Tetraplegia | | 2 | | 3.719 (0.889, 6.549) | 53.8% |  |  |
|  | Both | | 1 | | 6.000 (2.187, 9.813) | .. |  |  |
| Study design factors | | | | | | | | |
| Location | North America | | 2 | | 4.46 (0.16, 8.77) | 77.6% | 0.15 | |
|  | South America | | 0 | | ---- | --- |  |  |
|  | Europe | | 4 | | 7.52 (4.16, 10.87) | 69.8% |  |  |
|  | Middle East | | 1 | | 15.40 (9.65, 21.15) | --- |  |  |
|  | Asia | | 1 | | 9.05 (4.93, 13.18) | --- |  |  |
|  | Australia | | 2 | | 12.53 (-0.38, 25.44) | --- |  |  |
| Participants | ≤Median | | 6 | | 8.97 (3.39, 14.55) | 90.4% | 0.78 | |
|  | ≥Median | | 4 | | 8.00 (5.37, 10.63) | 65.5% |  |  |
| Study quality | High (≥8) | | 0 | | ---- | --- | ---- | |
|  | Moderate (<8) | | 10 | | 8.52 (5.52, 11.52) | 85.5% |  |  |
| *ND-Not done due to too few studies | | | | | | | | |

**Supplemental table 12**. Grading of Recommendations Assessment, Development and Evaluation (GRADE) of all outcomes in observational studies and randomized controlled trials

| **Quality assessment** | | | | | | | **No of patients** | | **Effect** | **Quality** | **Importance** |
| --- | --- | --- | --- | --- | --- | --- | --- | --- | --- | --- | --- |
|  |  |  |  |  |  |  |  |  |  |  |  |
| **No of studies** | **Design** | **Risk of bias** | **Inconsistency** | **Indirectness** | **Imprecision** | **Other considerations (publication bias)** | **Physically active/**  **Exercise group** | **Physically inactive/**  **Control group** | **WMD (95% CI)** |  |  |
| **Glucose** | | | | | | | | | | | |
| 3 | RCTs | Serious^1^ | Not serious | Not serious | Not serious | None | 32 | 21 | -3.26 mg/dL (- 5.12; -1.39) | Moderate | Important |
| **Glucose** | | | | | | | | | | | |
| 10 | CS | Not serious | Not serious | Not serious | Not serious | None | 166 | 286 | -3.25 mg/mL (-5.36, -1.14)* | Low | Important |
| **Insulin** | | | | | | | | | | | |
| 3 | RCTs | Serious^1^ | Not serious | Not serious | Not serious | None | 32 | 21 | -3.19 µU/mL (-3.96; -2.43) | Moderate | Important |
| **Insulin** | | | | | | | | | | | |
| 7 | CS | Not serious | Not serious | Not serious | Not serious | None | 86 | 132 | -2.12 µU/mL (-4.21;-0.03) | Low | Important |
| **HOMA-IR** | | | | | | | | | | | |
| 3 | RCTs | Serious^1^ | Serious^2^ | Not serious | Not serious | None | 32 | 21 | -0.47 (-0.60; -0.35) | Low | Important |
| **HOMA-IR** | | | | | | | | | | | |
| 6 | CS | Not serious | Not serious | Not serious | Serious^4^ | None | 70 | 56 | -0.22 (-0.70, 0.26) | Very low | Important |
| **Total cholesterol** | | | | | | | | | | | |
| 4 | RCTs | Serious^1^ | Not serious | Not serious | Serious^3^ | None | 44 | 30 | -4.91 mg/dL (-12.55; 2.73) | Low | Important |
| **Total cholesterol** | | | | | | | | | | | |
| 10 | CS | Not serious | Not serious | Not serious | Not serious | None | 129 | 312 | -6.72 mg/dL (-13.09, -0.34) | Low | Important |
| **Triglycerides** | | | | | | | | | | | |
| 3 | RCTs | Serious^1^ | Serious^2^ | Not serious | Serious^3^ | None | 33 | 24 | 2.59 mg/dL (-5.84; 11.03) | Very low | Important |
| **Triglycerides** | | | | | | | | | | | |
| 10 | CS | Not serious | Not serious | Not serious | Not serious | None | 129 | 312 | -3.21 mg/dL (-9.44, 3.03) | Low | Important |
| **HDL** | | | | | | | | | | | |
| 4 | RCTs | Serious^1^ | Not serious | Not serious | Serious^3^ | None | 44 | 30 | 2.31 mg/dL (0.00; 4.61) | Low | Important |
| **HDL** | | | | | | | | | | | |
| 12 | CS | Serious^14^ | Not serious^5^ | Not serious | Not serious | None | 181 | 358 | 3.86 mg/d (0.66; 7.05) | Low | Important |
| **LDL** | | | | | | | | | | | |
| 4 | RCTs | Serious^1^ | Serious^4^ | Not serious | Serious^3^ | None | 44 | 30 | -3.79 mg/dL (-10.07; 2.50) | Low | Important |
| **LDL** | | | | | | | | | | | |
| 9 | CS | Not serious | Not serious | Not serious | Serious^4^ | None | 141 | 214 | -5.13 mg/dL (-12.2,1.91) | Very low | Important |
| **Systolic blood pressure** | | | | | | | | | | | |
| 3 | RCTs | Serious^1^ | Not serious | Not serious | Not serious | None | 46 | 30 | -0.90 mmHg (-4.32; 2.53) | Moderate | Important |
| **Systolic blood pressure** | | | | | | | | | | | |
| 9 | CS | Not serious | Not serious | Not serious | Not serious | None | 212 | 268 | -2.31 mmHg (-6.68, 2.06) | Low | Important |
| **Diastolic blood pressure** | | | | | | | | | | | |
| 3 | RCTs | Serious^1^ | Not serious | Not serious | Not serious | None | 46 | 30 | 2.32 mmHg (- 0.01; 4.47) | Moderate | Important |
| **Diastolic blood pressure** | | | | | | | | | | | |
| 8 | CS | Serious^15^ | Not serious | Not serious | Serious^4^ | None | 157 | 218 | -1.99 mmHg (-5.47; 1.50) | Very low | Important |
| **Resting heart rate** | | | | | | | | | | | |
| 7 | CS | Not serious | Not serious | Not serious | Not serious | None | 122 | 103 | -6.93 b.p.m. (-11.22, -2.65) | Low | Important |
| **Relative VO_2_ max** | | | | | | | | | | | |
| 3 | RCTs | Serious^1^ | Not serious | Not serious | Not serious | None | 33 | 26 | 4.53 mL/kg/min (3.11; 5.96) | Moderate | Important |
| **Relative VO_2_ max** | | | | | | | | | | | |
| 10 | CS | Not serious | Not serious | Not serious | Not serious | None | 133 | 102 | 8.52 mL/kg/min (5.52, 11.52) | Low | Important |
| **Absolut VO_2_ max** | | | | | | | | | | | |
| 3 | RCTs | Serious^1^ | Not serious | Not serious | Not serious | None | 33 | 32 | 0.26 L/min (0.21; 0.32) | Moderate | Important |
| **Absolut VO_2_ max** | | | | | | | | | | | |
| 4 | CS | Not serious | Serious^6^ | Not serious | Not serious | None | 42 | 42 | 0.81 L/min (0.46, 1.15) | Very low | Important |
| **Peak workload** | | | | | | | | | | | |
| 5 | CS | Not serious | Serious^8^ | Not serious | Not serious | None | 72 | 61 | 53.23 W (36.66, 69.80) | Very low | Important |
| **Peak heart rate** | | | | | | | | | | | |
| 6 | CS | Not serious | Serious^7^ | Not serious | Not serious | Serious^13^ | 84 | 70 | 8.49 b.p.m. (0.06, 16.91) | Very low | Important |
| **Catalase** | | | | | | | | | | | |
| 3 | CS | Not serious | Not serious | Not serious | Not serious | None | 37 | 28 | 0.07 **UgHb^-1^** (0.03, 0.11) | Low | Important |
| **hsCRP** | | | | | | | | | | | |
| 4 | CS | Not serious | Not Serious | Not serious | Serious^9^ | Not serious | 53 | 44 | -0.03 (-0.10, 0.04) | Very low | Important |
| **Carotid intima media thickness** | | | | | | | | | | | |
| 2 | CS | Not serious | Not serious | Not serious | Not serious | None | 80 | 66 | -0.09 mm (-0.16, -0.02) | Low | Important |
| **Aortic root diameter** | | | | | | | | | | | |
| 3 | CS | Not serious | Not serious | Not serious | Serious^9^ | None | 37 | 38 | 1.00 mm (-0.65, 2.65) | Very low | Important |
| **LV end diastolic diameter** | | | | | | | | | | | |
| 3 | CS | Not serious | Not serious | Not serious | Not serious | None | 39 | 36 | 3.24 mm (1.06, 5.43) | Low | Important |
| **Posterior wall thickness** | | | | | | | | | | | |
| 4 | CS | Not serious | Serious^10^ | Not serious | Serious^11^ | None | 47 | 45 | 0.02 mm (-0.71, 0.75) | Very low | Important |
| **Septal wall thickness** | | | | | | | | | | | |
| 5 | CS | Not serious | Serious^12^ | Not serious | Serious^11^ | None | 72 | 55 | -0.13 mm (-0.95, 0.69) | Very low | Important |
| **LV mass index** | | | | | | | | | | | |
| 4 | CS | Not serious | Serious^13^ | Not serious | Serious^11^ | None | 47 | 45 | 6.57 **g/m^2^** (-8.42, 21.56) | Very low | Important |
| **End diastolic volume** | | | | | | | | | | | |
| 2 | CS | Not serious | Not serious | Not serious | Serious^11^ | None | 18 | 16 | -2.41 mL (-14.44, 9.61) | Very low | Important |
| **Stroke volume** | | | | | | | | | | | |
| **4** | CS | Not serious | Not serious | Not serious | Not serious | None | 66 | 58 | 9.37 mL (3.07, 15.66) | Low | Important |
| **Cardiac output** | | | | | | | | | | | |
| 3 | CS | Not serious | Not serious | Not serious | Not serious | None | 37 | 38 | -0.04 **L/min** (-0.62, 0.53) | Low | Important |
| **Ejection fraction, %** | | | | | | | | | | | |
| 5 | CS | Not serious | Not serious | Not serious | Serious^11^ | None | 76 | 65 | -0.85 % (-2.66, 0.97) | Very low | Important |
| **E/A ratio** | | | | | | | | | | | |
| **4** | CS | Not serious | Not serious | Not serious | Serious^11^ | None | 47 | 45 | 0.18 (-0.07, 0.44) | Very low | Important |
| **Isovolumetric relaxation time** | | | | | | | | | | | |
| 2 | CS | Not serious | Not serious | Not serious | Not serious | None | 18 | 16 | -11.70 ms (-19.50, -3.89) | Low | Important |
| ^1^Some concerns for risk of bias in randomization process across clinical trials  ^2^I^2^ = 84.2%, p=0.002, it was not possible to explore heterogeneity due to limited number of studies included in meta-analysis  ^3^Small number of participants (<400) and wide 95% CI that could either support or refute the effectiveness of physical activity intervention  ^4^ Small number of participants (<400) and wide 95% CI that could either support or refute the beneficial role of physical activity  ^5^I^2^=79.3%, p<0.001, heterogeneity was high and there was a wide variation of point estimates across studies, however, the injury level was identified as a source of heterogeneity so we decided not to downgrade (the same logic was followed for total cholesterol and LDL)  ^6^ I^2^=85.3%, p<0.001, heterogeneity was high and there was a wide variation of point estimates across studies  ^7^ I^2^=77.9%, p<0.001, the 95% of the overall effect estimate was wide, while there was also a variation also among the point estimates of included studies  ^8^ I^2^= 78.7% , p=0.001, the 95% of the overall effect estimate was wide, while there was also a variation also among the point estimates of included studies  ^9^ Small number of participants (<400) and wide 95% CI that could either support or refute the beneficial role of physical activity  ^10^ I^2^=76.6%, p=0.005, heterogeneity was high and there was a wide variation of point estimates across studies  ^11^ Small number of participants (<400) and wide 95% CI that could either support or refute the beneficial role of physical activity  ^12^ I^2^= 82.4%, p<0.001, heterogeneity was high and there was a wide variation of point estimates across studies  ^12^ I^2^= 86.6%, <0.001, heterogeneity was high and there was a wide variation of point estimates across studies  ^13^Funnel plot was asymmetrical and Egger's p-value<0.05 suggesting a risk of publication bias  ^14^50% of studies were conducted in para-athletes, in subgroup analysis overall effect estimate were different when stratifying analysis based on activity level  ^15^50% of studies were conducted in para-athletes, in subgroup analysis overall effect estimate were different when stratifying analysis based on activity level | | | | | | | | | | | |

**Supplemental Table 13** A. Leave one out analyses (Glucose)

| Study omitted | Estimate | [95% Confidence Interval] | |
| --- | --- | --- | --- |
| de Rossi et al, 2014 | -3.5652049 | -6.1377912 | -.99261814 |
| Oliveira et al, 2014 | -3.1225469 | -5.3261642 | -.91892958 |
| Dearwater et al, 1986 | -3.2590313 | -5.5388441 | -.97921872 |
| Koury et al, 2013 | -3.0918224 | -5.3167963 | -.86684847 |
| Sadowska-Krepa et al, 2015 | -3.4782634 | -5.6326108 | -1.3239157 |
| Sadowska-Krepa et al, 2015 | -3.5026004 | -5.6679072 | -1.3372934 |
| Buchholz et al, 2009 | -3.2747037 | -5.3968611 | -1.152546 |
| Buchholz et al, 2009 | -3.1687412 | -5.293211 | -1.0442717 |
| Paim et al, 2019 | -2.836937 | -5.1735277 | -.50034624 |
| Flank et al, 2014 | -3.2276874 | -5.4082994 | -1.0470755 |
| Combined | -3.2506719 | -5.3629023 | -1.1384414 |

**Supplemental Table 13** B**.** Leave one out analyses (Insulin)

| Study omitted | Estimate | [95% Confidence Interval] | |
| --- | --- | --- | --- |
| Oliveira et al, 2014 | -1.5823537 | -3.7080355 | .54332817 |
| Dearwater et al, 1986 | -2.4133444 | -4.63029 | -.19639875 |
| Koury et al, 2013 | -1.5462191 | -3.6521082 | .55967003 |
| Sadowska-Krepa et al,2015 | -2.5852497 | -5.2673988 | .09689922 |
| Sadowska-Krepa et al,2015 | -2.7655718 | -4.9670835 | -.56406009 |
|  |  |  |  |
| Buchholz et al, 2009 | -1.7478789 | -3.8276567 | .3318989 |
| Buchholz et al, 2009 | -2.2834051 | -4.7149372 | .1481269 |
| Combined | -2.1203248 | -4.2107212 | -.02992846 |

**Supplemental Table 13** C. Leave one-out analyses (HOMA-IR)

| Study omitted | Estimate | [95% Confidence Interval] | |
| --- | --- | --- | --- |
| Oliveira et al, 2014 | -.05926976 | -.57985336 | .46131384 |
| Koury et al, 2013 | -.42603186 | -.80771208 | -.04435166 |
| Sadowska-Krepa et al,2015 | -.25057232 | -.87189758 | .37075296 |
| Sadowska-Krepa et al,2015 | -.27716577 | -.85448205 | .30015051 |
| Buchholz et al, 2009 | -.12689194 | -.54703903 | .29325518 |
| Buchholz et al, 2009 | -.22279803 | -.76382476 | .31822869 |
| Combined | -.21716482 | -.69547414 | .26114449 |

**Supplemental Table 13** D. Leave one out analyses (High Density Lipoprotein)

| Study omitted | Estimate | [95% Confidence Interval] | |
| --- | --- | --- | --- |
| Brenes et al, 1986 | 3.3918974 | .04718474 | 6.7366099 |
| Brenes et al, 1986 | 3.2706573 | .0139588 | 6.5273561 |
| Buchholz et al, 2009 | 4.1618223 | .8973034 | 7.4263411 |
| Buchholz et al, 2009 | 4.4971967 | 1.259323 | 7.7350702 |
| Dallmeijer et al, 1997 | 3.5808904 | .20011966 | 6.9616613 |
| de Rossi et al, 2014 | 4.3201599 | .81356394 | 7.826756 |
| Dearwater et al, 1986 | 3.3186564 | .04633627 | 6.5909762 |
| Flank et al, 2014 | 4.5995564 | 1.5159239 | 7.6831889 |
| Hübner-Woźniak et al, 2010 | 3.7680166 | .28589711 | 7.2501359 |
| Paim et al, 2019 | 4.3767676 | .93632895 | 7.8172064 |
| Sadowska-Krepa et al, 2015 | 3.5261848 | .17533383 | 6.8770361 |
| Sadowska-Krepa et al, 2015 | 3.4584284 | .20417361 | 6.7126832 |
| Combined | 3.8545701 | .66375122 | 7.0453889 |

**Supplemental Table 13** E. Leave one out analyses (Low Density Lipoprotein)

| Study omitted | Estimate | [95% Confidence Interval] | |
| --- | --- | --- | --- |
| Buchholz et al, 2009 | -5.7303448 | -13.339568 | 1.8788784 |
| Buchholz et al, 2009 | -6.0312834 | -13.706071 | 1.6435041 |
| Dallmeijer et al, 1997 | -5.1378565 | -12.706431 | 2.4307177 |
| de Rossi et al, 2014 | -5.6710639 | -14.063627 | 2.7214997 |
| Flank et al, 2014 | -9.855011 | -17.350538 | -2.359482 |
| Hübner-Woźniak et al, 2010 | -4.7736821 | -12.745872 | 3.1985071 |
| Paim et al, 2019 | -2.2311118 | -8.2472267 | 3.7850034 |
| Sadowska-Krepa et al, 2015 | -1.884467 | -7.6999941 | 3.9310601 |
| Sadowska-Krepa et al, 2015 | -5.8315091 | -13.651799 | 1.9887811 |
| Combined | -5.131953 | -12.173417 | 1.9095108 |

**Supplemental Table 13** F. Leave one out analyses (Total cholesterol)

| Study omitted | Estimate | [95% Confidence Interval] | |
| --- | --- | --- | --- |
| Brenes et al, 1986 | -4.2472644 | -9.617013 | 1.1224843 |
| Brenes et al, 1986 | -6.7820077 | -13.680249 | .1162341 |
| Buchholz et al, 2009 | -7.0846539 | -13.860935 | -.30837256 |
| Buchholz et al, 2009 | -7.1084361 | -13.939404 | -.27746832 |
| Dallmeijer et al, 1997 | -6.7348676 | -13.503078 | .03334241 |
| Dearwater et al, 1986 | -4.6461673 | -10.457973 | 1.1656387 |
| Flank et al, 2014 | -9.5841131 | -15.082799 | -4.0854278 |
| Hübner-Woźniak et al, 2010 | -7.1630998 | -15.520449 | 1.1942496 |
| Sadowska-Krepa et al, 2015 | -6.7148657 | -13.806274 | .37654307 |
| Sadowska-Krepa et al, 2015 | -7.4979205 | -14.057092 | -.9387486 |
| Combined | -6.7155609 | -13.088409 | -.34271237 |

**Supplemental Table 13** G. Leave one out analysis (Triglycerides)

| Study omitted | Estimate | [95% Confidence Interval] | |
| --- | --- | --- | --- |
| Brenes et al, 1986 | -3.2435653 | -10.076401 | 3.5892699 |
| Brenes et al, 1986 | -2.862541 | -9.4146957 | 3.6896138 |
| Buchholz et al, 2009 | -3.7384517 | -10.541656 | 3.0647526 |
| Buchholz et al, 2009 | -3.6858215 | -10.800125 | 3.4284818 |
| Dallmeijer et al, 1997 | -1.7584203 | -5.9575262 | 2.4406857 |
| Dearwater et al, 1986 | -3.1571016 | -9.9148273 | 3.6006238 |
| Flank et al, 2014 | -5.6790142 | -11.459667 | .10163869 |
| Hübner-Woźniak et al, 2010 | -4.4759707 | -14.704214 | 5.7522726 |
| Sadowska-Krepa et al, 2015 | -4.0171614 | -10.787697 | 2.7533741 |
| Sadowska-Krepa et al, 2015 | -2.957303 | -9.8364 | 3 .9217937 |
| Combined | -3.2056862 | -9.4367164 | 3.0253439 |

**Supplemental Table 13** H. Leave one out analysis (Diastolic blood pressure)

| Study omitted | Estimate | [95% Confidence Interval] | |
| --- | --- | --- | --- |
| Buchholz et al, 2009 | -2.5732508 | -6.2385426 | 1.092041 |
| Buchholz et al, 2009 | -1.5944935 | -5.4406223 | 2.2516356 |
| Currie et al, 2015 | -2.1339417 | -5.9005709 | 1.6326874 |
| Currie et al, 2017 | -1.5030633 | -5.2765303 | 2.2704036 |
| de Rossi et al, 2014 | -3.1250894 | -6.5599051 | .30972645 |
| Flank et al, 2014 | -.05021728 | -3.2401283 | 3.1396937 |
| Huonker et al, 1998 | -2.3221405 | -6.2012577 | 1.5569764 |
| Paim et al, 2019 | -2.0551395 | -6.1561537 | 2.0458751 |
| Combined | -1.9857298 | -5.4722573 | 1.5007978 |

**Supplemental Table 13** I. Leave one out analyses (Systolic blood pressure)

| Study omitted | Estimate | [95% Confidence Interval] | |
| --- | --- | --- | --- |
| Bell et al, 2011 | -1.8399917 | -7.1421552 | 3.4621718 |
| Buchholz et al, 2009 | -2.9447522 | -7.27846 | 1.3889558 |
| Buchholz et al, 2009 | -1.742047 | -5.9334569 | 2.4493632 |
| Currie et al, 2015 | -2.3095465 | -7.0091734 | 2.3900805 |
| Currie et al, 2017 | -2.5927551 | -7.312942 | 2.1274321 |
| de Rossi et al, 2014 | -3.3026614 | -7.9067369 | 1.3014139 |
| Flank et al, 2014 | -.49207333 | -4.2064652 | 3.2223186 |
| Huonker et al, 1998 | -2.9274738 | -7.8550425 | 2.0000954 |
| Paim et al, 2019 | -2.4075999 | -7.4381242 | 2.6229243 |
| Combined | -2.3106973 | -6.6809214 | 2.0595269 |

**Supplemental Table 13** J. Leave one out analyses (Heart rate)

| Study omitted | Estimate | [95% Confidence Interval] | |
| --- | --- | --- | --- |
| Currie et al, 2015 | -7.0836244 | -12.009086 | -2.1581638 |
| Currie et al, 2017 | -6.1523008 | -10.791577 | -1.5130243 |
| de Rossi et al, 2014 | -6.4993372 | -11.877723 | -1.1209519 |
| Huonker et al, 1998 | -6.2274604 | -11.476629 | -.97829127 |
| Lovell et al, 2012 | -8.4277906 | -11.495951 | -5.3596306 |
| Maggioni et al, 2012 | -6.1912742 | -11.251064 | -1.1314845 |
| Paim et al, 2019 | -6.6975088 | -11.92889 | -1.4661275 |
| Combined | -6.9305101 | -11.215926 | -2.6450944 |

**Supplemental Table 13** K. Leave one out analyses (Septal wall thickness)

| Study omitted | Estimate | [95% Confidence Interval] | |
| --- | --- | --- | --- |
| Currie et al, 2017 | -.01459057 | -.96092588 | .93174469 |
| Schumacher et al, 2009 | .13649754 | -.74317199 | 1.016167 |
| de Rossi et al, 2014 | -.07472984 | -1.2344985 | 1.0850389 |
| de Rossi et al, 2014 | -.13045655 | -1.2174659 | .95655274 |
| Maggioni et al, 2012 | -.51388812 | -.97939861 | -.04837766 |
| Combined | -.12737764 | -.94622474 | .69146947 |

**Supplemental Table 13** L. Leave one out analyses (Ejection fraction)

| Study omitted | Estimate | [95% Confidence Interval] | |
| --- | --- | --- | --- |
| Currie et al, 2017 | -1.1559333 | -3.0445452 | .73267871 |
| Huonker et al, 1998 | -1.1644427 | -3.1995888 | .87070328 |
| de Rossi et al, 2014 | -.69707948 | -2.8665278 | 1.472369 |
| de Rossi et al, 2014 | -.24095951 | -2.4855423 | 2.0036232 |
| Maggioni et al, 2012 | -.80788898 | -2.7021756 | 1.0863976 |
| Combined | -.84717487 | -2.6642773 | .96992756 |

**Supplemental Table 13** M. Leave one out analyses (Peak Oxygen uptake (Relative VO_2_)

| Study omitted | Estimate | [95% Confidence Interval] | |
| --- | --- | --- | --- |
| Huonker et al, 1998 | 8.2594767 | 4.8871355 | 11.631818 |
| Ingles et al, 2016 | 8.9567795 | 5.8557239 | 12.057836 |
| Maggioni et al, 2012 | 8.5296602 | 5.1976109 | 11.861711 |
| Dallmeijer et al, 1997 | 8.9251738 | 5.5669546 | 12.283392 |
| Davis et al, 1988 | 8.7366886 | 5.3473034 | 12.126073 |
| Zwiren et al, 1975 | 7.8796654 | 4.8342099 | 10.92512 |
| Tanhoffer et al, 2014 | 8.8175755 | 5.5017772 | 12.133373 |
| Lee et al, 2015 | 8.4669418 | 5.1636004 | 11.770283 |
| Bhambhani et al, 1995 | 9.3158331 | 6.4768343 | 12.154833 |
| Lovell et al, 2012 | 7.3490992 | 4.8307877 | 9.8674107 |
| Combined | 8.5200966 | 5.5173882 | 11.522805 |

Supplemental Figure 1. Metaregression of intermediate cardiovascular outcomes with age as a continuous variable

| 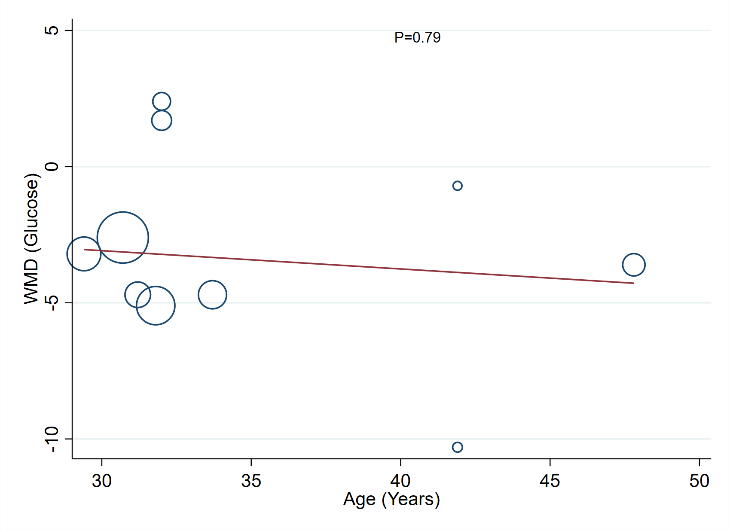Glucose | 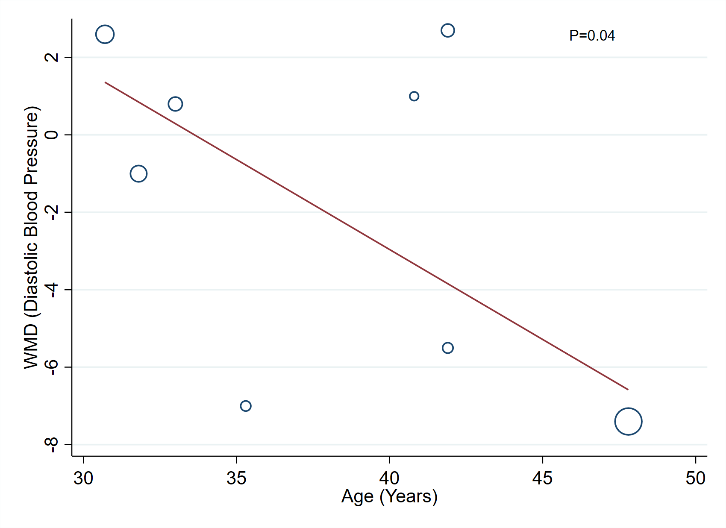Diastolic blood pressure | 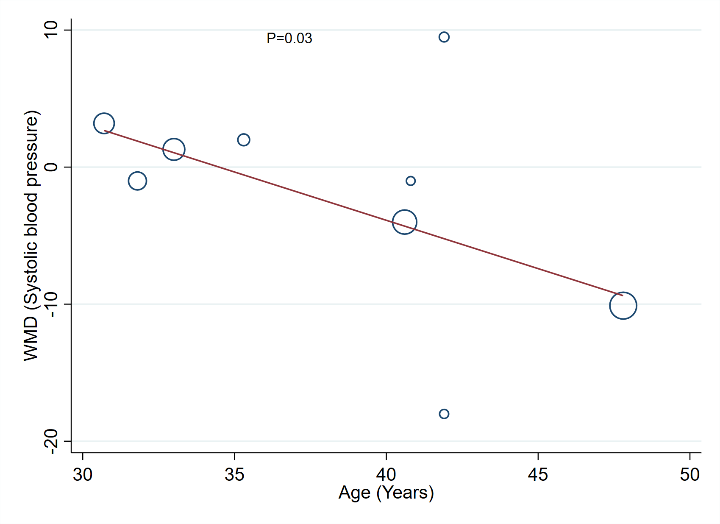Systolic blood pressure |
| --- | --- | --- |
| 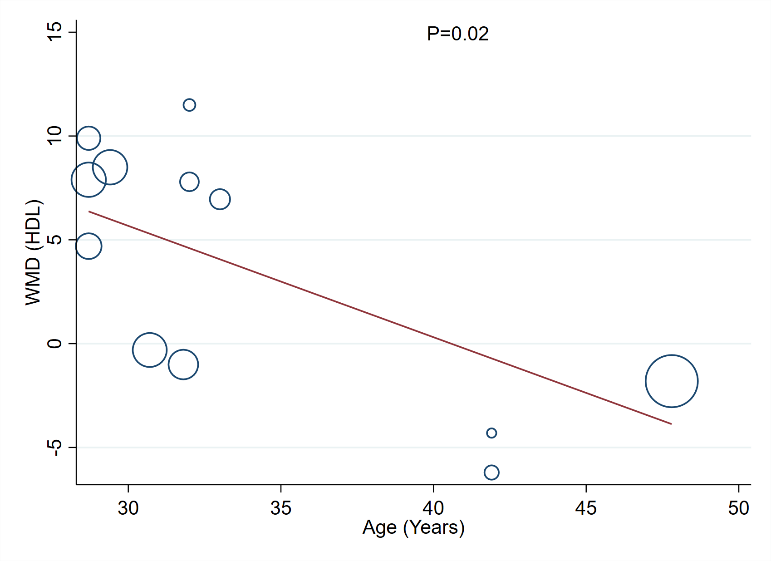High density lipoprotein | 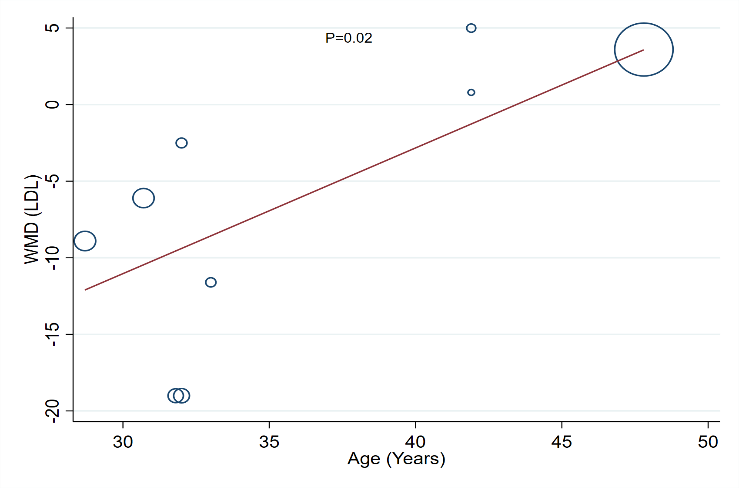Low density lipoprotein | 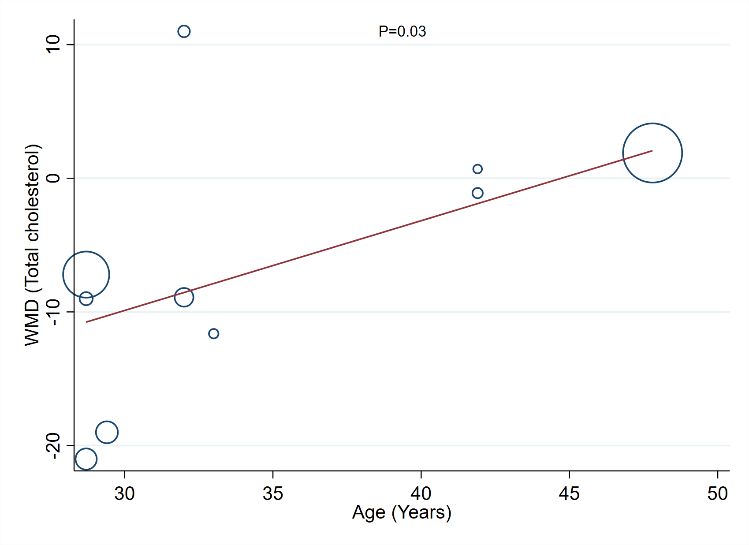Total cholesterol |
| 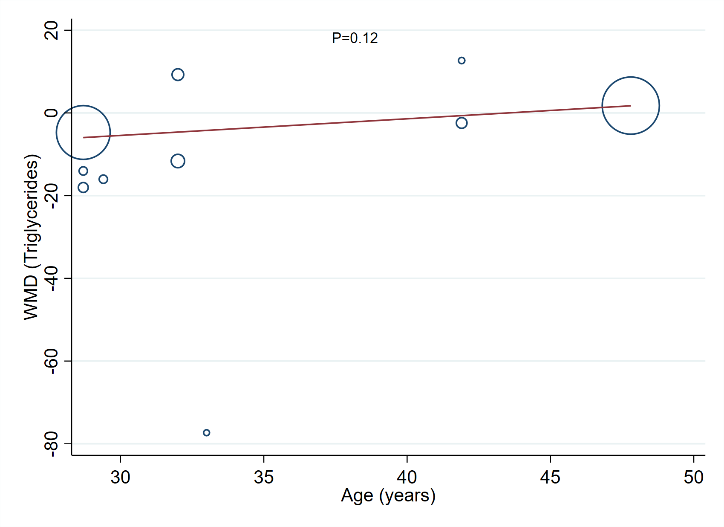Triglycerides | 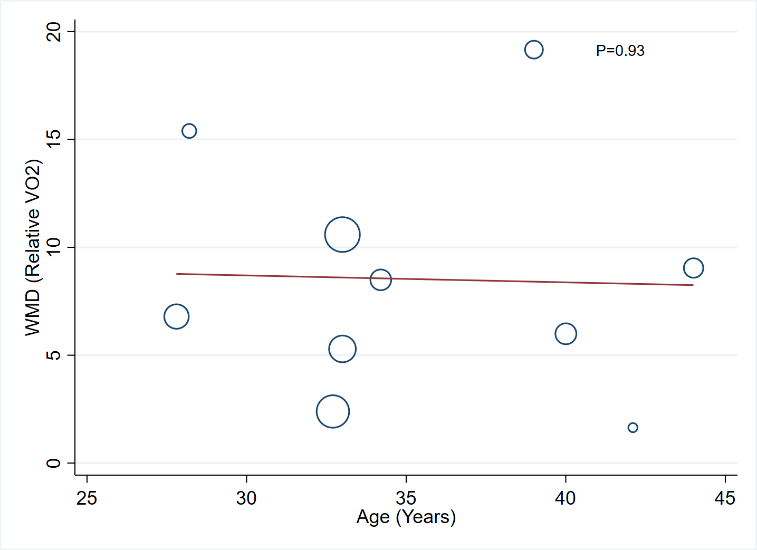  Peak Oxygen uptake (Relative VO_2_) |  |

Supplemental Figure 2. Metaregression of intermediate cardiovascular outcomes with hours of exercise per week as a continuous variable

| 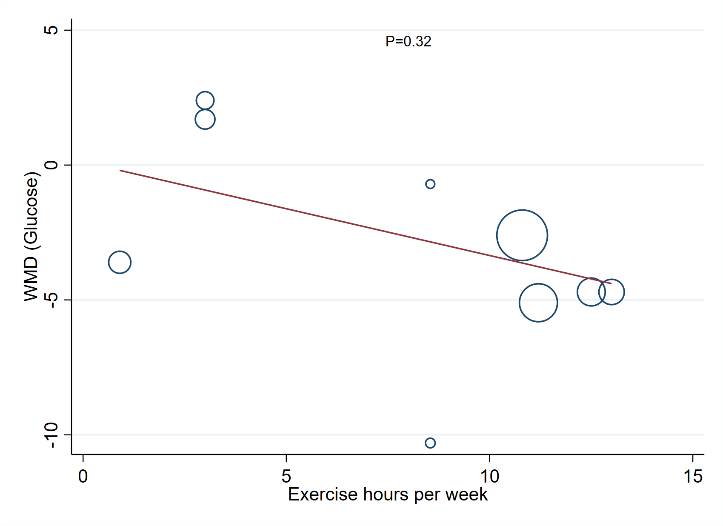Glucose | 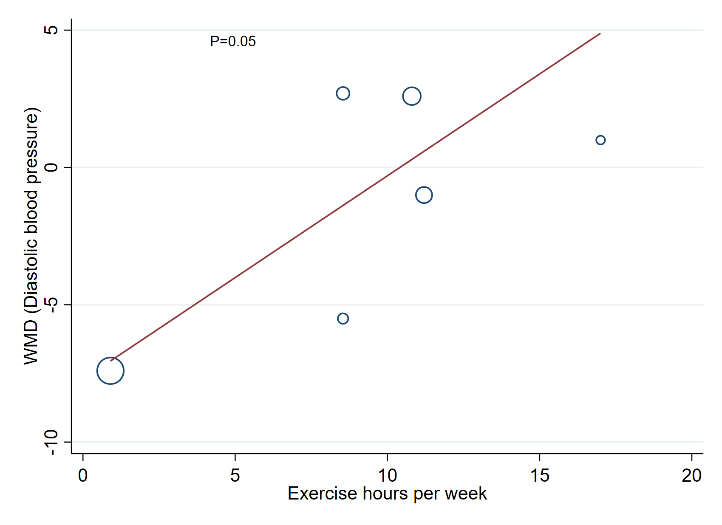Diastolic blood pressure | 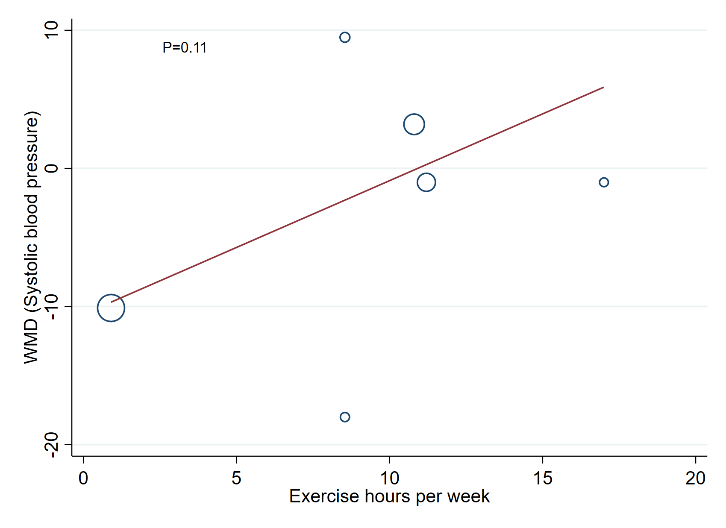Systolic blood pressure |
| --- | --- | --- |
| 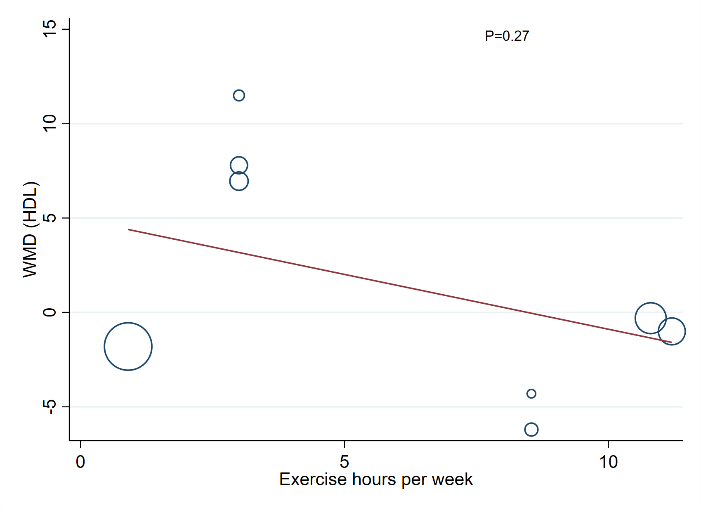High density lipoprotein | 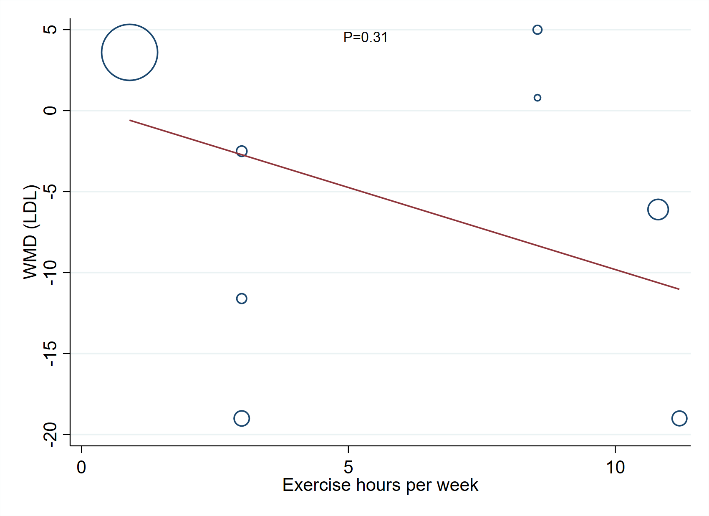Low density lipoprotein | 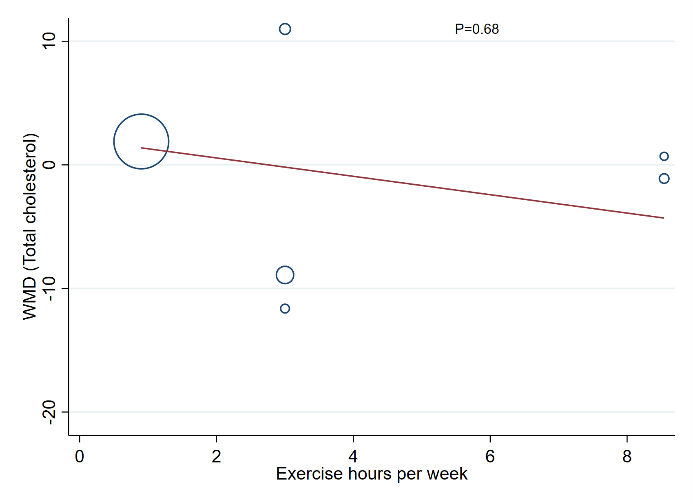Total cholesterol |
| 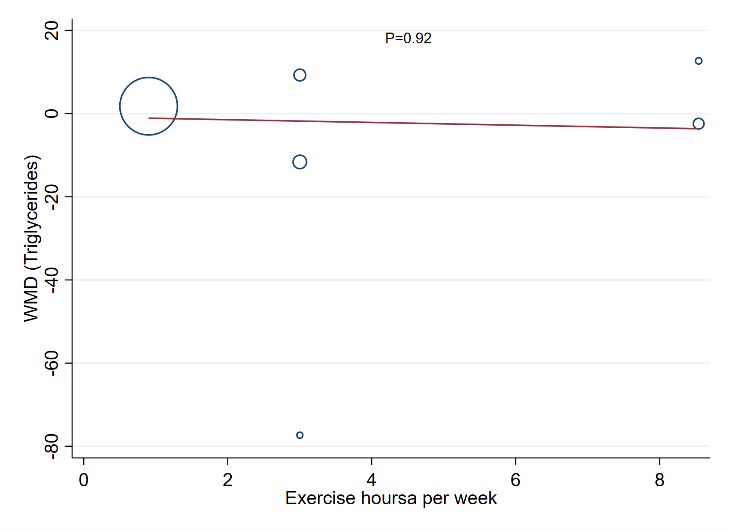Triglycerides | 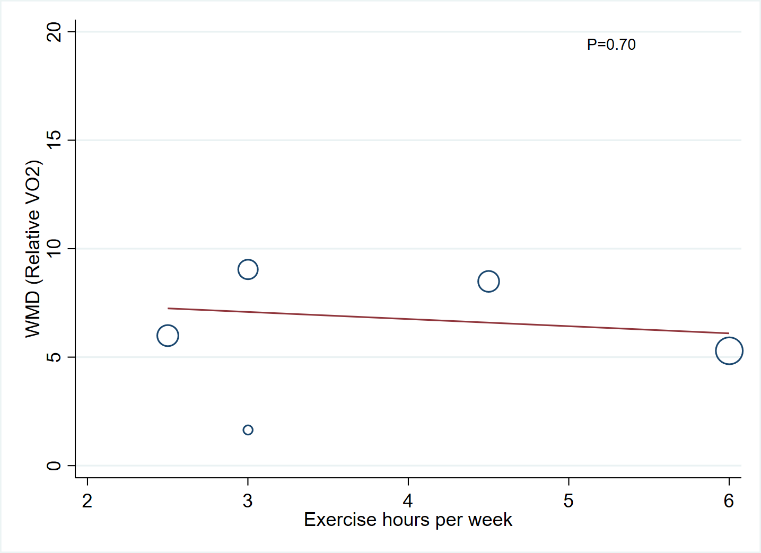  Peak Oxygen uptake (Relative VO_2_) |  |

Supplemental Figure 3. Metaregression of intermediate cardiovascular outcomes with duration of injury as a continuous variable

| 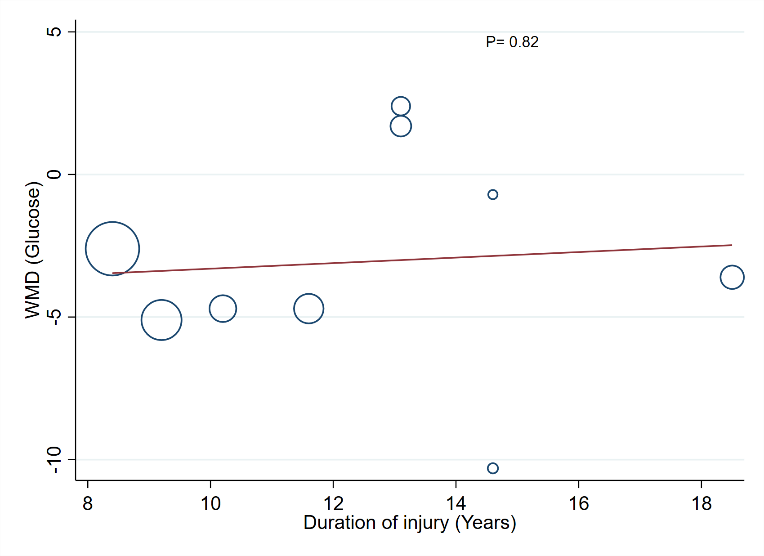Glucose | 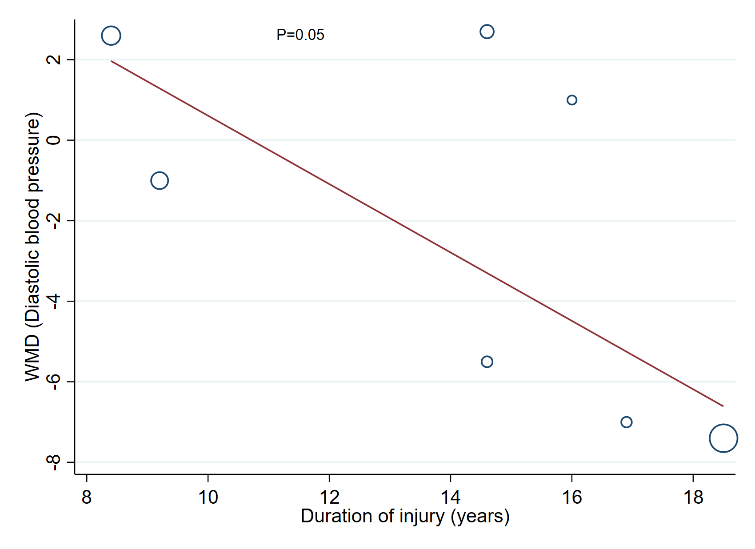Diastolic blood pressure | 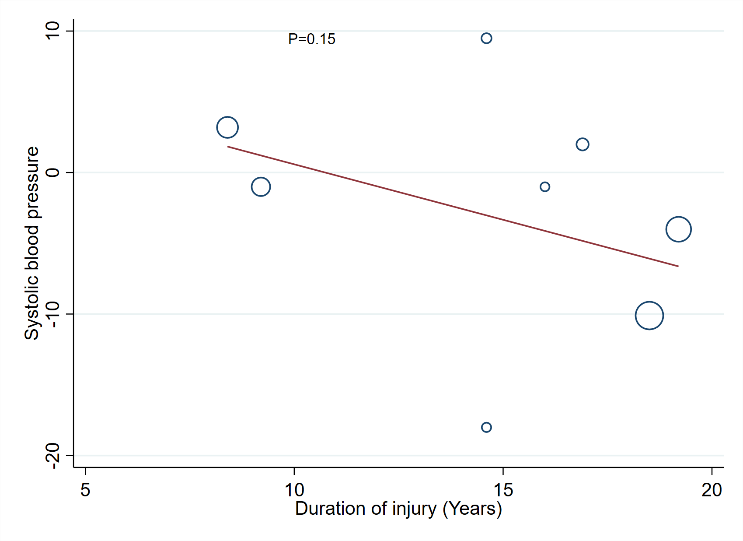Systolic blood pressure |
| --- | --- | --- |
| 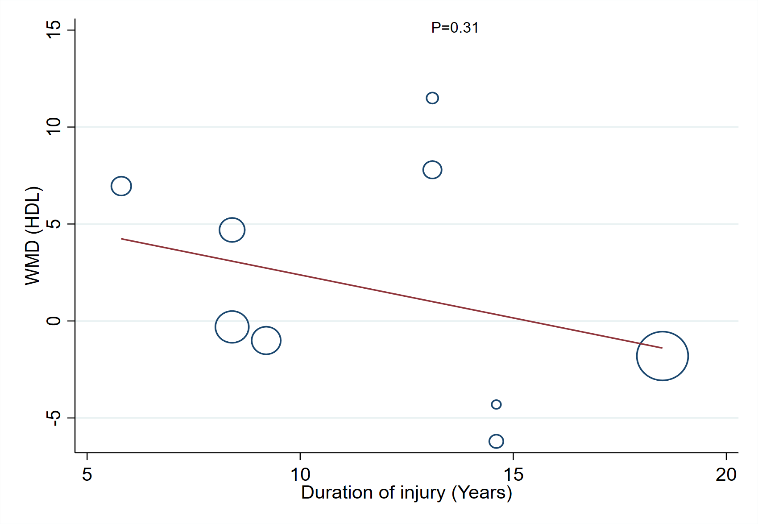High density lipoprotein | 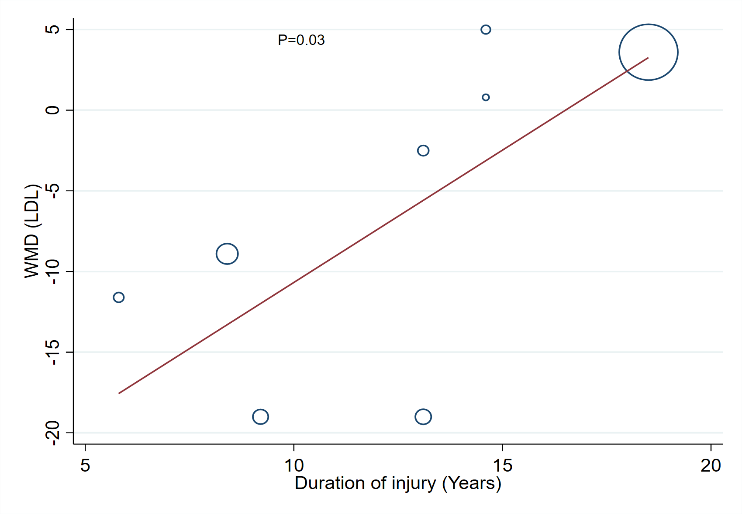Low density lipoprotein | 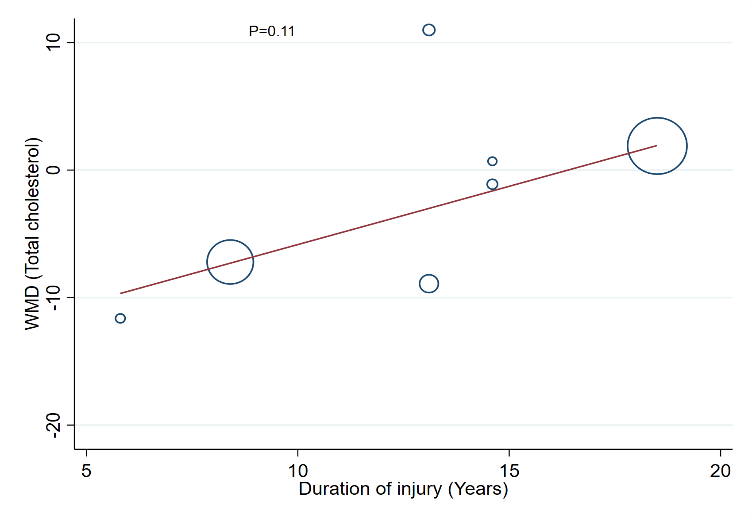Total cholesterol |
| 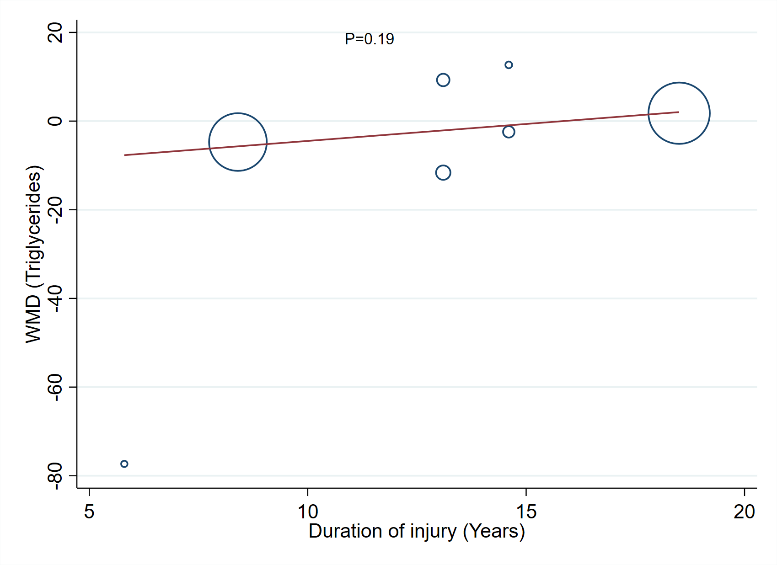Triglycerides | 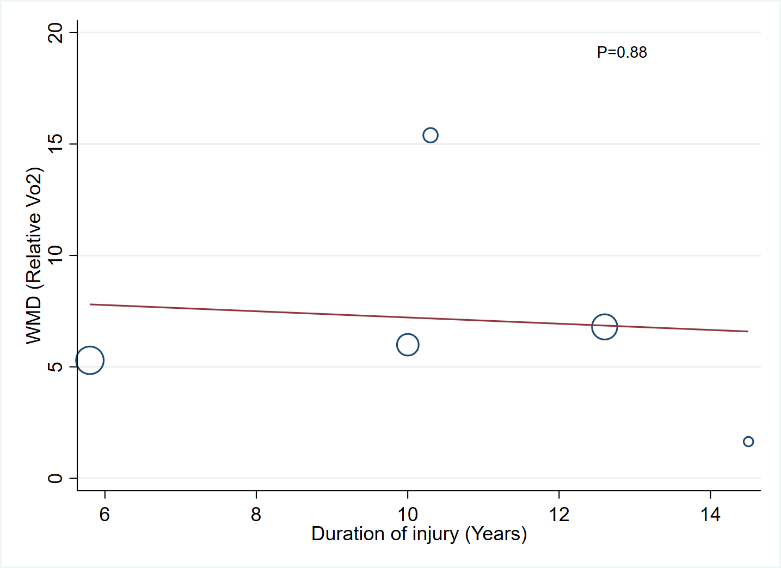  Peak Oxygen uptake (Relative VO_2_) |  |

Supplemental Figure 4. Metaregression of intermediate cardiovascular outcomes with percentage of male participants (sex) as a continuous variable

| 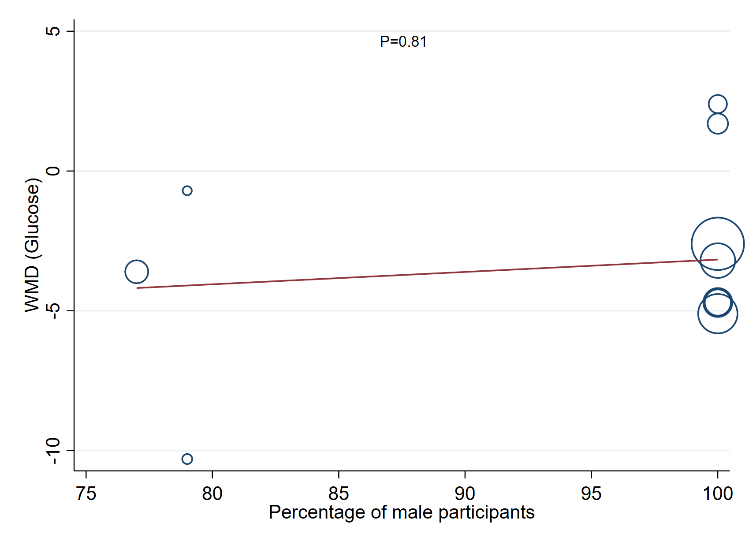Glucose | 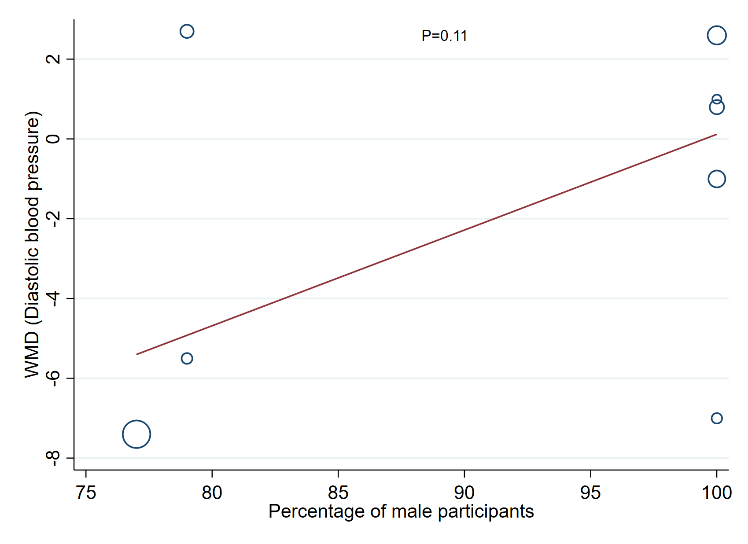Diastolic blood pressure | 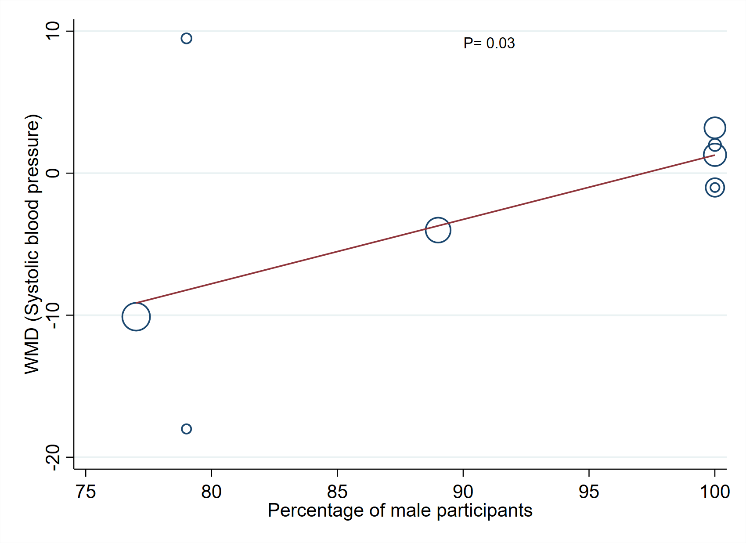Systolic blood pressure |
| --- | --- | --- |
| 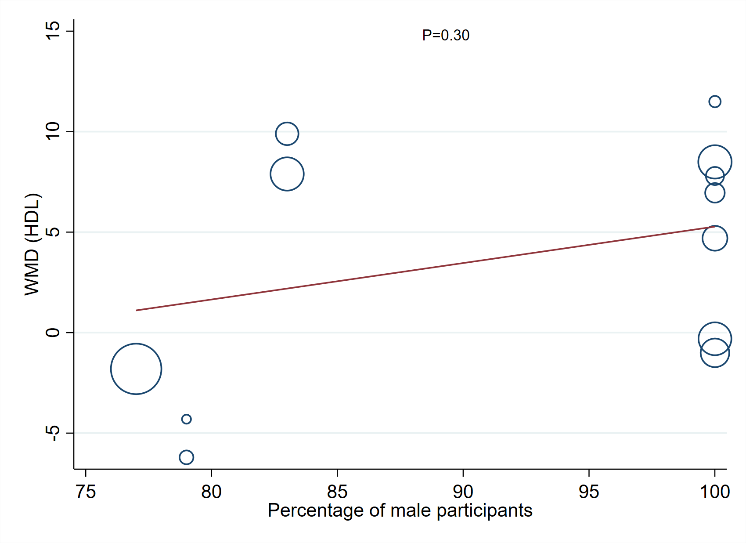High density lipoprotein | 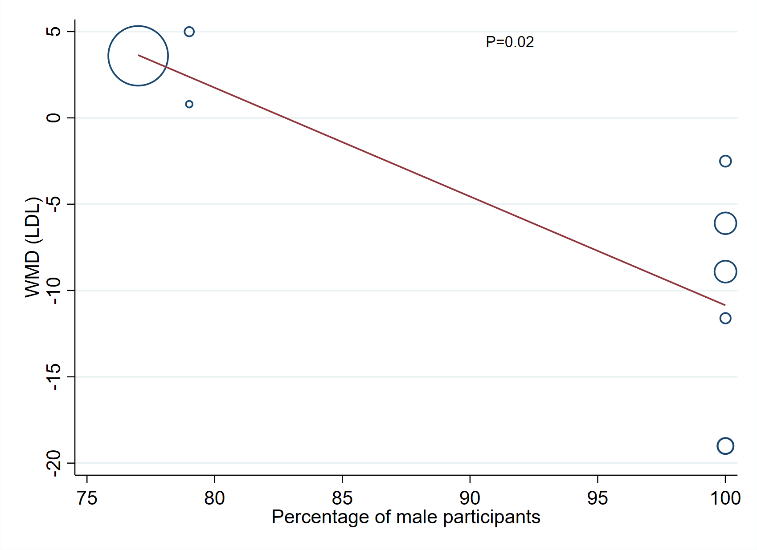Low density lipoprotein | 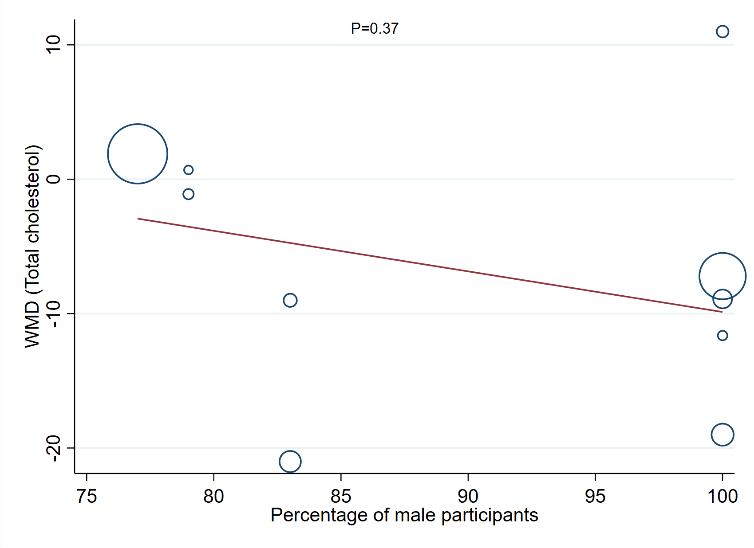Total cholesterol |
| 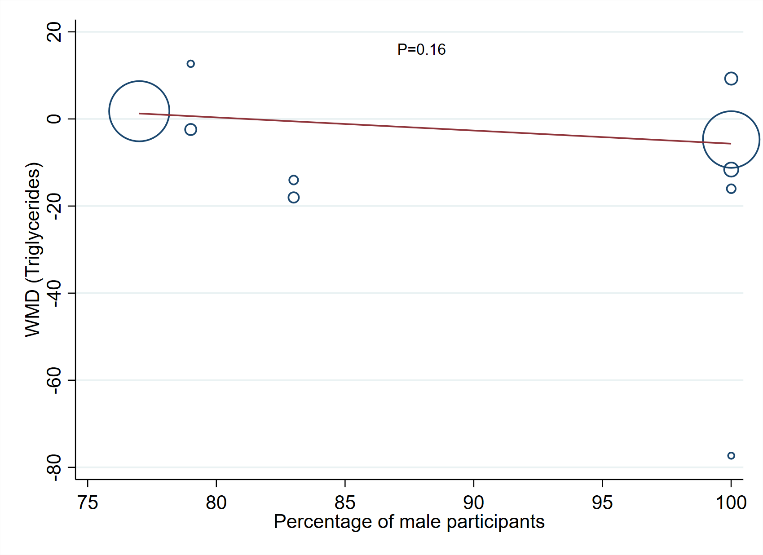Triglycerides | 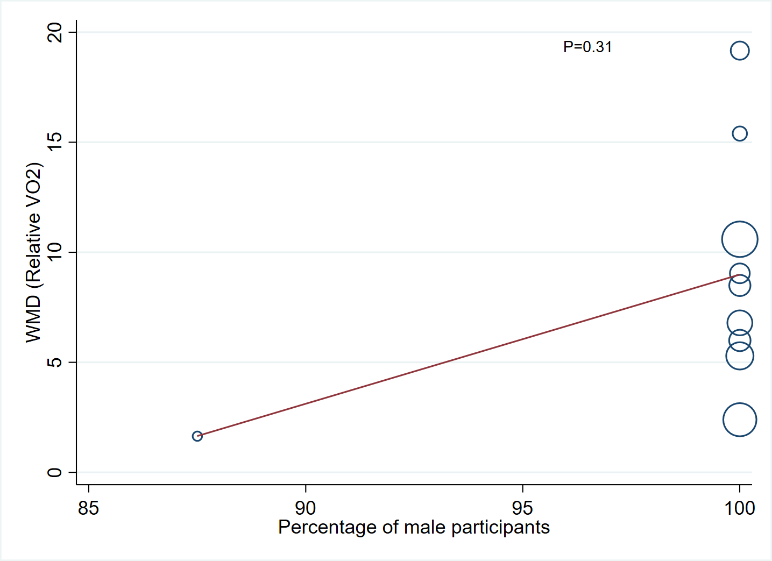  Peak Oxygen uptake (Relative VO_2_) |  |

Supplemental Figure 5. Assessment of publication bias (funnel plots and Egger’s test)

| 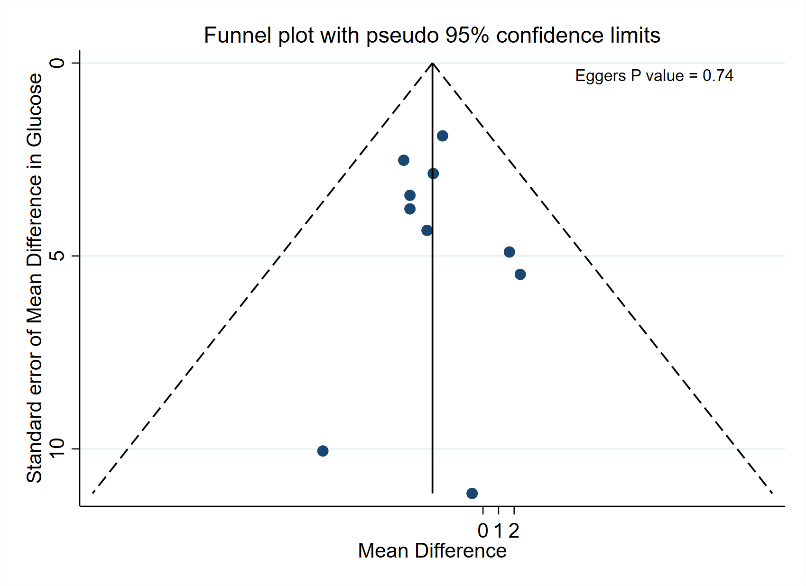Glucose | 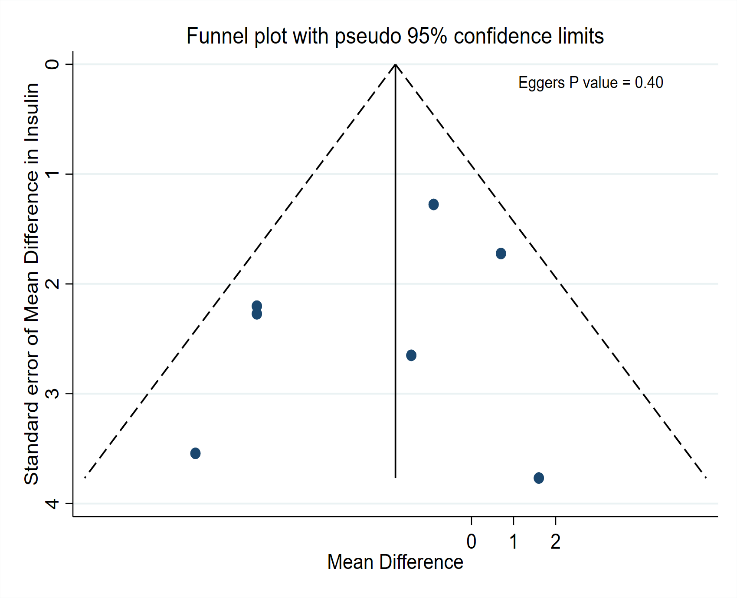Insulin | 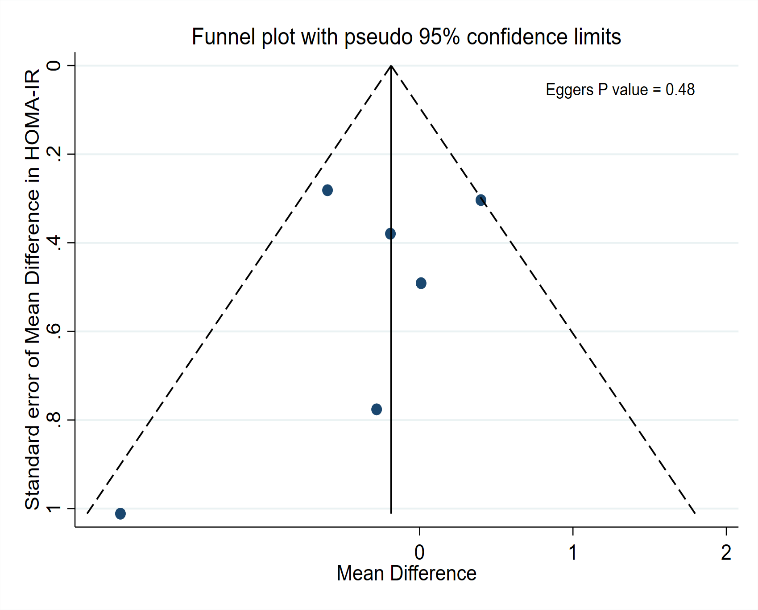HOMA-IR |
| --- | --- | --- |
| 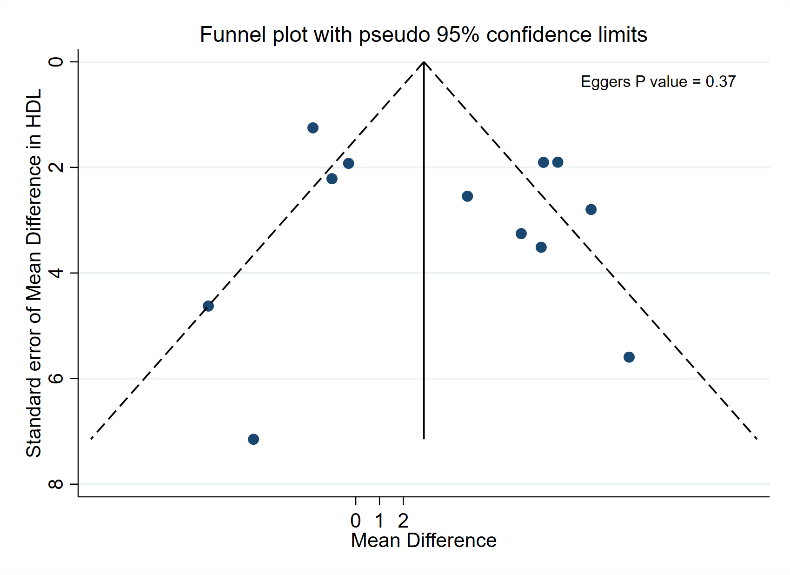High densisty lipoprotein | 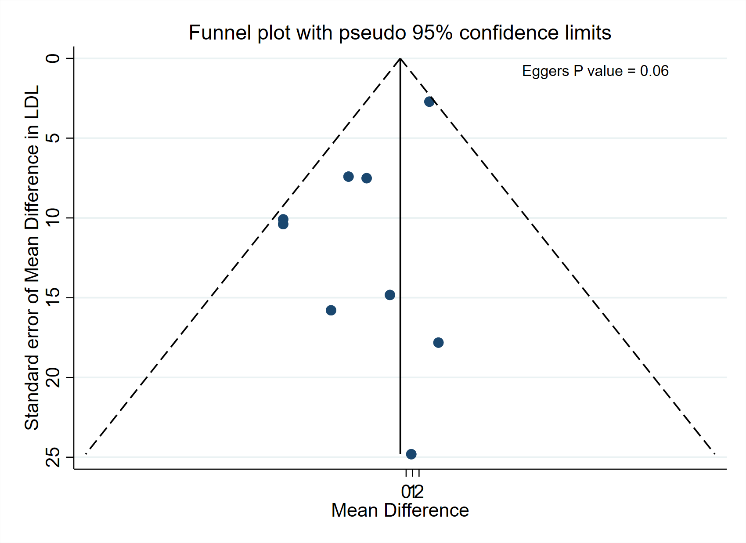Low density lipoprotein | 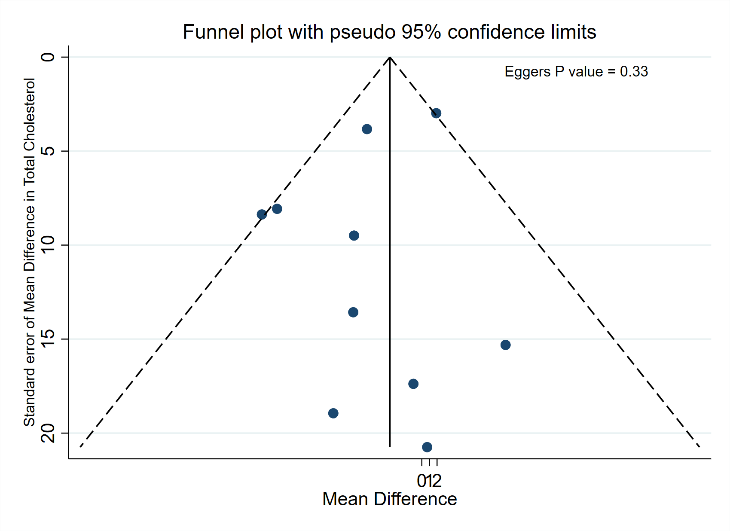Total cholesterol |
| 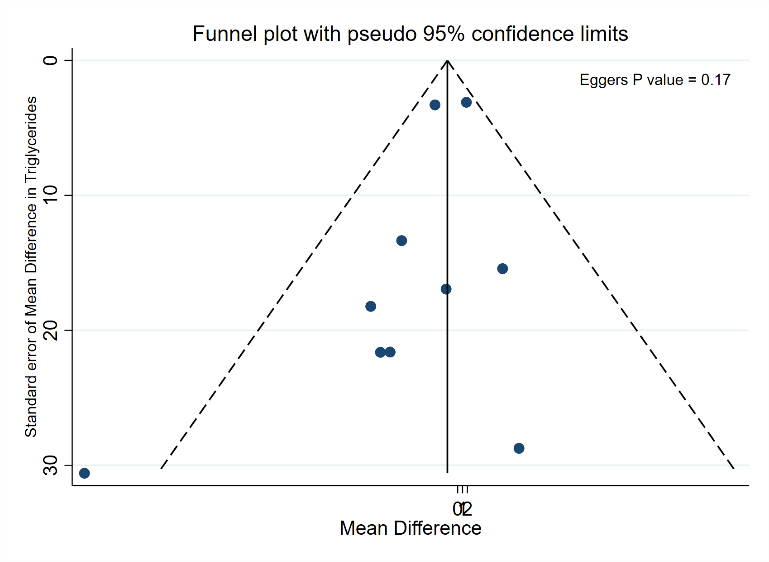Triglycerides | 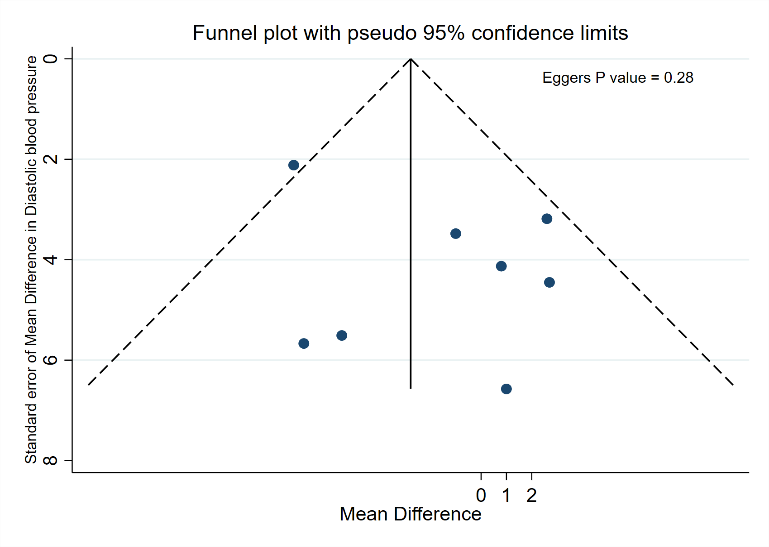Diastolic blood pressure | 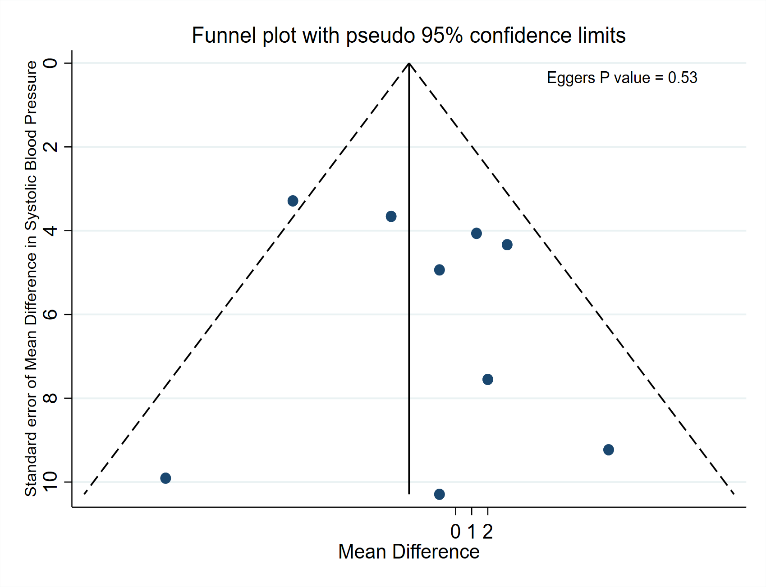Systolic blood pressure |
| 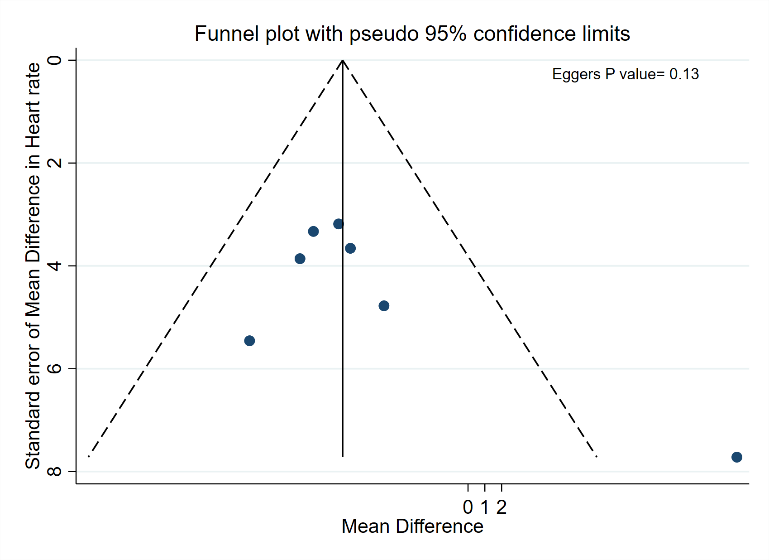Heart rate | 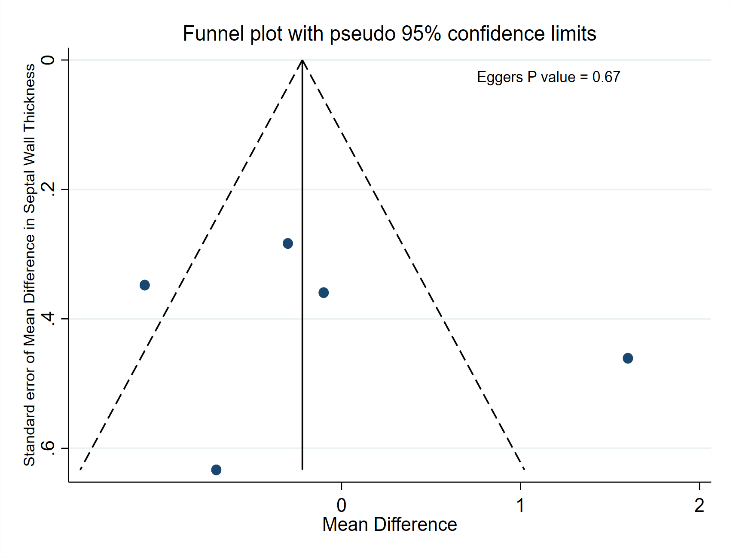Septal wall thickness | 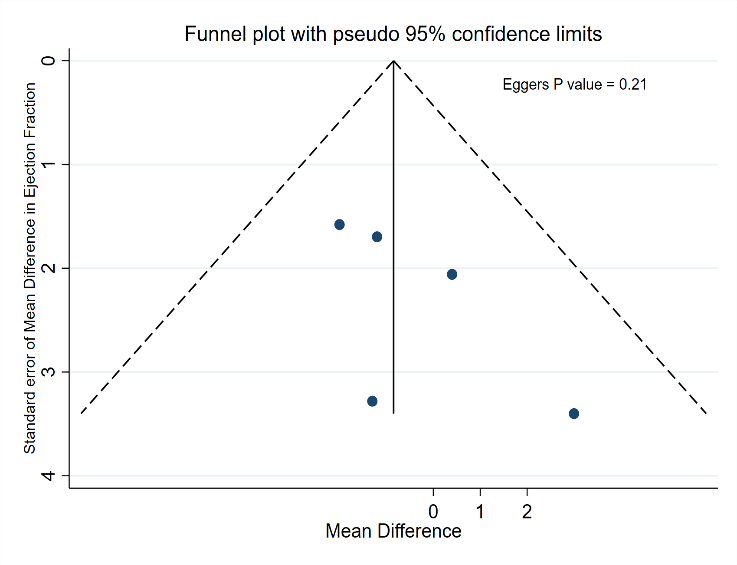Ejection fraction |
| 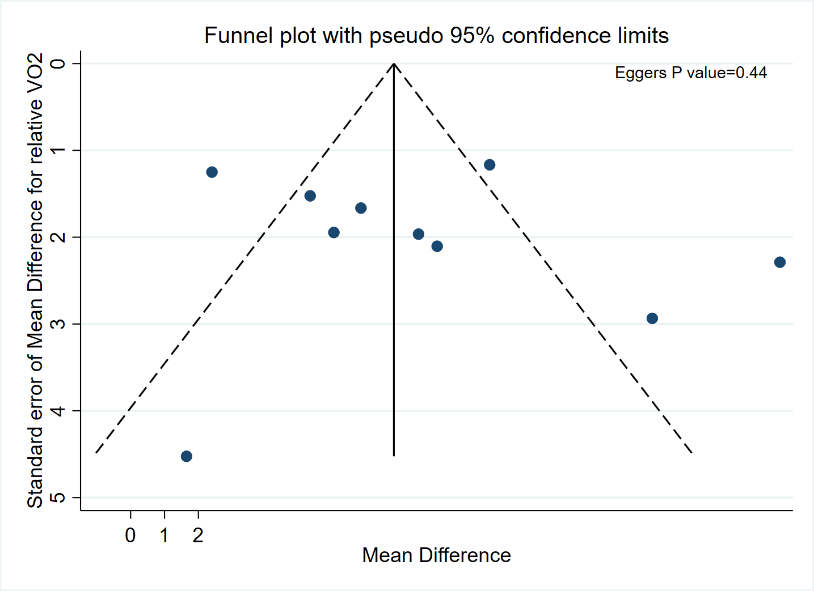  Peak Oxygen Uptake (Relative VO_2_) | 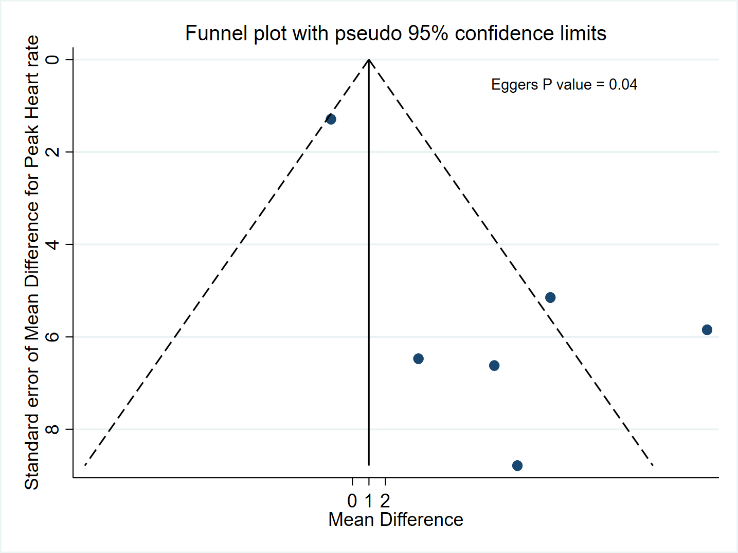  Peak heart rate | 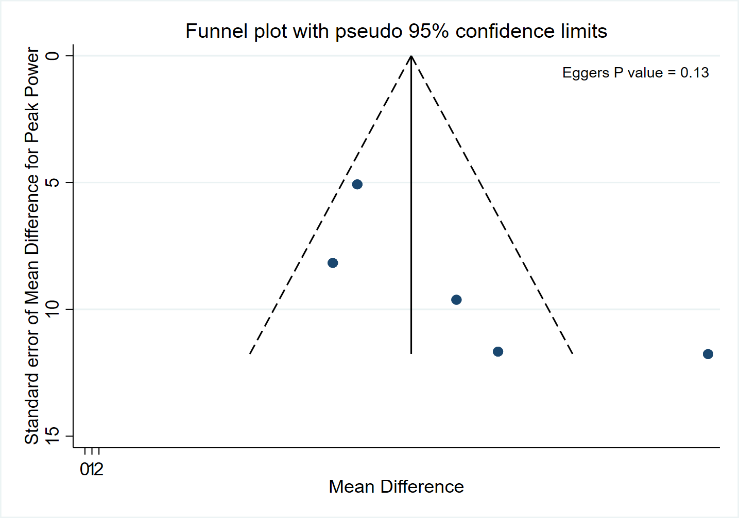  Peak Power/Peak work load |

**References**

1. Bell JW, Chen D, Bahls M, Newcomer SC. Evidence for greater burden of peripheral arterial disease in lower extremity arteries of spinal cord-injured individuals. Am J Physiol Heart Circ Physiol. 2011;301(3):H766-72. doi:<https://dx.doi.org/10.1152/ajpheart.00507.2011>

2. Bhambhani YN, Burnham RS, Wheeler GD, Eriksson P, Holland LJ, Steadward RD. Ventilatory threshold during wheelchair exercise in untrained and endurance-trained subjects with quadriplegia. Adapted Physical Activity Quarterly. 1995;12(4):333-43. doi:10.1123/apaq.12.4.333

3. Brenes G, Dearwater S, Shapera R, LaPorte RE, Collins E. High density lipoprotein cholesterol concentrations in physically active and sedentary spinal cord injured patients. Arch Phys Med Rehabil. 1986;67(7):445-50.

4. Buchholz AC, Martin Ginis KA, Bray SR, et al. Greater daily leisure time physical activity is associated with lower chronic disease risk in adults with spinal cord injury. Appl Physiol Nutr Metab. 2009;34(4):640-7. doi:<https://dx.doi.org/10.1139/H09-050>

5. Currie KD, West CR, Hubli M, Gee CM, Krassioukov AV. Peak heart rates and sympathetic function in tetraplegic nonathletes and athletes. Med Sci Sports Exerc. 2015;47(6):1259-64. doi:10.1249/MSS.0000000000000514

6. Currie KD, West CR, Stohr EJ, Krassioukov AV. Left Ventricular Mechanics in Untrained and Trained Males with Tetraplegia. Journal of neurotrauma. 2017;34(3):591-8. doi:10.1089/neu.2016.4510

7. Dallmeijer AJ, Hopman MT, van der Woude LH. Lipid, lipoprotein, and apolipoprotein profiles in active and sedentary men with tetraplegia. Arch Phys Med Rehabil. 1997;78(11):1173-6.

8. Dallmeijer AJ, Hopman MT, Angenot EL, van der Woude LH. Effect of training on physical capacity and physical strain in persons with tetraplegia. Scand J Rehabil Med. 1997;29(3):181-6.

9. Davis GM, Shephard RJ. Cardiorespiratory fitness in highly active versus inactive paraplegics. … and science in sports and exercise. 1988.

10. G DER, Matos-Souza JR, Costa ESAD, et al. Physical activity and improved diastolic function in spinal cord-injured subjects. Med Sci Sports Exerc. 2014;46(5):887-92. doi:10.1249/MSS.0000000000000187

11. Dearwater SR, LaPorte RE, Robertson RJ, Brenes G, Adams LL, Becker D. Activity in the spinal cord-injured patient: an epidemiologic analysis of metabolic parameters. Med Sci Sports Exerc. 1986;18(5):541-4.

12. D'Oliveira GL, Figueiredo FA, Passos MC, Chain A, Bezerra FF, Koury JC. Physical exercise is associated with better fat mass distribution and lower insulin resistance in spinal cord injured individuals. J Spinal Cord Med. 2014;37(1):79-84. doi:10.1179/2045772313Y.0000000147

13. Flank P, Fahlstrom M, Bostrom C, Lewis JE, Levi R, Wahman K. Self-reported physical activity and risk markers for cardiovascular disease after spinal cord injury. Journal of rehabilitation medicine. 2014;46(9):886-90. doi:10.2340/16501977-1857

14. Rimmer JH, Riley BB, Rubin SS. A new measure for assessing the physical activity behaviors of persons with disabilities and chronic health conditions: the Physical Activity and Disability Survey. American journal of health promotion : AJHP. 2001;16(1):34-42. doi:10.4278/0890-1171-16.1.34

15. Washburn RA, Zhu W, McAuley E, Frogley M, Figoni SF. The physical activity scale for individuals with physical disabilities: development and evaluation. Arch Phys Med Rehabil. 2002;83(2):193-200. doi:10.1053/apmr.2002.27467

16. Hubner-Wozniak E, Morgulec-Adamiec N, Malara M, Okecka-Szymanska J. EFFECT OF TRAINING ON THE SERUM LIPID PROFILE IN ABLE-BODIED AND SPINAL CORD INJURED RUGBY PLAYERS. Biol. Sport. 2010;27(4):269-72.

17. Hubner-Wozniak E, Morgulec-Adamowicz N, Malara M, Lewandowski P, Okecka-Szymanska J. Effect of rugby training on blood antioxidant defenses in able-bodied and spinal cord injured players. Spinal Cord. 2012;50(3):253-6. doi:<https://dx.doi.org/10.1038/sc.2011.134>

18. Huonker M, Schmid A, Sorichter S, Schmidt-Trucksab A, Mrosek P, Keul J. Cardiovascular differences between sedentary and wheelchair-trained subjects with paraplegia. Med Sci Sports Exerc. 1998;30(4):609-13. doi:10.1097/00005768-199804000-00020

19. Koury JC, Passos MC, Figueiredo FA, Chain A, Franco JG. Time of physical exercise practice after injury in cervical spinal cord-injured men is related to the increase in insulin sensitivity. Spinal Cord. 2013;51(2):116-9. doi:<https://dx.doi.org/10.1038/sc.2012.85>

20. Lovell D, Shields D, Beck B, Cuneo R, McLellan C. The aerobic performance of trained and untrained handcyclists with spinal cord injury. European Journal of Applied Physiology. 2012;112(9):3431-7. doi:10.1007/s00421-012-2324-x

21. Maggioni MA, Ferratini M, Pezzano A, et al. Heart adaptations to long-term aerobic training in paraplegic subjects: an echocardiographic study. Spinal Cord. 2012;50(7):538-42. doi:10.1038/sc.2011.189

22. Craig CL, Marshall AL, Sjöström M, et al. International physical activity questionnaire: 12-country reliability and validity. Med Sci Sports Exerc. 2003;35(8):1381-95. doi:10.1249/01.Mss.0000078924.61453.Fb

23. Paim LR, Schreiber R, de Rossi G, et al. Circulating microRNAs, Vascular Risk, and Physical Activity in Spinal Cord-Injured Subjects. J Neurotrauma. 2019;36(6):845-52. doi:10.1089/neu.2018.5880

24. Sadowska-Krepa E, Zwierzchowska A, Glowacz M, Borowiec-Rybak K, Klapcinska B. Blood metabolic response to a long-term wheelchair rugby training. Spinal Cord. 2016;54(5):371-5. doi:<https://dx.doi.org/10.1038/sc.2015.178>

25. Schreiber R, Paim LR, de Rossi G, et al. Matrix metalloproteinases and left ventricular function and structure in spinal cord injured subjects. Clinica chimica acta; international journal of clinical chemistry. 2014;437:136-40. doi:10.1016/j.cca.2014.07.018

26. Schreiber R, Souza CM, Paim LR, et al. Impact of Regular Physical Activity on Adipocytokines and Cardiovascular Characteristics in Spinal Cord-Injured Subjects. Arch Phys Med Rehabil. 2018;99(8):1561-7.e1. doi:10.1016/j.apmr.2018.02.010

27. Schumacher YO, Ruthardt S, Schmidt M, Ahlgrim C, Roecker K, Pottgiesser T. Total haemoglobin mass but not cardiac volume adapts to long-term endurance exercise in highly trained spinal cord injured athletes. Eur J Appl Physiol. 2009;105(5):779-85. doi:10.1007/s00421-008-0963-8

28. Ingles M, Serra-Ano P, Gambini J, et al. Active paraplegics are protected against exercise-induced oxidative damage through the induction of antioxidant enzymes. Spinal Cord. 2016;54(10):830-7. doi:<https://dx.doi.org/10.1038/sc.2016.5>

29. Zwiren LD, Bar Or O. Responses to exercise of paraplegics who differ in conditioning level. Medicine and Science in Sports and Exercise. 1975;7(2):94-8.

30. Wecht JM, Marsico R, Weir JP, Spungen AM, Bauman WA, De Meersman RE. Autonomic recovery from peak arm exercise in fit and unfit individuals with paraplegia. Medicine and Science in Sports and Exercise. 2006;38(7):1223-8. doi:10.1249/01.mss.0000227306.34149.ba

31. Tanhoffer RA, Tanhoffer AI, Raymond J, Hills AP, Davis GM. Exercise, energy expenditure, and body composition in people with spinal cord injury. Journal of physical activity & health. 2014;11(7):1393‐400. doi:10.1123/jpah.2012-0149

32. Lee YH, Oh KJ, Kong ID, Kim SH, Shinn JM. Effect of regular exercise on cardiopulmonary fitness in males with spinal cord injury: ncbi.nlm.nih.gov; 2015.

33. Martin Ginis KA, van der Scheer JW, Latimer-Cheung AE, et al. Evidence-based scientific exercise guidelines for adults with spinal cord injury: an update and a new guideline. Spinal Cord. 2018;56(4):308-21. doi:10.1038/s41393-017-0017-3

34. Evans N, Wingo B, Sasso E, Hicks A, Gorgey AS, Harness E. Exercise Recommendations and Considerations for Persons With Spinal Cord Injury. Arch Phys Med Rehabil. 2015;96(9):1749-50. doi:10.1016/j.apmr.2015.02.005

35. Haskell WL, Lee IM, Pate RR, et al. Physical activity and public health: updated recommendation for adults from the American College of Sports Medicine and the American Heart Association. Med Sci Sports Exerc. 2007;39(8):1423-34. doi:10.1249/mss.0b013e3180616b27

36. Hubli M, Currie KD, West CR, Gee CM, Krassioukov AV. Physical exercise improves arterial stiffness after spinal cord injury. Journal of Spinal Cord Medicine. 2014;37(6):782-5. doi:<https://dx.doi.org/10.1179/2045772314Y.0000000232>

37. Nightingale TE, Walhin JP, Thompson D, Bilzon JL. Biomarkers of cardiometabolic health are associated with body composition characteristics but not physical activity in persons with spinal cord injury. J Spinal Cord Med. 2017;42(3):328-37. doi:10.1080/10790268.2017.1368203

38. Rio C. Lipid profile of spinal cord injury: Paraplegia and sports. Arquivos de Fisiatria e Doencas Osteo-Articulares. 1997;4(2):71-8.

39. Akkurt H, Karapolat HU, Kirazli Y, Kose T. The effects of upper extremity aerobic exercise in patients with spinal cord injury: a randomized controlled study. Eur J Phys Rehabil Med. 2017;53(2):219-27. doi:<https://dx.doi.org/10.23736/S1973-9087.16.03804-1>

40. Davis G.M, Shephard R.J, H. LFH. Cardiac effects of short term arm crank training in paraplegics: echocardiographic evidence. Eur J Appl Physiol 1987; 56:90-6.

41. De Almeida PA, Barbosa VRC, Dias MC, Neiva CM. The effects of adaptated swimming program on the biochemstry blood profile and physical fitness in individuals with spinal cord injury. Medicina. 2011;44(4):377-88.

42. Gorgey AS, Martin H, Metz A, Khalil RE, Dolbow DR, Gater DR. Longitudinal changes in body composition and metabolic profile between exercise clinical trials in men with chronic spinal cord injury. Journal of Spinal Cord Medicine. 2016;39(6):699-712. doi:10.1080/10790268.2016.1157970

43. Hicks AL, Martin KA, Ditor DS, et al. Long-term exercise training in persons with spinal cord injury: effects on strength, arm ergometry performance and psychological well-being. Spinal Cord. 2003;41(1):34-43. doi:10.1038/sj.sc.3101389

44. Kim DI, Lee H, Lee BS, Kim J, Jeon JY. Effects of a 6-Week Indoor Hand-Bike Exercise Program on Health and Fitness Levels in People With Spinal Cord Injury: A Randomized Controlled Trial Study. Arch Phys Med Rehabil. 2015;96(11):2033-40 e1. doi:10.1016/j.apmr.2015.07.010

45. Kim DI, Taylor JA, Tan CO, et al. A pilot randomized controlled trial of 6-week combined exercise program on fasting insulin and fitness levels in individuals with spinal cord injury. Eur Spine J. 2019;28(5):1082-91. doi:10.1007/s00586-019-05885-7

46. Nightingale TE, Walhin JP, Thompson D, Bilzon JLJ. Impact of Exercise on Cardiometabolic Component Risks in Spinal Cord-injured Humans. Med Sci Sports Exerc. 2017;49(12):2469-77. doi:10.1249/mss.0000000000001390

47. Nightingale TE, Rouse PC, Walhin JP, Thompson D, Bilzon JLJ. Home-Based Exercise Enhances Health-Related Quality of Life in Persons With Spinal Cord Injury: A Randomized Controlled Trial. Arch Phys Med Rehabil. 2018;99(10):1998-2006 e1. doi:10.1016/j.apmr.2018.05.008

48. Ordonez FJ, Rosety MA, Camacho A, et al. Arm-cranking exercise reduced oxidative damage in adults with chronic spinal cord injury. Arch Phys Med Rehabil. 2013;94(12):2336-41. doi:10.1016/j.apmr.2013.05.029

49. Rosety-Rodriguez M, Camacho A, Rosety I, et al. Low-grade systemic inflammation and leptin levels were improved by arm cranking exercise in adults with chronic spinal cord injury. Arch Phys Med Rehabil. 2014;95(2):297-302. doi:10.1016/j.apmr.2013.08.246

50. Totosy de Zepetnek JO, Pelletier CA, Hicks AL, MacDonald MJ. Following the Physical Activity Guidelines for Adults With Spinal Cord Injury for 16 Weeks Does Not Improve Vascular Health: A Randomized Controlled Trial. Arch Phys Med Rehabil. 2015;96(9):1566-75. doi:10.1016/j.apmr.2015.05.019

51. Hopman MT, Dallmeijer AJ, Snoek G, van der Woude LH. The effect of training on cardiovascular responses to arm exercise in individuals with tetraplegia. Eur J Appl Physiol. 1996;74(1-2):172-9.

52. Lavado EL, Cardoso JR, Silva LG, Dela Bela LF, Atallah AN. Effectiveness of aerobic physical training for treatment of chronic asymptomatic bacteriuria in subjects with spinal cord injury: a randomized controlled trial. Clin Rehabil. 2013;27(2):142-9. doi:10.1177/0269215512450522

53. Pelletier CA, Totosy de Zepetnek JO, MacDonald MJ, Hicks AL. A 16-week randomized controlled trial evaluating the physical activity guidelines for adults with spinal cord injury. Spinal Cord. 2015;53(5):363-7. doi:10.1038/sc.2014.167

54. Nightingale TE, Rouse PC, Walhin JP. Home-based exercise enhances health-related quality of life in persons with spinal cord injury: A randomized controlled trial: Elsevier; 2018.

55. Huonker M, Schmid A, Sorichter S, Schmidt-Trucksab A, Mrosek P, Keul J. Cardiovascular differences between sedentary and wheelchair-trained subjects with paraplegia. Med Sci Sports Exerc. 1998;30(4):609-13.

**ONLINE APPENDIX I.** Detailed search strategies used in this review

**Search date: April 16^th^ 2021**

|  | Before deduplication | After deduplication |
| --- | --- | --- |
| Embase.com | 3994 |  |
| Medline Ovid | 2383 |  |
| Cochrane Library | 643 |  |
| Web-of-Science | 2502 |  |
| Google Scholar | 200 |  |
| Total | 9722 | **5816** |

3906 duplicate records have been removed

**Embase.com = 3994 results**

('spinal cord injury'/exp OR 'cervical spine injury'/de OR 'spinal cord ischemia'/de OR 'paraplegia'/de OR 'spastic paraplegia'/de OR 'quadriplegia'/de OR 'spinal dysraphism'/de OR ('injury'/exp AND 'spinal cord'/exp) OR (((spine or spinal) NEAR/3 (injur* or trauma* or damag*)) OR (spinal cord NEAR/3 (disease* or contusion* or laceration* or transection* or lesion* or trauma* or ischemi* or ischaemi*)) OR (myelopath* NEAR/3 (trauma* or post-trauma* or posttrauma*)) OR ((spine or spinal or vertebrae) NEAR/3 (fracture* or trauma* or injur* or damage* or wound*)) OR 'central cord injury syndrome*' OR 'central cord syndrome*' OR 'central spinal cord syndrome*' OR 'cauda equine syndrome*' OR 'anterior cord syndrome*' OR 'conus medullaris syndrome*' OR 'Brown Sequard' OR paraplegi* or quadriplegi* or tetraplegi* OR 'spina bifida'):ti,ab)

**AND**

('physical activity'/exp OR 'exercise'/exp OR 'sport'/exp OR 'training'/de OR 'fitness'/de OR 'endurance'/de OR 'athlete'/exp OR 'leisure time physical activity'/de OR 'leisure time exercise questionnaire'/de OR (exercis* OR (physical* NEAR/3 (activ* OR fit* OR exert* OR inactiv*)) OR sport* OR athlete* OR non-athlete* OR fitness OR training OR trained OR untrained OR (activ* NEAR/3 lifestyle*) OR sedentary):ab,ti)

**OR**

(paralympic* OR para-athlet* OR parathlet* OR para-sport*):ab,ti

**AND**

('diabetes mellitus'/exp OR 'cardiovascular disease'/de OR 'heart failure'/de OR 'congestive heart failure'/de OR 'heart disease'/de OR 'cardiovascular risk'/exp OR 'cardiometabolic risk'/de OR 'blood pressure'/exp OR 'hypertension'/de OR 'coronary artery disease'/de OR 'ischemic heart disease'/exp OR 'cerebrovascular accident'/de OR 'venous thromboembolism'/de OR 'atherosclerotic cardiovascular disease'/de OR 'brain ischemia'/exp OR 'insulin response'/exp OR 'glucose blood level'/exp OR 'insulin blood level'/exp OR 'hyperinsulinism'/exp OR 'impaired glucose tolerance'/de OR 'impaired fasting glucose'/de OR 'hyperglycemia'/de OR 'fasting glucose'/de OR 'glycosylated hemoglobin'/exp OR 'lipid blood level'/exp OR 'lipid level'/exp OR 'C reactive protein'/de OR 'c reactive protein blood level'/de OR (inflammation/de AND (marker/de OR 'C reactive protein'/exp OR cytokine/de OR fibrinolysis/exp OR 'tumor necrosis factor alpha'/exp)) OR 'chronic inflammation'/exp OR atherosclerosis/de OR 'atherosclerotic plaque'/de OR 'carotid atherosclerosis'/exp OR 'coronary artery atherosclerosis'/exp OR 'obesity'/de OR 'body mass'/de OR 'abdominal obesity'/de OR 'waist circumference'/de OR 'metabolic disorder'/exp OR 'oxidative stress'/de OR 'reactive oxygen metabolite'/de OR 'lipid peroxidation'/de OR 'isoprostane derivative'/de OR 'malonaldehyde'/de OR 'lipoxygenase'/de OR 'myeloperoxidase'/de OR 'endothelium derived relaxing factor'/de OR 'nitric oxide'/de OR 'prostacyclin derivative'/exp OR 'endothelium derived hyperpolarizing factor'/de OR 'endothelium derived constricting factor'/de OR 'endothelin 1'/de OR 'intercellular adhesion molecule 1'/de OR 'vascular cell adhesion molecule 1'/de OR 'endothelial leukocyte adhesion molecule 1'/de OR 'PADGEM protein'/de OR 'arterial stiffness'/de OR 'heart function'/exp OR 'cardiovascular function'/exp OR 'flow-mediated dilation test'/de OR 'peripheral arterial tonometry'/de OR 'heart stroke volume'/de OR (diabet* OR prediabet* OR pre-diabet* OR HbA1c OR HbA-1c OR ((cardiovascular OR coronar* OR cardiac OR heart OR cardiometabol* OR cardio-metabol* OR metabolic) NEAR/3 (disease* OR event* OR disorder* OR health)) OR cvd OR cvds OR CV-risk OR 'blood pressure*' OR hypertension OR ((ischemi* OR ischaemi* OR fail* OR insufficien* OR infarct*) NEAR/3 (heart OR cardia* OR myocard*)) OR (cerebrovascular* NEAR/3 accident*) OR cva OR stroke* OR cardiopath* OR angina OR ((brain OR cerebral) NEAR/3 (ischemi* OR ischaemi*)) OR ((glucose OR sugar OR insulin* OR lipid* OR cholester* OR lipoprotein* OR triacylglycerol* OR triglyceride*) NEAR/6 (level* OR blood OR serum OR plasma* OR concentration*)) OR hyperglycemi* OR hyperglycaemi* OR dyslipidemia* OR dyslipidaemia* OR glucosaem* OR glucosem* OR glycaem* OR glycem* OR hyperinsulin* OR hypoinsulin* OR insulinaem* OR insulinem* OR (insulin NEAR/3 (response OR dependen* OR resistan* OR sensitiv*)) OR hypercholesterol* OR (inflammat* NEAR/3 (chronic* OR marker* OR biomarker* OR interleukin* OR crp OR 'c reactive' OR cytokine* OR leptin* OR fibrinolys* OR fibrinogenlys* OR 'tumor necrosis factor' OR tnf)) OR atheroscler* OR arterioscler* OR homocysteine* OR obes* OR adipos* OR (waist NEAR/3 (circumference* or hip)) OR 'body mass' OR 'body size' OR 'body weight' OR bmi OR (oxidative NEAR/3 stress*) OR (reactive NEAR/3 oxygen* NEAR/3 (metabolite* OR species)) OR (lipid* NEAR/3 (peroxidat* OR autooxidat* OR autoxidat*)) OR lipoperoxidat* OR lipo-peroxidat* OR isoprostan* OR malonaldehyde* OR lipoxygenase* OR myeloperoxidase* OR 'endothelial-derived relaxing factor' OR 'nitric oxide' OR prostaglandin-i2 OR PGI2 OR 'endothelium derived hyperpolarising factor*' OR 'endothelium derived constricting factor*' OR 'endothelium derived contracting factor*' OR endothelin-1 OR 'intercellular adhesion molecule-1' OR 'vascular cell adhesion molecule-1' OR E-selectin OR selectin-E OR P-selectin OR selectin-P OR ((vascular OR arterial OR aortic) NEAR/1 (stiffness OR stiffening)) OR ((arterial) NEAR/1 (wall OR thickness)) OR ((ventricular OR ventricle OR heart) NEAR/3 (function* OR dysfunction* OR rate*)) OR 'ventricle stroke volume' OR 'pulse wave' OR 'flow-mediated dilatation' OR 'peripheral arterial tonometry' OR echocardiograph*):ab,ti)

**OR** ('cardiorespiratory fitness'/de OR 'cardiorespiratory endurance'/de OR 'cardiopulmonary fitness'/de OR 'oxygen consumption'/exp OR 'maximal oxygen uptake'/de OR 'metabolic equivalent'/de OR 'peak work rate'/de OR 'exercise physiology'/de OR 'exercise test'/exp OR 'ergospirometry'/de OR (((cardiorespir* OR cardiopulmonar* OR 'cardio respir*' OR 'cardio pulmonar*' OR aerobic) NEAR/3 (fitness OR capacit* OR endur*)) OR ((exercise) NEAR/3 (test* OR toleran* OR physiolog*)) OR ((oxygen OR O2) NEAR/3 (consumption* OR uptak* OR demand* OR requir* OR utilizat*)) OR 'Vo2 max' OR Vo2max OR 'max Vo2' OR maxVo2 OR 'Vo2 peak' OR Vo2peak OR 'peak Vo2' OR peakVo2 OR 'peak work rate*' OR WRpeak OR (metabolic* NEAR/1 equivalent*) OR METS OR 'MET value*' OR ergospirometr* OR spiroergometr* OR spiroergograph* ergospirograph*):ab,ti)

**NOT** ([animals]/lim NOT [humans]/lim) NOT ([Conference Abstract]/lim OR [Letter]/lim OR [Note]/lim OR [Editorial]/lim)

**Medline Ovid = 2028 results**

(exp Spinal Cord Injuries/ OR exp Spinal Cord Ischemia/ OR exp Paraplegia/ OR Quadriplegia/ OR Spinal Dysraphism/ OR (((spine or spinal) ADJ3 (injur* or trauma* or damag*)) OR (spinal cord ADJ3 (disease* or contusion* or laceration* or transection* or lesion* or trauma* or ischemi* or ischaemi*)) OR (myelopath* ADJ3 (trauma* or post-trauma* or posttrauma*)) OR ((spine or spinal or vertebrae) ADJ3 (fracture* or trauma* or injur* or damage* or wound*)) OR central cord injury syndrome* OR central cord syndrome* OR central spinal cord syndrome* OR cauda equine syndrome* OR anterior cord syndrome* OR conus medullaris syndrome* OR Brown Sequard OR paraplegi* OR quadriplegi* OR tetraplegi* OR spina bifida).ab,ti.)

**AND**

(exp Exercise/ OR exp Sports/ OR Endurance Training/ OR High-Intensity Interval Training/ OR Resistance Training/ OR exp Physical Fitness/ OR Athletes/ OR Leisure Activities/ OR (exercis* OR (physical* ADJ3 (activ* OR fit* OR exert* OR inactiv*)) OR sport* OR athlete* OR non-athlete* OR fitness OR training OR trained OR untrained OR (activ* ADJ3 lifestyle*) OR sedentary).ab,ti.)

**OR**

(paralympic* OR para-athlet* OR parathlet* OR para-sport*).ab,ti.

**AND**

(exp "Diabetes Mellitus"/ OR "Cardiovascular Diseases"/ OR "Heart Failure"/ OR "Heart Diseases"/ OR exp Blood Pressure/ OR exp "Coronary Artery Disease"/ OR exp "Myocardial Ischemia"/ OR exp "Stroke"/ OR Venous Thromboembolism/ OR Hypertension/ OR "Atherosclerosis"/ OR exp "Brain Ischemia"/ OR "Insulin Resistance"/ OR glucose/bl OR insulin/bl OR exp Hyperinsulinism/ OR exp Hyperglycemia/ OR lipids/bl OR Glycated Hemoglobin A/ OR C-Reactive Protein/OR (inflammation/ AND (biomarkers/ OR "C-Reactive Protein"/ OR cytokines/ OR fibrinolysis/ OR "Tumor Necrosis Factor-alpha"/)) OR Atherosclerosis/ OR "Plaque, Atherosclerotic"/ OR "Carotid Artery Diseases"/ OR exp obesity/ OR "Body Mass Index"/ OR Waist Circumference/ OR Metabolic Diseases/ OR Oxidative Stress/ OR Reactive Oxygen Species/ OR Lipid Peroxidation/ OR Isoprostanes derivative/ OR Malondialdehyde/ OR Lipoxygenase/ OR Peroxidase/ OR Endothelium-Dependent Relaxing Factors/ OR Nitric Oxide/ OR exp prostaglandins i/ OR Endothelin-1/ OR Nitrites/ OR Cell Adhesion Molecules/ OR Intercellular Adhesion Molecule-1/ OR Vascular Cell Adhesion Molecule-1/ OR e-selectin/ OR p-selectin/ OR Vascular Stiffness/ OR Pulse Wave Analysis/ OR exp Heart Function Tests/ OR Ventricular Dysfunction/ OR (diabet* OR prediabet* OR pre-diabet* OR HbA1c OR HbA-1c OR ((cardiovascular OR coronar* OR cardiac OR heart OR cardiometabol* OR metabolic) ADJ3 (disease* OR event* OR disorder* OR health)) OR cvd OR cvds OR CV-risk OR blood pressure* OR ((ischemi* OR ischaemi* OR fail* OR insufficien* OR infarct*) ADJ3 (heart OR cardia* OR myocard*)) OR (cerebrovascular* ADJ3 accident*) OR cva OR stroke* OR cardiopath* OR angina OR ((brain OR cerebral) ADJ3 (ischemi* OR ischaemi*)) OR ((glucose OR sugar OR insulin* OR lipid* OR cholester* OR lipoprotein* OR triacylglycerol* OR triglyceride*) ADJ6 (level* OR blood OR serum OR plasma* OR concentration*)) OR hyperglycemi* OR hyperglycaemi* OR dyslipidemia* OR dyslipidaemia* OR glucosaem* OR glucosem* OR glycaem* OR glycem* OR hyperinsulin* OR hypoinsulin* OR insulinaem* OR insulinem* OR (insulin ADJ3 (response OR dependen* OR resistan* OR sensitiv*)) OR hypercholesterol* OR (inflammat* ADJ3 (chronic* OR marker* OR biomarker* OR interleukin* OR crp OR c reactive OR cytokine* OR leptin* OR fibrinolys* OR fibrinogenlys* OR tumor necrosis factor OR tnf)) OR atheroscler* OR arterioscler* OR homocysteine* OR obes* OR adipos* OR (waist ADJ3 (circumference* or hip)) OR body mass OR body size OR body weight OR bmi OR (oxidative ADJ3 stress*) OR (reactive ADJ3 oxygen* ADJ3 (metabolite* OR species)) OR (lipid* ADJ3 (peroxidat* OR autooxidat* OR autoxidat*)) OR lipoperoxidat* OR lipo-peroxidat* OR isoprostan* OR malonaldehyde* OR lipoxygenase* OR myeloperoxidase* OR endothelial-derived relaxing factor OR nitric oxide OR prostaglandin-i2 OR PGI2 OR endothelium derived hyperpolarising factor* OR endothelium derived constricting factor* OR endothelium derived contracting factor* OR endothelin-1 OR intercellular adhesion molecule-1 OR vascular cell adhesion molecule-1 OR E-selectin OR selectin-E OR P-selectin OR selectin-P OR ((vascular OR arterial OR aortic) ADJ1 (stiffness OR stiffening)) OR ((arterial) ADJ1 (wall OR thickness)) OR ((ventricular OR ventricle OR heart) ADJ3 (function OR dysfunction OR rate)) OR ventricle stroke volume OR pulse wave OR flow-mediated dilatation OR peripheral arterial tonometry OR echocardiograph*).ab,ti.)

**OR**

(Cardiorespiratory Fitness/ OR exp Oxygen Consumption/ OR Metabolic Equivalent/ OR exp Exercise Test/ OR Exercise Tolerance/ OR (((cardiorespir* OR cardiopulmonar* OR cardio-respir* OR cardio-pulmonar* OR aerobic) ADJ3 (fitness OR capacit* OR endur*)) OR ((exercise) ADJ3 (test* OR toleran* OR physiolog*)) OR ((oxygen OR O2) ADJ3 (consumption* OR uptak* OR demand* OR requir* OR utilizat*)) OR Vo2 max OR Vo2max OR max Vo2 OR maxVo2 OR Vo2 peak OR Vo2peak OR peak Vo2 OR peakVo2 OR peak work rate* OR WRpeak OR (metabolic* ADJ1 equivalent*) OR METS OR MET value* OR ergospirometr* OR spiroergometr* OR spiroergograph* OR ergospirograph*).ab,ti.)

**NOT** (exp animals/ NOT humans/) NOT (letter OR news OR comment OR editorial OR congress OR abstracts).pt.

**Cochrane CENTRAL = 545 results***Cochrane Central Register of Controlled Trials Issue 7 of 12,* April 2021

((((spine or spinal) NEAR/3 (injur* or trauma* or damag*)) OR (spinal cord NEAR/3 (disease* or contusion* or laceration* or transection* or lesion* or trauma* or ischemi* or ischaemi*)) OR (myelopath* NEAR/3 (trauma* or post-trauma* or posttrauma*)) OR ((spine or spinal or vertebrae) NEAR/3 (fracture* or trauma* or injur* or damage* or wound*)) OR "central cord injury syndrome" OR "central spinal cord syndrome" OR "central cord syndrome" OR "cauda equine syndrome" OR "anterior cord syndrome" OR "conus medullaris syndrome" OR "Brown Sequard" OR paraplegi* or quadriplegi* or tetraplegi* OR "spina bifida"):ab,ti,kw)

**AND**

((exercis* OR (physical* NEAR/3 (activ* OR fit* OR exert* OR inactiv*)) OR sport* OR athlete* OR non-athlete* OR fitness OR training OR trained OR untrained OR (activ* NEAR/3 lifestyle*) OR sedentary):ab,ti,kw)

**OR**

(paralympic* OR para-athlet* OR parathlet* OR para-sport*):ab,ti,kw

**AND**

((diabet* OR prediabet* OR pre-diabet* OR HbA1c OR HbA-1c OR ((cardiovascular OR coronar* OR cardiac OR heart OR cardiometabol* OR cardio-metabol* OR metabolic) NEAR/3 (disease* OR event* OR disorder* OR health)) OR cvd OR cvds OR CV-risk OR "blood pressure" OR hypertension OR ((ischemi* OR ischaemi* OR fail* OR insufficien* OR infarct*) NEAR/3 (heart OR cardia* OR myocard*)) OR (cerebrovascular* NEAR/3 accident*) OR cva OR stroke* OR cardiopath* OR angina OR ((brain OR cerebral) NEAR/3 (ischemi* OR ischaemi*)) OR ((glucose OR sugar OR insulin* OR lipid* OR cholester* OR lipoprotein* OR triacylglycerol* OR triglyceride*) NEAR/5 (level* OR blood OR serum OR plasma* OR concentration*)) OR hyperglycemi* OR hyperglycaemi* OR dyslipidemia* OR dyslipidaemia* OR glucosaem* OR glucosem* OR glycaem* OR glycem* OR hyperinsulin* OR hypoinsulin* OR insulinaem* OR insulinem* OR (insulin NEAR/3 (response OR dependen* OR resistan* OR sensitiv*)) OR hypercholesterol* OR (inflammat* NEAR/3 (chronic* OR marker* OR biomarker* OR interleukin* OR crp OR "c reactive" OR cytokine* OR leptin* OR fibrinolys* OR fibrinogenlys* OR "tumor necrosis factor" OR tnf)) OR atheroscler* OR arterioscler* OR homocysteine* OR obes* OR adipos* OR (waist NEAR/3 (circumference* or hip)) OR "body mass" OR "body size" OR "body weight" OR bmi OR (oxidative NEAR/3 stress*) OR (reactive NEAR/3 oxygen* NEAR/3 (metabolite* OR species)) OR (lipid* NEAR/3 (peroxidat* OR autooxidat* OR autoxidat*)) OR lipoperoxidat* OR lipo-peroxidat* OR isoprostan* OR malonaldehyde* OR lipoxygenase* OR myeloperoxidase* OR "endothelial-derived relaxing factor" OR "nitric oxide" OR prostaglandin-i2 OR PGI2 OR "endothelium derived hyperpolarising factor" OR "endothelium derived constricting factor" OR "endothelium derived contracting factor" OR endothelin-1 OR "intercellular adhesion molecule-1" OR "vascular cell adhesion molecule-1" OR E-selectin OR selectin-E OR P-selectin OR selectin-P OR ((vascular OR arterial OR aortic) NEAR/1 (stiffness OR stiffening)) OR ((arterial) NEAR/1 (wall OR thickness)) OR ((ventricular OR ventricle OR heart) NEAR/3 (function* OR dysfunction* OR rate*)) OR "ventricle stroke volume" OR "pulse wave" OR "flow-mediated dilatation" OR "peripheral arterial tonometry" OR echocardiograph*):ab,ti,kw)

**OR**

((((cardiorespir* OR cardiopulmonar* OR cardio-respiratory OR cardio-pulmonary OR aerobic) NEAR/3 (fitness OR capacit* OR endur*)) OR ((exercise) NEAR/3 (test* OR toleran* OR physiolog*)) OR ((oxygen OR O2) NEAR/3 (consumption* OR uptak* OR demand* OR requir* OR utilizat*)) OR "Vo2 max" OR Vo2max OR "max Vo2" OR maxVo2 OR "Vo2 peak" OR Vo2peak OR "peak Vo2" OR peakVo2 OR "peak work rate" OR "peak work rates" OR WRpeak OR (metabolic* NEAR/1 equivalent*) OR METS OR (MET NEXT value*) OR ergospirometr* OR spiroergometr* OR spiroergograph* OR ergospirograph*):ab,ti,kw)

**Web of Science = 2119 results**

TS=(((((spine or spinal) NEAR/2 (injur* or trauma* or damag*)) OR (spinal cord NEAR/2 (disease* or contusion* or laceration* or transection* or lesion* or trauma* or ischemi* or ischaemi*)) OR (myelopath* NEAR/2 (trauma* or post-trauma* or posttrauma*)) OR ((spine or spinal or vertebrae) NEAR/2 (fracture* or trauma* or injur* or damage* or wound*)) OR "central cord injury syndrome" OR "central spinal cord syndrome" OR "central cord syndrome" OR "cauda equine syndrome" OR "anterior cord syndrome" OR "conus medullaris syndrome" OR "Brown Sequard" OR paraplegi* or quadriplegi* or tetraplegi* OR "spina bifida")))

TS=(((exercis* OR (physical* NEAR/2 (activ* OR fit* OR exert* OR inactiv*)) OR sport* OR athlete* OR non-athlete* OR fitness OR training OR trained OR untrained OR (activ* NEAR/2 lifestyle*) OR sedentary)))

TS=((paralympic* OR para-athlet* OR parathlet* OR para-sport*))

TS=(((diabet* OR prediabet* OR pre-diabet* HbA1c OR HbA-1c OR ((cardiovascular OR coronar* OR cardiac OR heart OR cardiometabol* OR cardio-metabol* OR metabolic) NEAR/2 (disease* OR event* OR disorder* OR health)) OR cvd OR cvds OR CV-risk OR "blood pressure" OR hypertension OR ((ischemi* OR ischaemi* OR fail* OR insufficien* OR infarct*) NEAR/2 (heart OR cardia* OR myocard*)) OR (cerebrovascular* NEAR/2 accident*) OR cva OR stroke* OR cardiopath* OR angina OR ((brain OR cerebral) NEAR/2 (ischemi* OR ischaemi*)) OR ((glucose OR sugar OR insulin* OR lipid* OR cholester* OR lipoprotein* OR triacylglycerol* OR triglyceride*) NEAR/6 (level* OR blood OR serum OR plasma* OR concentration*)) OR hyperglycemi* OR hyperglycaemi* OR dyslipidemia* OR dyslipidaemia* OR glucosaem* OR glucosem* OR glycaem* OR glycem* OR hyperinsulin* OR hypoinsulin* OR insulinaem* OR insulinem* OR (insulin NEAR/2 (response OR dependen* OR resistan* OR sensitiv*)) OR hypercholesterol* OR (inflammat* NEAR/2 (chronic* OR marker* OR biomarker* OR interleukin* OR crp OR "c reactive" OR cytokine* OR leptin* OR fibrinolys* OR fibrinogenlys* OR "tumor necrosis factor" OR tnf)) OR atheroscler* OR arterioscler* OR homocysteine* OR obes* OR adipos* OR (waist NEAR/2 (circumference* or hip)) OR "body mass" OR "body size" OR "body weight" OR bmi OR (oxidative NEAR/2 stress*) OR (reactive NEAR/2 oxygen* NEAR/2 (metabolite* OR species)) OR (lipid* NEAR/2 (peroxidat* OR autooxidat* OR autoxidat*)) OR lipoperoxidat* OR lipo-peroxidat* OR isoprostan* OR malonaldehyde* OR lipoxygenase* OR myeloperoxidase* OR "endothelial-derived relaxing factor" OR "nitric oxide" OR prostaglandin-i2 OR PGI2 OR "endothelium derived hyperpolarising factor" OR "endothelium derived constricting factor" OR "endothelium derived contracting factor" OR endothelin-1 OR "intercellular adhesion molecule-1" OR "vascular cell adhesion molecule-1" OR E-selectin OR selectin-E OR P-selectin OR selectin-P OR ((vascular OR arterial OR aortic) NEAR/1 (stiffness OR stiffening)) OR ((arterial) NEAR/1 (wall OR thickness)) OR ((ventricular OR ventricle OR heart) NEAR/2 (function* OR dysfunction* OR rate*)) OR "ventricle stroke volume" OR "pulse wave" OR "flow-mediated dilatation" OR "peripheral arterial tonometry" OR echocardiograph*)))

**NOT** TS=(((animal* OR rat OR rats OR mouse OR mice OR murine OR nonhuman* OR primate*) NOT (human* OR patient*)))**AND** DT=(article)

**OR**

TS=((((cardiorespir* OR cardiopulmonar* OR cardio-respir* OR cardio-pulmonar* OR aerobic) NEAR/3 (fitness OR capacit* OR endur*)) OR ((exercise) NEAR/3 (test* OR toleran* OR physiolog*)) OR ((oxygen OR O2) NEAR/3 (consumption* OR uptak* OR demand* OR requir* OR utilizat*)) OR "Vo2 max" OR Vo2max OR "max Vo2" OR maxVo2 OR "Vo2 peak" OR Vo2peak OR "peak Vo2" OR peakVo2 OR "peak work rate*" OR WRpeak OR (metabolic* NEAR/1 equivalent*) OR METS OR ("MET value*") OR ergospirometr* OR spiroergometr* OR spiroergograph* OR ergospirograph*))

**Google scholar (first 200) of 28’000 results**

"Spinal Cord Injury|Injuries|Trauma|Lesion|Ischemia"|Paralympic Exercise|"Physical activity"|Sports|Training diabetes|glucose|cardiovascular|coronary|stroke|obesity|BMI|cholesterol|LDL|HDL|"pulse wave"|"c-reactive"|"cardiorespiratory fitness"|"Vo2"
